# A qualitative analysis of virtual patient descriptions in healthcare education based on a systematic literature review

**DOI:** 10.1186/s12909-016-0655-8

**Published:** 2016-05-13

**Authors:** Inga Hege, Andrzej A. Kononowicz, Daniel Tolks, Samuel Edelbring, Katja Kuehlmeyer

**Affiliations:** Institute for Medical Education, Ludwig-Maximilians-Universität München, Ziemssenstr. 1, München, 80336 Germany; Department of Bioinformatics and Telemedicine, Faculty of Medicine, Jagiellonian University, Kraków, Poland; Department of Learning, Informatics Management and Ethics, Karolinska Institutet, Stockholm, Sweden; Department of Medical and Health Sciences, Linköping University, Linköping, Sweden; Institute for Ethics, History and Theory of Medicine, Ludwig-Maximilians-Universität München, München, Germany; Geisel School of Medicine at Dartmouth, Hanover, NH USA

**Keywords:** Virtual patients, Qualitative content analysis, Concept mapping

## Abstract

**Background:**

Virtual Patients (VPs) have been in the focus of research in healthcare education for many years. The aim of our study was to analyze how virtual patients are described in the healthcare education literature, and how the identified concepts relate to each other.

**Methods:**

We performed a literature review and extracted 185 descriptions of virtual patients from the articles. In a qualitative content analysis approach we inductively-deductively developed categories and deducted subcategories. We constructed a concept map to illustrate these concepts and their interrelations.

**Results:**

We developed the following five main categories: Patient, Teacher, Virtual Patient, Curriculum, and Learner. The concept map includes these categories and highlights aspects such as the under-valued role of patients in shaping their virtual representation and opposing concepts, such as standardization of learner activity versus learner-centeredness.

**Conclusions:**

The presented concept map synthesizes VP descriptions and serves as a basis for both, VP use and discussions of research topics related to virtual patients.

**Electronic supplementary material:**

The online version of this article (doi:10.1186/s12909-016-0655-8) contains supplementary material, which is available to authorized users.

## Background

Virtual Patients (VPs) in healthcare education is a broad umbrella term for computer-based programs to simulate real-life clinical scenarios [1]. A body of research literature reports on didactical and technical VP characteristics, and curricular use. VPs can be realized using a wide range of presentations, styles, and configurations [2]. Variations can include aspects such as interactivity, provision of feedback, curricular integration, or case progression [3]. VPs can be delivered in different formats such as virtual worlds, mainly text-based low-interactive VPs, high-fidelity simulations, or conversational agents. A range of competencies, such as clinical reasoning, communication, or examination skills corresponds to these formats and can be trained with VPs [4].

To further describe VPs, researchers have suggested frameworks and categorizations. For example, Huwendiek et al. developed a typology of VPs based on the four categories general, educational, instructional, and technical [5]. More recently, Talbot et al. developed a classification model that categorizes VPs based on nine categories, (e.g. “core technology” and “learner skills evaluated”) [6], which Kononowicz et al. further elaborated by applying two categories (technology and competency) to classify the body of literature on VPs [4]. Furthermore, VP design principles that students consider beneficial for their learning have been identified (e.g. relevance, interactivity, specific feedback, and authenticity of the interface and student tasks) [7].

The common thread between such frameworks is that they focus on categories and concepts, but not on how these may influence each other or how they are influenced by their environment or actors. However, these aspects have implications on how teacher design VPs and integrate them into courses, and consequently, how students use VPs as learning resources. Therefore, we believe that “zooming out” [8] is important to move forward and elaborate a broader perspective of VPs and their environment filling the gaps between categories with relations. A narrow or limited view focusing on particular VP features may constrain possible holistic learning benefits; an overly visionary and idealistic conceptions considering VPs as “one size fits all” interventions may not be in line with educators’ and students’ way of using VPs.

Our aim was to follow a broad approach by analyzing descriptions of VPs in the healthcare literature, since the process of introducing and explaining a concept often also involves presenting relations of concepts. We aimed to capture and synthesize these concepts and relations to provide both, an overview for educators on using VPs, and a basis for planning research studies with VPs.

We formulated two research questions to guide our work:

- How are virtual patients described in the healthcare literature?
- How do the identified concepts influence each other?

## Methods

The first step of our study was a literature review to extract text passages that describe virtual patients. This included both, explicit definitions and characterizations of virtual patients. In a second step we applied a qualitative content analysis to synthesize and analyze these descriptions.

### Data collection

We searched PubMed, Scopus, EMBASE, PsycINFO, CINAHL/EBSCO, and ERIC for citations on virtual patients (Additional file 1: PRISMA Checklist). Our search strategy consisted of “virtual patient” or “virtual patients” in the title and/or abstract. Exclusion criteria were the following:

- articles in a language other than English.
- short conference abstracts (less than one page).
- “virtual patient” not mentioned within the articles (only in abstract or title).
- non-educational articles.

We deliberately did not include any related search terms, such as case-based learning, since we considered it essential for our study to focus specifically on the concept of virtual patients.

To ensure a comprehensive search in the literature, we did not use a beginning date cutoff and the last date of inclusion was December 31st, 2014.

From the collection of papers, two authors (AK and IH) extracted the VP descriptions into two separate files and composed a single list by consensus. We included statements characterizing the essence or nature of VPs. The descriptions could be composed of multiple text passages from the manuscript, although most of the descriptions originated from introductions. Any specific VP description, such as a specific type of VP implemented at an author’s institution have been excluded.

### Qualitative content analysis

We applied a qualitative content analysis following the approach of Schreier [9] to synthesize and analyze descriptions of virtual patients in the healthcare education literature based on a coding frame we developed for this purpose. The five main categories were developed inductively-deductively; they are stemming from a Simulation-based model developed by Issenberg [10] and our shared understanding of VPs. The categories represent the life-cycle and environment of a VP with the three main actors: the patient, who is in the center and the basis of a VP, the teacher who creates a VP, and the learner who engages with a VP. In addition, we elaborated the VP itself as the learning activity and the curriculum, ie the environment, as main categories. The subcategories were developed in a data-driven approach, for which we used the method of subsumption; three authors (AK, SE, IH) examined the descriptions for relevant concepts and paraphrased them into a subcategory. We reached the point of saturation, after coding 10 % of the descriptions. The final decisions about the subcategories and the point of saturation were made in a discussion (AK, SE, IH). We specified the categories and subcategories with a short description including indicators, examples from the data, and decision rules where necessary.

**Fig. 1:**
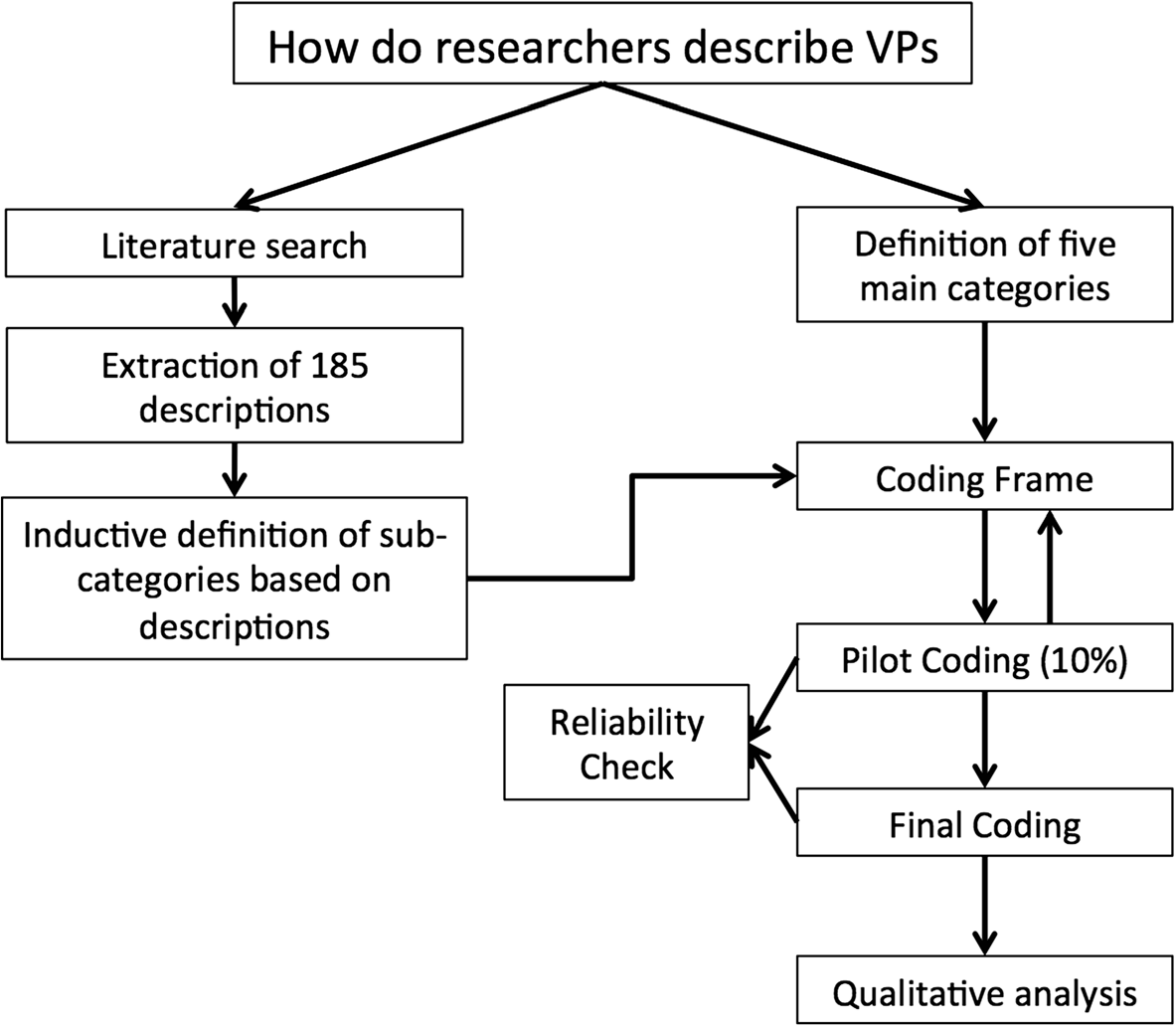
Process of the study

In a second step, we applied the coding frame to 20 % of the descriptions, which were coded independently by two authors (DT, IH). Inconsistencies were resolved by consensus and the coding frame refined to be more specific in some instances. The coding of the remaining 70 % of the descriptions was done by IH; 20 % of these were double-coded by DT and 80 % were re-coded by IH two months after the initial coding. We did not encounter any inconsistencies at this stage. The following flow chart (Fig. 1) illustrates the process of the analysis.

We documented the data analysis in MS Excel and used color codes to segment the descriptions.

### Development of the concept map

We decided to visualize the identified concepts and relations in a concept map. Concept maps are particularly suitable to visualize the organization of knowledge. They represent a set of concepts and their relations in a way that new concepts are linked with what is already known [11]. We used the software CMap [12] to construct a concept map that illustrates the concepts (i.e. categories and subcategories) and the relations between them.

## Results

### Data collection

**Fig. 2:**
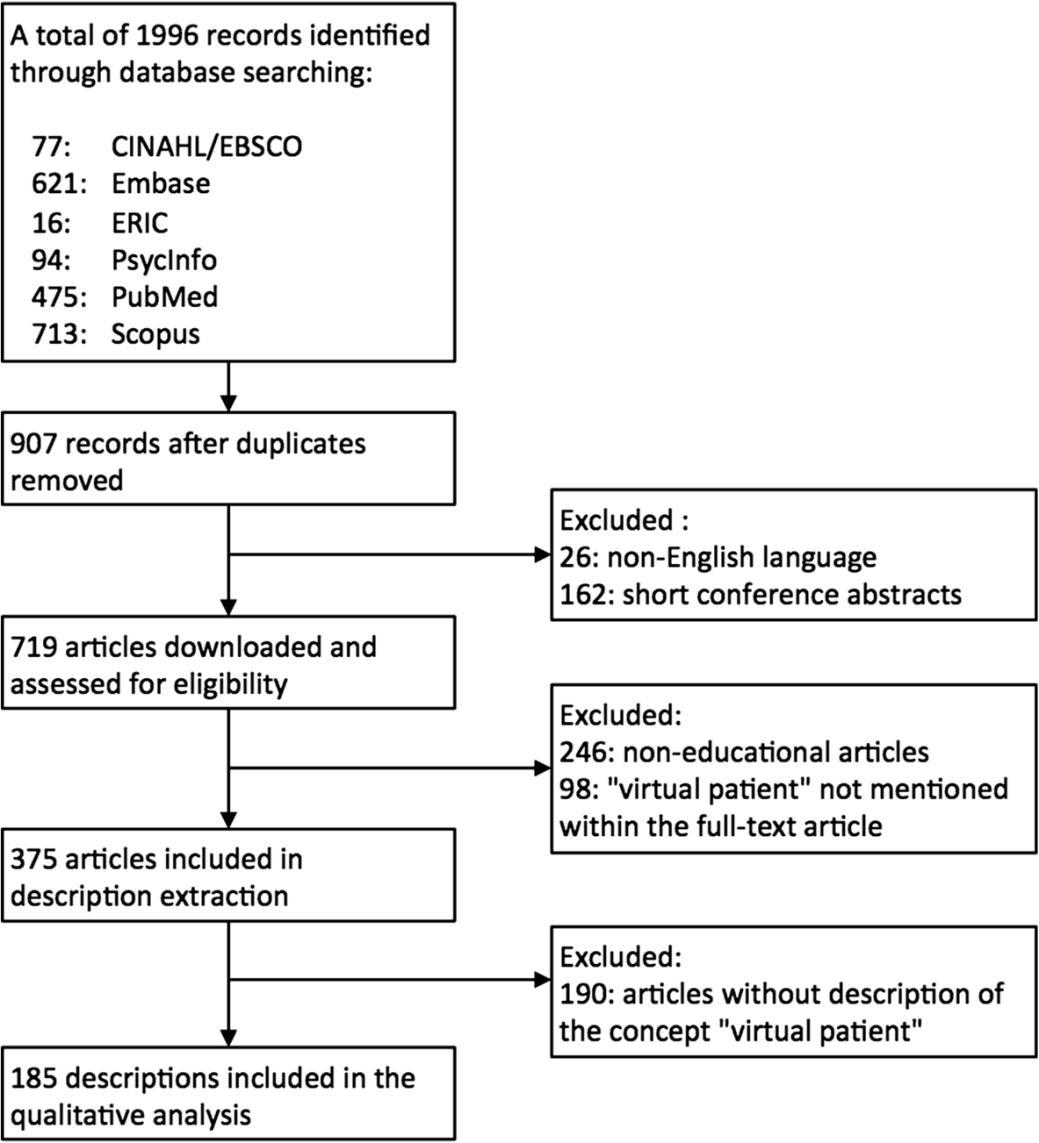
Process applied to identify descriptions

With our search strategy we identified and included 375 educational articles that ranged from 1991 through to the end of 2014 (Fig. 2). From these 375 articles, we extracted 185 descriptions of virtual patients (Additional file 2). The length of the descriptions varied from short paragraphs to page-long descriptions. The remaining 190 articles did not contain any descriptions, mainly because the focus of these articles was not on VPs and therefore the authors did not further describe the VP concept.

### Qualitative content analysis

We defined five main categories: patient, teacher, virtual patient, curriculum, and learner. The subcategories were identified in an inductive approach based on the descriptions. A brief version of the coding guideline is shown in Table 1, the full version can be obtained upon request.

**Table 1:** Categories and subcategories

Category	Subcategories	Definition
Patient		The role the patient plays in a VP
Teacher		The role of the teacher in VP development and use
	Documentation	Tracking and documentation of learner activities and performance
	Resources	Resources required to create and implement VPs
	Challenges	Challenges a teacher might face when creating or using VPs
Virtual Patient		Technical and didactical features of VPs
	Authenticity	Any aspects related to how realistic VPs are
	Interactivity	Any interactive elements implemented in a VP
	Feedback	Any kind of feedback that is implemented in a VP (e.g. from VP, tutor, or peers)
	Variation	Variation and adaptability of VP design
	Technology	Technical aspects of VPs (e.g. scalability or availability).
	Instructional design	Design aspects of a VP (e.g. navigation model)
Curriculum		Relation of a VP to an overall curriculum
	Purpose	Purpose of a VP in a curriculum (e.g. a teaching or assessment activity)
	Integration	Integration of VPs into a curriculum
	Standardization	VPs as a standardization of teaching in medical education
	Adoption	Adoption of VPs in healthcare education
Learner		Learner-related aspects of VPs
	Role-Play	Roles the learner plays within a VP scenario
	Competency	Competencies that can be trained with VPs
	Learner-centeredness	Aspects related to learner as the main focus of a VP activity
	Safe Environment	VPs as a risk-free environment for learners and patients.

Overview of categories and subcategories derived from the VP descriptions

## Patient

The role of patients in the virtual patient creation process was, if at all, described as a deliverer of “authentic video material of real patients” [13] or “(anonymous) patient-related data” [14]. Also, patients were described as not available for bedside-teaching or students do not have access to them (e.g. [15, 16]), which is one of the reasons why VPs (with almost unlimited availability) were seen as a useful supplement to bedside teaching activities.

## Teacher

The role of teacher or educator in the context of VPs was mainly described as the creator of the virtual patients [17, 18].

### Documentation

Teachers can use virtual patient activities to “document the fact that all students have been exposed to all diseases defined by curricular objectives” [14]. VPs “can also easily record student performance and generate reports on individual students” [19].

### Resources/costs to create and maintain a VP

The descriptions included ambiguous information about the resources (i.e. time, effort, and costs) required to develop and deliver VPs. The “process of creating quality VP cases is both expensive and time consuming” [20] and results in “high production costs” [21]. However, in comparison with other teaching activities, authors saw VPs as a cost-effective approach since VPs are “limiting the effort and expense associated with SP [Standardized Patient] training” [22] and “can be delivered at low cost over the internet” [23]. A more differentiated description was provided by Imison et al.: “Branching cases are more difficult to construct, more expensive when compared with linear cases” [24]. As a way to lower the effort and production costs, authors described that “medical schools have undertaken efforts to collaboratively develop and use VPs in the recent past” [25] as well as approaches that “have focused on the exchangeability of virtual patients” [26].

### Challenges

Concerning the challenges of developing and using VPs, mainly legal and technical challenges were mentioned. Legal issues included management of rights, permissions, and copyright issues [27, 28]; technical issues were, for example, management of hard- and software, technology support, unreliable internet connection, difficulty in editing a VP, or cross-platform compatibility [27, 29]. Other challenges were low content validity and reliability [30], difficulty of integration into a curriculum [29], and a non-realistic, impersonal, and isolated learning experience [31].

## Virtual patient

### Authenticity

VPs were described as “real life clinical scenarios” [32] and authenticity was described as “critical to whether a virtual patient can be considered to be part of a situated learning endeavor [..]” [33].

### Interactivity & feedback

VPs “can […] permit a high level of interactivity” and “fall in the high interactivity range of the continuum [34] but “interaction with these systems also varies greatly” [35]. Posel et al. related the level of interactivity to the navigation model of the VP and concluded that “a branching approach allows the highest level of interactivity” [36]. Immediate feedback in VPs can include “visual and auditory feedback” [37] and “virtual patient platforms can also provide real time clinical guidance” [38] and are “giving the learner automatic feedback on the patient management process” [33].

### Variations

This subcategory encompasses aspects of variability, variety, and adaptability within and across VPs. VPs can “demonstrate a variety of clinical or interview scenarios, for example changing the gender or race of the patient” [20]. They can also be “adapt[ed] quickly to prior knowledge and other individual characteristics of learners” [39]. “VPs can take different forms” [40], such as avatars in virtual worlds or text-based formats.

### Technology

To describe the underlying technology of VPs researchers used a great variety of terms such as “computer”, “web-based”, and “simulation”, but also “e-learning”, “virtual reality”, or “game”. Some researchers described the VP technology in more detail, like the use of “multimedia devices such as still images, video, and audio clips” [41] and user input as well as VP output (e.g. text, speech). The use of mobile devices was included in one description as a medium for VPs [42].

The descriptions also included aspects related to availability and accessibility of patients and VPs. Many factors “limit student exposure to real patients; these include reduced patient time in hospitals, increasing hospital specialization and pressure on clinical budgets.” [43]; VPs “can cater to a large number of learners simultaneously and be used by learners repeatedly when needed” [44] “to reach more learners, at more times, in a wider geographic area, than they are able to do through face-to-face contact” [45].

### Instructional design

This subcategory encompasses instructional design aspects of VPs, such as how a learner navigates through a VP. In a linear VP, “the user is prevented from going down any wrong paths by immediate correction” [46]; branched VPs “offer the students various paths to the solution of a case” [14]. However, other terms were used to describe VP navigation, such as “linear-interactive” [47], “knowledge-based contextualization layout” [47], or “algorithm method” [46]. Another categorization of instructional design variations is a problem-solving vs narrative approach. In a problem solving design “information is not ‘cued’, that is, there is no direction from the program format as to what the student’s next course of action should be” [48]; in a narrative design a “personal story line” unfolds following a path in a predefined case [48].

## Curriculum

### Purpose

Three different aspects - learning (including teaching and training), assessment and self-assessment - were pointed out by the researchers: VPs “have been proposed for both training and assessment” [49] and “have also been increasingly utilized for self-assessment” [19].

### Curricular integration

VPs were seen as part of a blended-learning strategy, as “preparation for interaction with SPs and real patients” [22] and to “complement clinical training” [50]. Other potential integration scenarios were the replacement of existing teaching activities with VPs or a learning-by-teaching approach [43, 51]. Curricular integration of VPs was also mentioned as a challenge [52].

### Standardization

Compared to bedside teaching or teaching with standardized patients (SPs), “virtual patients offer true standardization across interactions creating a more consistent but less flexible experience for learners” [53]. “The use of virtual patients can help to standardize the educational value of clinical rotations by exposing all medical students or residents, either through actual clinical cases or through virtual patients, to each classic or important case that is targeted for exposure during the rotation” [45].

### Adoption

Opinions varied about whether VPs are well adopted, an upcoming activity or not (yet) well adopted in healthcare education. Statements ranged from “resulting in lower adoption rates than might be expected” [54], “growth in the use of virtual patients is likely to continue” [39], “VP technology is increasingly used” [55], to VPs are “widely adopted” [56].

## Learner

The target group of VPs were mainly described as students, less often as healthcare professionals. But, also non-healthcare personnel, such as a policemen, fire-fighters [57], and caregiver or family members of a patient [58] were described as potential learners.

### Role-play

VPs “allow students to adopt the role of a health care provider” [59]. This includes professions such as nurses, dentists, pharmacists and clinicians. However, none of the articles described the potential role of the user as a patient or users playing multiple roles including family members in an inter-professional setting.

### Safe environment

VPs provide a “safe environment for students to practice” [60], “practice making clinical decisions in a safe environment without risk to patients” [31], and lead to “improvement of clinical skills in a non-threatening environment” [29]. It is also noteworthy that researchers pointed out the safety aspect for both, patients and learners: “VP provides practice in a safe environment with no risk to patient or student. Mistakes are allowed [40]”.

### Learner-centeredness

Researchers described the use of VPs as “self-paced, independent, and self-directed environment” [34]. VPs “allow for repetitive and deliberate practice” [40] and “there is no time pressure to complete a case, so students may pause, reflect, and choose alternative paths and decisions. [..] Students have the opportunity to repeat their practice and gradually refine their performance” [61].

### Competencies

Competencies that can be addressed by VPs include knowledge acquisition, clinical reasoning, teamwork, communication, and clinical skills training. These competencies are also reflected in the frameworks of Talbot [6] and Kononowicz et al [2]. Additionally, descriptions covered “socio-cultural aspects, trust, respect and empathy” [33].

Researchers pointed out a variety of other characteristics of VPs, such as validity, effectiveness, and reliability of VPs.

### Concept map

**Fig. 3:**
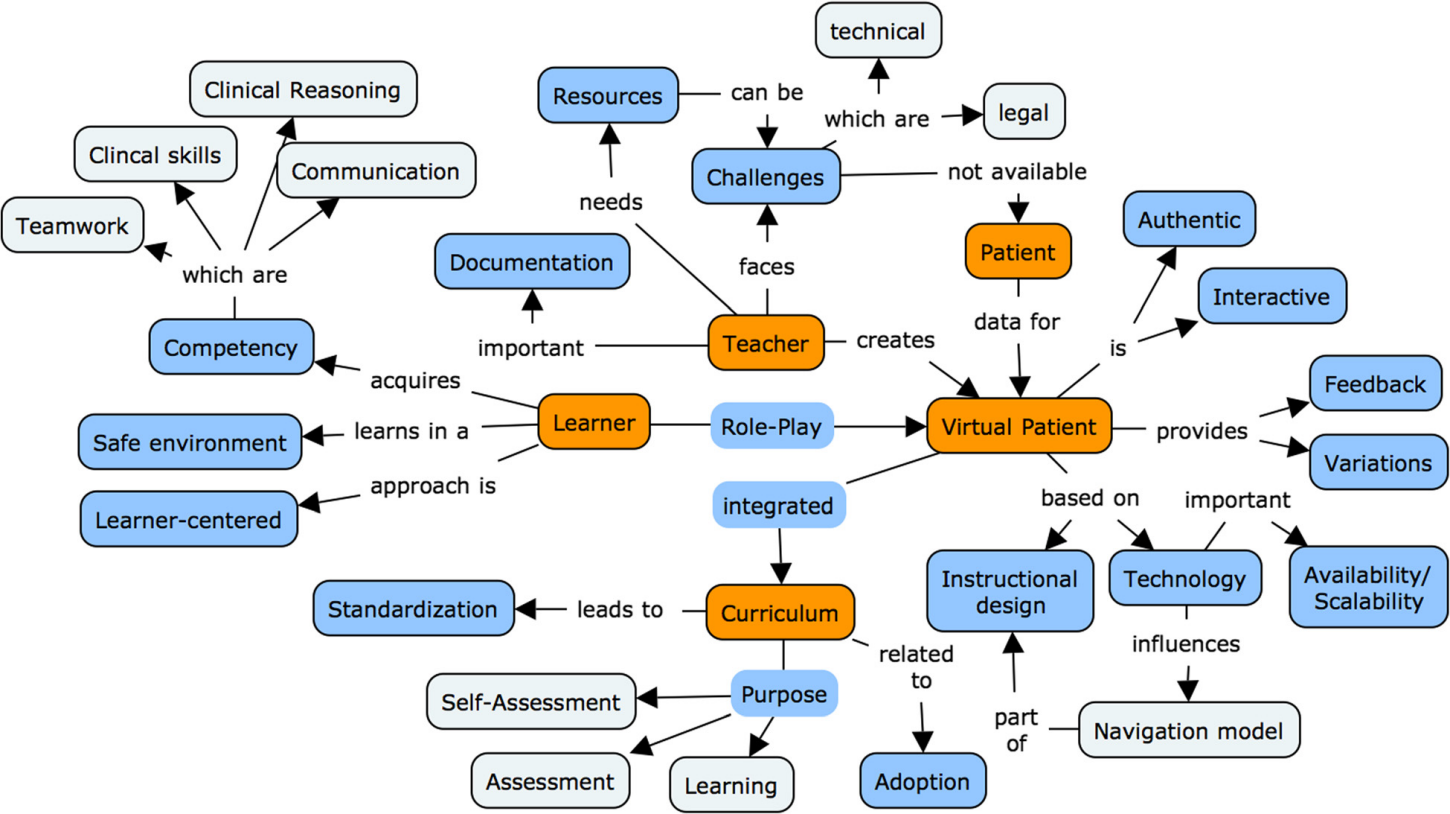
Concept map

Finally, we visualized the relations between the categories and subcategories identified in the analysis in a concept map (Fig. 3).

*Figure*3*shows a concept map of the categories and subcategories. Categories are displayed in orange, subcategories in blue, and related concepts in gray. A full-size map is available under*http://map.virtualpatients.net.

## Discussion

We synthesized VP descriptions from the healthcare education literature into categories and related concepts and visualized these in a concept map (Fig. 3). This map serves as a basis for the following discussion of categories, concepts, relations, and their implications for both, research and medical education practice.

### Patient

Patients are the center and basis of VPs. However, they do not seem to be regarded and valued as significant actors and their role is often one of a passive receiver of care. This is reflected in the lack of subcategories and only a few relations to other concepts in the concept map. But, patients could be much more involved than being mere donors of material. For example, they could act as reviewers or feedback-givers to ensure that their perspective is adequately represented and valued. Especially when thinking of VPs that present a patient’s perspective [33], a deeper inclusion of patients into the VP development seems indispensable. Otherwise, we should be concerned about how much we value learning from and with a patient, a concern also raised by Fitzgerald [62].

Future VP development and research should focus on including patients into the VP development beyond multimedia elements and investigate effects on the perceived authenticity of a VP and learner engagement.

### Teacher

Teachers face time, resource, and technical demands, as well as legal challenges when creating and integrating VPs [20]. On the other hand, VPs are supposed to be more time and cost-efficient than other activities, such as SPs [19]. To reduce costs the group of Berman et al. successfully implemented a model to collaboratively develop VPs and share infrastructure costs among institutions [63]. Another initiative, the Electronic Virtual Patients (eViP) project, focused on the exchangeability of virtual patients among different institutions and across VP systems [43, 64]. Educators would benefit from a sophisticated study investigating the multifaceted costs of VP creation, use, and maintenance depending on the underlying VP technology and initiatives for collaborative development or sharing and repurposing VPs.

Despite such challenges, teachers appreciate the fact that their students VP activities and performances are recorded, documented, and can be evaluated for learner and quality assessment purposes. The documented data (such as time spent on a VP or interactions within a VP) can be used for learning analytics and data mining purposes to support the learning processes. However, this raises new issues about data ownership, privacy issues, and disempowerment of learners [65].

### Virtual patient

We subsumed six different aspects under the virtual patient category: technology, authenticity, interactivity, feedback, instructional design, and variations. Although the research focus most often lies in didactical aspects of VPs, the technical basis and variety, ranging from text-based VPs to high-fidelity simulations, are important characteristics that are included in classification frameworks [4, 5] and influence the VP look. Consequently, VPs are not homogenous technical artifacts and educators should carefully consider which type of VP aligns best with their learning objectives. Further research is needed to classify characteristic features for specific types of VPs. Availability and scalability play an important role in immersive training environments requiring intensive computational resources [66] or in Massive Open Online Courses (MOOC) [67].

Many concepts of the “Virtual Patient” category have been addressed individually in earlier studies and review articles [3, 36] and offer interesting future research questions. For example, the authenticity of a VP (e.g. use of media, interface design, and learner task authenticity) or instructional design have been identified as important [7]. But, how exactly these influence the learning and how learners master the step from virtual to real patients remain open questions [13]. It has to be acknowledged that the aspects of this category are interrelated with other concepts. For example, offering variable and adaptable VPs to learners does not yet seem to be widely implemented [59]. Reasons for this lack of flexibility might be technical limitations, a time-consuming creation process, or a contradiction to standardization efforts. An approach to randomize physiological data to quickly produce many slightly different VPs [68] and a framework to integrate computational models into VPs to produce variations of VPs [42] have been suggested to overcome barriers in the creation process. We recommend further studies to identify methods and assess the quality of such semi-automated created VPs and how this affects the creation process, feedback, and level of interactivity. Future research could also focus on the interplay between these aspects, and how that influences learning and learner engagement.

### Curriculum

When offering VPs to learners it is important to consider how they can be integrated into the overall curriculum. Studies have shown that VPs should not be provided as isolated add-ons to a curriculum [69] and that the integration strategy influences student engagement with the VPs [70]. However, there are interesting open research questions and opportunities for innovation. For example, a long-known blended-learning concept in other content domains, but only recently transferred to medical education, is the flipped or inverted classroom model [71] which can foster critical thinking in students [72]. A few studies [69, 73] have been implemented on how to integrate VPs into such an educational setting but, to our knowledge, a large-scale implementation in the curriculum has not yet been described. However, we see an important future research potential in the integration of VPs into inverted classroom scenarios.

Also, for this category, it is important to be aware of the relations to other concepts when integrating VPs into a curriculum: Standardization and assessment to some extent contradict the aspect of a learner-centered and dynamic VP and also the navigation model of a VP influences the degree of standardization. For example, in branched VPs learners can choose diverse learning paths potentially covering different learning objectives.

Different opinions have been expressed when describing how well VPs have been adopted by the medical education community. This is an interesting side-finding which could be followed-up, for example, by conducting surveys among healthcare educators to identify factors that foster or hinder VP uptake.

### Learner

The learners interacts with the VP in a role-play approach; often they are allowed to make their own clinical decisions from the “driver’s seat” [74]. According to the descriptions, VPs offer a learner-centered and safe environment in which errors have no negative consequences and deliberate practice is fostered. However, curricular aspects also influence the degree of learner-centeredness and safety of the environment. If using VPs as summative or formative assessment tools, errors do have a consequence for the learner - an aspect that should be carefully considered.

Also, when thinking of a learner-centered embedding of VPs into a curriculum, learners could benefit from collecting and connecting content, learning experiences, feedback, or any other VP-related activity in their personal (e-)portfolio. Portfolios have become widespread in healthcare education in the recent years [75] but to our knowledge little development and research has been done on how to effectively combine them with VP activities.

Learner-centeredness could also be reflected by diversity in role-play, for example, by enabling the learner to choose a role (including the patient’s role) in an inter-professional VP setting. The form of the learner’s role differs depending on the type of VP and ranges from a written explanation of the scenario and the user’s role to the learner steering an avatar in a virtual reality. How this aspect influences learning with a VP or perceived authenticity, especially when looking at different role-play levels, remains in our opinion an interesting open research question.

The presented concept map highlights the most prevalent and important topics for the VP community. We encourage researchers and educators to use this map as a basis for designing VPs, developing VP systems, and introducing faculty development courses about virtual patients.

#### Limitations

Our analysis contributes to an increased awareness of VP characteristics. However, there are several limitations to our study. First, there are other, more general, terms used for VPs, such as patient simulation or computer-aided, case-based learning, that have not been included into our analysis. Consequently, we cannot exclude that additional concepts might be revealed when expanding the study to such additional terms. Secondly, our study focused on the body of literature and thereby on the researchers’ and educators’ views. It is unclear to what extent this also represents other perspectives, such as the learners’. Thirdly, we are aware that in qualitative research the subjective perspective of the researchers and the perspectives expressed in the data is interwoven and in retrospective hard to separate.

Finally, as our study aim was to follow a broad approach, we did not present subtle nuances and therefore recommend further, fine-grained research for the subcategories and their relations.

## Conclusions

This paper outlines healthcare education researchers’ descriptions of virtual patients structured in five main categories and related concepts. Many of the concepts, especially in the Virtual Patient category, have been considered in existing frameworks and have been researched upon in the past. However, we also point out aspects that have rarely been investigated, such as documentation, standardization of learning activities, or learner-centeredness in VPs. Further research is needed to explore these concepts in more detail.

In the concept map, we visualize concepts related to VPs and their interrelations. We hope that the map will serve as a dynamic resource for both, educators and researchers, and new concepts and relations will be added as research progresses.

The concepts and relations can serve as a basis for structuring a course, formulating learning objectives, evaluation, quality management, and implementing research studies with VPs. We believe that the interactions and relations of the identified concepts have not yet been fully explored and further research is needed. In particular, learner-centeredness seems to be of central importance and is influenced by aspects, such as standardization or interactivity. Finally, we believe that the involvement of learners and patients in virtual patient development and integration should be prioritized in the future.

### Ethics approval and consent to participate

Not applicable.

### Consent for publication

Not applicable.

### Availability of data and materials

Additional file 2: Extracted definitions (appendix2.pdf).

## Additional files

Additional file 1: PRISMA Checklist. (PDF 123 kb) Additional file 2: Extracted definitions. (PDF 671 kb)
