# Supplementary material for: A qualitative analysis of virtual patient descriptions in healthcare education based on a systematic literature review
Source: BMC Med Educ. 2016 May 13;16:146. doi: 10.1186/s12909-016-0655-8 (PMC4865997; doi:10.1186/s12909-016-0655-8)
Supplement: Additional file 2: — Extracted definitions. (PDF 671 kb) [file 12909_2016_655_MOESM2_ESM.pdf]

Description	Source
As student access to real clinical experience becomes increasingly difficult, the use of virtual patients (VP) has been shown to provide a valid and effective alternative. A virtual patient is "an interactive computer simulation of real-life clinical scenarios for the purpose of medical training, education, or assessment". While many traditional educational artifacts-paper cases, full or partial mannequin simulations, or even live actors simulating patient encounters - are similar to virtual patients in their basic function, the use of computer-simulated virtual patients has posed a challenge with regard to establishing effective standards for their scalable implementation across diverse learning environments. In turn, this challenge becomes all the more pronounced insofar as the embodied experience of professional practice cannot be reproduced online in anything like its full sensorial richness. [...] the evolution of VP technology still remains relatively modest in its scope. While the development and use of virtual patients is not in itself new, the narratives that accompany many current VP scenarios and computer-assisted learning (CAL) sequences remain simple and linear in comparison with the complexity of the everyday clinical environment;	Begg M, Ellaway R Dewhurst D, Macleod, H. Transforming Professional Healthcare Narratives into Structured Game-Informed-Learning Activities. Innovate: Journal of Online Education 2007; 3(6).
Virtual patients as a form of educational intervention can take many forms and can provide highly effective ways of addressing reduced student access to real patients, the need for standardised and well-structured educational patient encounters, and opportunities for students to practice in safe and responsive environments. However, virtual patients can also be complicated<br>and costly to develop. As a result collaborative and distributed development is best suited to their widespread take up. [..] Virtual patients can take many forms; software-based physiological simulators, simulated patients in the form of actors (used for instance in OSCE stations or skills training sessions) and physical manikins and simulators, all of which can be used in education and all of which are well described elsewhere. In education, at least, there is a growing consensus around the virtual patient as 'an interactive computer simulation of real-life clinical scenarios for the purpose of healthcare and medical training, education or assessment'. Virtual patients are typically presented as pre-existing cases that the student works through, typically on their own but they can also be collaborative or cases they build up themselves. Virtual patient activities may be about solving a problem (such as diagnosis or treatment), learning clinical process and thinking, or accessing and learning from a knowledgebase presented in a clinical context. Indeed, despite variations of virtual patients having been around for a while, their application in teaching, learning, and assessment is still being explored. One of the most significant barriers has been difficulties associated with authoring, exchanging and reusing virtual patients. [..] the development and application of common and open standards and specifications is now enabling virtual patients to come into their own as effective and sustainable components of the contemporary health education environment.	Ellaway R, Poulton T, Fors U, McGee JB, Albright S. Building a virtual patient commons. Med Teach 2008;30(2):170-4.
Virtual patient cases are a valuable component of medical education informatics.[...] However, acceptance of the role of virtual patient cases, while necessary, does not help support faculty who are charged with their development. Case creation can be daunting for new authors and there are no specific, discrete guidelines to	Posel N. Making a case: Validating criterion-referenced guidelines for virtual patient case authoring. Dissertation Abstracts International

support these educators. The actual development process of virtual patient case creation can be challenging. [...]virtual patient cases provided students with a requisite domain knowledge-base, cognitive preparation, exposure to clinical reasoning processes, and the opportunity to participate in the ever-accelerating pace of patient care through on-line, but realistic, experiences in the form of virtual patient cases. Aschenbrener, emphasized that these cases encouraged critical thinking, decision making and complex problem solving, and, as “virtual scenarios” permitted understanding of “biomedical concepts, patient diagnosis and management and procedures skills training” [...] diversity of current virtual patient case designs [...]virtual patient cases emphasize active learning. They can (a) permit a high level of interactivity within a personally significant, safe, nonthreatening, self-paced, independent, and self-directed environment, (b) be situated within meaningful real-life practice and support participation in a virtual community of practice, (c) use dynamically constructed learning activities to increase their effectiveness, and (d) provide adapted support of the legitimate peripheral participation of learners of different levels and backgrounds. Virtual patient cases allow learners to plan, act, implement, observe, reflect, and generalize care [...]Virtual patient cases also support the individualization of learning, allowing educators to tailor instruction to the student and to integrate assessment and feedback within relatively realistic and engaging settings. They permit a level of reflective practice that “reallife” clinical exposure might not allow. [...] Virtual patient cases can be stored, retrieved, displayed and archived, thus ensuring availability for reuse, practice, and repurposing [...] Virtual patient cases can provide opportunities for exploration and reflection, permit authors to situate problems of varying complexity in sequenced contexts, present complex problems rooted in real medical experiences, and promote critical evaluation, extend knowledge, and enhance understanding and “deep learning”. They allow for repetitions and deliberate practice in a safe and controlled environment [...]Virtual patient cases can highlight deliberate practice (Ericsson, 2004) and serve to (a) underscore the organizational structure of the medical model, (b) reinforce the individual components of this model such as reason for visit, history, physical examination, laboratory, and radiology test ordering and care management, (c) help learners interactively generate, prioritize, and modify a differential diagnoses or hypotheses, (d) provide learners with multiple and diverse clinical scenarios, (e) require students to use new knowledge or skills to solve problems, and support a deeper understanding of the content and, thus, long-term retention. [..] virtual patient cases allow students to practice these professional approaches and reflect on and compare their performance to [...] Virtual patient cases can be used as activities to further encourage interprofessional dialogue (Posel et al., 2008) and creation of interprofessional care models [...] in her review of both empirical studies and cognitive theories, suggested that virtual patient cases and simulations are “guided discovery forms of e-learning and fall in the high interactivity range of the continuum, [...] They defined a virtual patient as “an interactive computer simulation of real-life clinical scenarios for the purpose of medical training, education or assessment”. More recently, this definition was revised to highlight the utility of virtual patients to provide an effective way to learn, practice and assess clinical	Section A: Humanities and Social Sciences. Vol.73(2-A),2012, pp. 490.

skills. Virtual patients provide the opportunity for a realistic, apprentice-like experience that allows students to practice physician examinations, interviews, and general communication skills, as well as allow students to practice searching for and interpreting data, making clinical decisions, determining a differential diagnosis or hypothesis, and formulating a plan of care or treatment plan.	
Virtual patients (VPs) are “interactive computer simulations of real-life clinical scenarios for the purpose of medical training, education, or assessment”. They offer a wide variety of (anonymous) patient-related data including medical history, physical and technical examinations, as well as laboratory tests. In most cases, the goal of the student is to find the right diagnosis and propose a correct medical treatment based on the data presented. Virtual patients provide a training opportunity in a risk-free environment before students are allowed to take part in bedside teaching. They may also be used to document the fact that all students have been exposed to all diseases defined by curricular objectives. [...] As is often the case with new terms their meaning may vary in the community, which sometimes leads to misunderstandings. For that reason it is imperative to differentiate between the definition of a virtual patient as presented in this chapter and related concepts. Most virtual patients are entirely computer-based and should not be confused with standardised patients – i.e. human actors playing the role of patients, nor with high-fidelity computer simulators connected to realistic robot mannequins. [...]Virtual patients consist of a set of patient-related medical data that can be organised in various forms, thereby allowing its division into different classes of systems. In linear systems the information is displayed in a fixed, predefined order. A user’s decisions do not have an influence on how a case unfolds. Such cases can be created, for instance in the virtual patient system CASUS®. Branched systems offer the students various paths to the solution of a case. The user is confronted with a clinical situation and may select one from a set of options. [...] . Template-based systems (e.g. CAMPUS or Web-SP) offer students a very wide choice of possible options. The user may select from hundreds of interview questions, laboratory exams, physical examination and treatment methods. [..]A recent trend in the authoring of virtual patients is to embed them as 3D-characters in virtual worlds, like for example, Second Life. In such an environment the user may work on the cases collaboratively with fellow students through the Internet. Margaret Bearman attempts to summarise the variety of virtual patient models by dividing them into two major groups: problem-solving and narrative models. In the former a student has to deal with a large set of raw information and has to decide by himself what is relevant. In the latter, a patient's personal storyline is presented. The first model allows more freedom in information collection, whereas the second encourages reflective learning through experience gained in observing the correct medical treatment patterns.	Kononowicz AA, Hege I. Virtual patients as a practical realisation of the e-learning idea in medicine. Safeeullah Soomro (Ed.), ISBN: 978-953-307-092-6, INTECH.
Virtual Patients (VPs), which are interactive computer simulations of real-life clinical scenarios, enable learners to take on the role of the physician, gathering data, diagnosing, and making management decisions. [...] Virtual Patients (VPs), which are interactive computer simulations of real-life clinical scenarios, enable learners to take on the role of the physician, in which they take a history, conduct a physical exam, and make diagnostic and therapeutic decisions	Wilson JI. Evaluating the effectiveness of Virtual Patients to promote clinical reasoning. Dissertation Abstracts International Section A: Humanities and Social Sciences. Vol.72(9-A),2012, pp. 3235.
Online or computer-based patient cases have been a growing area of interest for more than a decade, driven in part	Poulton T, Balasubramaniam C. Virtual patients:

by a number of factors which limit student exposure to real patients; these include reduced patient time in hospitals, increasing hospital specialisation and pressure on clinical budgets. All these factors reduce the amount of time that students have with both patients and clinical educators, and the range of patients that they interact with. In an era of increasing certification, a further issue that may lie ahead is any potential requirement for students to achieve certification of competency before they are exposed to real patients. Virtual patients (VPs), online interactive computer simulations of patient encounters (Ellaway et al. 2006), would seem an ideal tool to address these issues, matching neatly the gradual movement of medical education towards scenariobased and problem-based learning (PBL). VPs are now widely recognised as a valuable teaching tool for medical and healthcare education and assessment. Their benefits include the opportunity to practice clinical problem-solving and clinical management skills (Cook & Triola 2009) and 'the potential to decrease the numbers and effects of medical errors, to facilitate open exchange in training situations to enhance patient safety, and to decrease the reliance on vulnerable patients for training'. [..] increasing impact that VPs have had on the medical curriculum over the last 2 years. [...] One factor has been the development of international collaborative efforts to make VPs more easily available and to introduce standards to make them more interoperable. [..]VPs have become less expensive to produce since the time when a major multi-centre US study established the very high cost of virtual VPs, varying from 10,000 to 130,000 US dollars. VPs have become progressively easier to develop, and major collaborative studies in Europe have suggested that the mean time spent on creation of VPs was now as low as 20–80 h (eViP consortium 2010).[...] The VP authoring systems have in general become easier to use and more accessible to clinicians and subject matter experts over time. [..]As optional components used in self-directed learning, VPs are useful for demonstrating the extent to which students will choose to use resources of this type if they are well presented and perceived as useful to their learning. (more text code from paper)	a year of change. Med Teach 2011;33(11):933-7.
Virtual Patients (VPs), defined as "interactive computer simulations of reallife clinical scenarios" [1] are perfect examples of case-based reusable learning objects, often including a wide range of multimedia content recorded in a medical context. Their primary function is to facilitate and assess the development of clinical reasoning skills.	Kononowicz AA, Zary N, Davies D, Heid J, Woodham L, Hege I. Push and pull models to manage patient consent and licensing of multimedia resources in digital repositories for case-based reasoning. Stud Health Technol Inform. 2011;169:203-7.
Virtual patient (VP) cases have been defined as 'interactive computer simulations of real-life clinical scenarios for the purpose of medical training, education or assessment' . VP cases are particularly valuable for the construction of clinical reasoning skills [...]VP cases can be used to compress time, allowing students to observe strategies such as 'test of time' or 'test of treatment'	Adams EC, Rodgers CJ, Harrington R, Young MD, Sieber VK. How we created virtual patient cases for primary care-based learning. Med Teach 2011;33(4):273-8.
Virtual patient simulations are interactive, educational computer-based games rooted in real-life clinical case scenarios, for use within medical and healthcare training. Suspending disbelief, students take on the role of the healthcare professional and progress through a range of steps in a pre-programmed pathway. Based on continual feedback given during the simulation scenario, care and treatment decisions are made as they would be in the real	Guise V, Chambers M, Conradi E, Kavia S, Välimäki M. Development, implementation and initial evaluation of narrative virtual patients for use in vocational mental health nurse training.

world by applying clinical knowledge and critical thinking skills. Barriers such as time, cost and technical complexity have thus far hindered more widespread development and use of VPs but the recent advent of simpler, less costly development methods has the potential to facilitate increased use of VPs for nursing training.<br>[...]Several different types of VPs exist, involving a variety of associated technical and pedagogical features, case structures and approaches to development. Common VP features that vary by design and available technology include access patterns and mechanisms; level of interactivity. [...]how information is requested and provided; performance tracking; structuring and delivery of feedback; presence or not of learner collaboration; presence, structuring and delivery of assessment; integration or not of multimedia devices such as still images, video and audio clips; and last but not least how case scenarios are structured, developed and maintained . VP scenarios can for example be programmed to unfold in a linear, experimental or branching way, among other types. The format and structure of the scenario has a significant impact on the resulting learning experience .	Nurse Educ Today 2012;32(6):683-9.
A VP is an interactive computer-based simulation of a real-life clinical case scenario, where learners take on the role of a healthcare professional and make judgements and clinical decisions regarding the assessment, diagnoses, treatment and therapeutic care of the VP in the same way they would with a real-life patient based on continual information given during the simulation scenario. Essentially therefore, VP simulations are case-based educational computer games where the user progresses through a range of steps in one or more pre-programmed pathways by applying clinical knowledge and critical thinking skills to make care and treatment decisions. [...] virtual patient (VP) technology, which can be defined as technology that enables and supports interactive computerized clinical simulations.	Guise V, Chambers M, Välimäki M. What can virtual patient simulation offer mental health nursing education? J Psychiatr Ment Health Nurs 2012;19(5):410-8.
Scenario-based gaming can be a powerful tool for teaching reasoning and decision-making skills in competency-based training. Virtual patients (VPs) are an educational game that uses these skills in medicine and healthcare training. [...] A virtual patient is defined as: “an interactive computer simulation of real-life clinical scenarios for the purpose of medical training, education, or assessment. In its simplest form a VP allows the user, usually via a computer, to make a choice based on some presented information. The user is then given feedback dependent on their choice. More complex VPs will offer more choices, and then link pages together, so that the information and choices available at any stage depend on the choices made earlier in the scenario. Although virtual patients address the needs of medicine and healthcare disciplines, they are essentially problem solving exercises – the user must apply knowledge to make decisions and progress through the scenario [...] In order to be successful, VPs need to be as close to real practice as possible, whilst still offering educational opportunities and activities. It is emulating real practice that makes the design of decision-making games such as VPs a challenging task.<br>Simulators and games such as VPs have proven effective and popular e-learning tools that can underpin and extend current practice in teaching and learning. In a survey of 107 medical colleges in Canada and North America, half of virtual patients constructed had cost between 10,000-50,000 dollars, and many cost more than 100,000. In addition, the median time for VP production was 17 months. Not surprisingly, only a few of the medical schools were currently producing them. Within the medical education community, there are four distinct	Conradi E., Poulton T., Round J. Teaching decision-making skills through inexpensive virtual scenarios. Proceedings of the 10th IASTED International Conference on Computers and Advanced Technology in Education: 404-9

approaches to virtual patient design. Approaches to virtual patient design. · The linear approach: The principle of this approach is that the user is prevented from going down any wrong paths by immediate correction. This is often unrealistic for emulating real life, where there are often several ways to tackle a problem and mistakes are often not immediately obvious. This approach might be used for testing knowledge of a protocol, but will not engage students in the same way as more complex, multi choice scenarios. · The algorithm method: Here formulae are developed that mimic physiologic processes in the body and in disease states, so that changes made by the user (typically administration of drugs or fluids) alter the output of the formulae and produce changes on the display, typically of biophysical variables. Some early computer modelled examples were produced at McMaster University, such as MacPuff, MacDope, MacMan. Most of clinical medicine cannot be tackled in this way, as it is descriptive and history based, and has limited transference to other disciplines. · The Lo-Fi method: Here effort is spent on creating a large, but limited number of choices. Users are allowed to make around 2-3 wrong choices sequentially before finding out their mistake. They are given the option, after making a wrong choice, of making the correct choice, as long as the choice was not dangerous. These cases are often not as media-rich or as interactive as hi-fi cases. The interaction will focus on a specific set of options rather than a much broader set of choices. · The Hi-Fi approach: This approach demands a large amount of time, money and effort to model all of the possible choices.<br>Effort is then spent on linking the case to other media and on the appearance of the case. Simulators such as those simulated patients used to train emergency medicine staff and anaesthesiologists are becoming more available, although their purchase price and running costs are large.	
Virtual patients (VPs) are E-learning applications which can be defined as "interactive computer simulations of real-life clinical scenarios for the purpose of medical training, education, or assessment". VPs are an important component of medical curricula and several recent projects, such as Electronic Virtual Patients (eViP) [2] have focused on the exchangeability of virtual patients.	Hege I, Zary N, Kononowicz AA. Criteria to assess the quality of virtual patients. Stud Health Technol Inform. 2012;180:954-7.
Virtual patients (VPs) are a one-of-a-kind e-learning resource where the learner takes the role of a healthcare professional and interactively diagnoses and treats his or her patient. VP approaches usually offer the possibility to embed multimedia such as video and audio clips to illustrate patient findings or procedures. A large variability of designs and approaches has been described. The current literature suggests the main benefit of VPs as primarily promoting clinical reasoning skills. To date VPs have mainly been reported as stand-alone teaching units. [...] Several authors have suggested investigating how to successfully integrate e-learning in general, and VP in particular, into a curriculum. Surprisingly, there are only a few reports available comparing different curricular integration scenarios for VPs. These authors show that providing VPs on its own results in low acceptance and usage, while the approach is more effective when blended with face-to-face sessions. The scarce literature regarding VPs as a preparation tool mainly focuses on the preparation of communication or physical examination skills. The authors of these studies showed that such electronic tools are well accepted among students and that they can improve performance for clinical skills and self-confidence in dealing with real patients. Edelbring et al.	Lehmann R, Bosse HM, Simon A, Nikendei C, Huwendiek S. An innovative blended learning approach using virtual patients as preparation for skills laboratory training: perceptions of students and tutors. BMC Med Educ 2013; 13:23.

reported that VPs can provide a structure in the unstructured environment of a new clinical field for students to get prepared for real patient encounters.	
A VP is defined as: an interactive computer simulation of real-life clinical scenarios for the purpose of medical training, education, or assessment." VPs can take different forms. One form is multimedia VPs where video, audio and computer graphics combine with word-based media. A second is anthropomorphic VPs where virtual agent technology is used to simulate diagnosis and physical treatment as well as human interaction through three dimensional computer models, and highly emotional facial expression and lip-synchronised speech. Both of these have high cost implications and require both sophisticated software and technological expertise. The third form of VP is the word-based VP where a case scenario is presented online and where students are given relevant information about the case scenario in order to make certain decisions about patient care. Word-based VPs can include links to appropriate websites to help the student work through the scenario. [...] A specific type of computer programme that simulates real-life clinical scenarios; learners emulate the roles of health care providers to obtain a history, conduct a physical exam and make diagnostic and therapeutic decisions. In the literature VPs are commonly used as a way of assessing a student's decision-making. In its simplest form a VP allows the user to make clinical decisions based on some clinical information. The user is then given feedback dependent on their choice. VPs are essentially problem-solving and decision-making exercises e the user must apply knowledge to progress through the scenario. If the case is reflective of real situations and real choices, VPs can be useful tools to practice or assess use of knowledge, reasoning, and decision-making skills. Cook and Triola (2009) found that the literature related to the use of VPs in medical education is diverse; [...] Many such studies focus on usability, flexibility and the ability to integrate the VPs into the regular curriculum or on students' attitudes to the use of VPs. Others focus on the use of VPs to solve practical healthcare education problems, e.g., increased student numbers and decreased availability of practice placements or supervisors, or unavailability of suitable real patients. There is additional literature arguing why use of VPs might be valuable in healthcare education and how use of simulated patients are less risky for learning clinical skills than use of real patients. There is also literature offering students' opinions of using VPs as a learning tool without evidence of exactly how the VP influenced students' subsequent practice. Kenny et al. (2008) undertook an evaluative study to ascertain whether the medical students who used the VP activity engaged sufficiently with the VP to ask the appropriate questions of the patient in order to make a diagnosis and propose treatment. Their findings suggest that the interactions between the medical students and the VP resulted in appropriate rapport being demonstrated and that the medical students demonstrated suitable questioning of the VP. However, the extent to which students subsequently took this learning into real practice was not studied. Cook and Triola (2009) concluded that there is an insufficient evidence base related to the use and design of VPs, and their integration into the curriculum, as well as an absence of rigorous studies related to the role of VPs in medical education, how VPs should be designed and presented, how VPs should be integrated with other educational activities, how VPs can be used in assessment and who should develop and maintain VPs.	Hurst HM, Marks-Maran D. Using a virtual patient activity to teach nurse prescribing. Nurse Educ Pract. 2011;11(3):192-8.

Although on-screen ‘‘virtual patients (VPs)’’ have been around for decades it is only now that they are entering the mainstream, and as such they are new to most of the medical education community. There is significant variety in the form, function, and efficacy of different VPs and there is, therefore, a growing need to clarify and distinguish between them [..] A VP has been defined as ‘‘an interactive computer simulation of real-life clinical scenarios for the purpose of healthcare and medical training, education, or assessment. This definition is intentionally broad and inclusive and therefore not without problems, not least because it covers so many different kinds of designs and applications. [...] Returning to the definition of what constitutes a VP, we are immediately faced with a problem: - Interventions and designs that fall within the description of VPs are described using different terms. For instance the following terms (with variations) have all been used to describe designs that are what we would consider to be VPs: ‘‘case-based learning systems’’ (Aha 1991; Garde et al. 2005), ‘‘computer-aided simulations of the clinical encounter’’ (Harless et al. 1971; Melnick 1990), ‘‘interactive patients’’ (Gerritsma & Smal 1988; Hayes & Lehman 1996), ‘‘computerized clinical patient problems’’ (Pickell et al. 1986), ‘‘patient simulation by computer’’ (Verbeek 1987; Clauser et al. 2002), ‘‘patient simulations’’ (Finkelstein et al. 1991; Bergin & Fors 2003) and finally ‘‘VPs’’ (Zary et al. 2006; Begg et al. 2007). . Interventions that may be called VPs but are quite different from the working definition, including: ‘‘simulated patients’’ or ‘‘standardized patients’’ (actors playing the role of patients, for instance in developing communication skills or as part of an Objective Structured Clinical Exam (OSCE) station), physical simulation artefacts (ranging from whole body models such as mannequins, to individual body parts such as task trainers), physiological models (computerized algorithms representing physiological processes), and real patients reflected in the data held about them in health records and other databases. [..] Despite the increasing use of VPs worldwide....	Huwendiek S, De leng BA, Zary N, Fischer MR, Ruiz JG, Ellaway R. Towards a typology of virtual patients. Med Teach. 2009;31(8):743-8.
Virtual patients (VP) are interactive computer simulations of real-life clinical scenarios created for the purpose of medical training, education, or assessment	Kononowicz AA, Heid J, Donkers J, Hege I, Woodham L, Zary N. Development and validation of strategies to test for interoperability of virtual patients. Stud Health Technol Inform. 2009;150:185-9.
Virtual patients are a key exemplar of game–informed learning in medical education, taking a number of different forms, such as artificial patients (typically computer simulations of human physiology, real patients reflected in their data (electronic health records or EHRs), physical simulators (models and mannequins), simulated patients (actors and role–play), and electronic case–studies and scenarios. It is the latter form that has most relevance to e-learning in medicine as ‘an interactive computer simulation of real–life clinical scenarios for the purpose of medical training, education, or assessment'. Typically, virtual patients take the form of an open–ended clinical narrative or a structured patient encounter, the latter being the more common. In either scenario, students may have to search for and/or interpret data, make appropriate clinical decisions or solve particular problems such as making a diagnosis or formulating a treatment regime. Furthermore, the role of the learner may take many forms: the physician or other member of the care team, the patient, or an observer. In addition, they may create a virtual	Ellaway R, Masters K. AMEE Guide 32: e-Learning in medical education Part 1: Learning, teaching and assessment. Med Teach 2008;30(5):455-73

patient themselves, or work through a pre–existing one, they may work alone or collaboratively, they may work through an exemplar case or have to critique a flawed one, and the outcomes may vary between decision–making, knowledge acquisition or assessment. Some virtual patients will employ a case as a framework into which didactic activities are connected while others will encourage open exploration and discovery.[...] Practica, such as simulators and virtual patients, can offer highly valid and authentic learning environments, they can be scalable and replayable, they can be made available on demand, and they can be highly immersive for the learner. Furthermore, by taking a 'thinslicing' approach to learning medical practice, they are particularly useful for managing cognitive load and helping the learners to pace themselves. As such, it is likely that these educational techniques are going to be used as part of patient education as well as for health professionals in the years to come.	
Unlike its siblings, the standardized patients and the highfidelity simulators virtual patient simulation (VPS) systems are fairly recent additions to the spectrum of simulation. VPS can be defined as 'an interactive computer simulation of real-life clinical scenarios for the purpose of healthcare and medical training, education or assessment' and as 'computer programs that simulate real-life clinical scenarios in which the learner acts as healthcare professional obtaining a history and a physical exam and making diagnostic and therapeutic decisions'. The educational applications of VPS are intimately connected to their design (Ellaway et al. 2008). To reach the proposed learning goals most systems take advantage of the linear-interactive, branching or knowledge-based contextualization layout. [...]VP can be used both for learning and for assessment	Botezatu M, Hult H, Tessma MK, Fors UG. Virtual patient simulation for learning and assessment: Superior results in comparison with regular course exams. Med Teach. 2010;32(10):845-50.
The virtual patient (VP) is a web-based tool that allows students to test their clinical decision- making skills using simulated patients. [...] The virtual patient (VP), ''an interactive computer simulation of real-life clinical scenarios for the purpose of healthcare and medical training, education or assessment'', permits the application of clinical reasoning to the care of simulated patients. VPs have been implemented in undergraduate medical education and have been explored in surgical education. [...]The VP can also be used as an assessment tool. High- fidelity simulators and standardized patients are often used for assessment, yet VPs are infrequently implemented for this purpose.	Yang RL, Hashimoto DA, Predina JD, Bowens NM, Sonnenberg EM, Cleveland EC, Lawson C, Morris JB, Kelz RR. The virtual-patient pilot: testing a new tool for undergraduate surgical education and assessment. J Surg Educ. 2013;70(3):394-401.
Virtual patient cases are an increasingly utilized and compelling pedagogical strategy for medical education informatics. They provide educators with the opportunity to develop richly layered, multidimensional teaching situations for their learners. However, 'virtual patients are notoriously difficult to author, adapt and exchange' (MedBiquitous Virtual Patient Specification, Virtual Patient Working Group 2007), and case creation can be daunting. [...] Defined as an 'interactive computer simulation of real-life clinical scenarios for the purpose of health professionals training, education or assessment' in which 'users may be learners, teachers or examiners', virtual patient cases merge the traditional medical emphasis on the value of case-based learning with utilization of new and innovative technologies, and can emphasize 'problem solving (such as diagnosis or treatment), learning a clinical process and thinking or accessing and learning from a knowledgebase presented in a clinical context'. Virtual patient cases provide educators with the opportunity to develop richly layered, multidimensional teaching situations for their learners. Experts in medical and healthcare education suggest that virtual patient cases can (a)	Posel N, Fleiszer D, Shore BM. 12 Tips: Guidelines for authoring virtual patient cases. Med Teach 2009;31(8):701-8.

address the complexities inherent in medical education, (b) promote independent, interactive, self-directed and selfpaced learning , (c) encourage critical thinking, decision-making and complex medical problem-solving, (d) support active learning, understanding and retention of information in long-term memory, (e) facilitate assessment, (f) further individualization and (f) simulate learning in 'realistic' contexts, thus providing access to patient scenarios that are no longer available in the 'clinical lab that is the hospital'.	
Virtual patients (VPs) are computer-based simulations of clinical cases allowing the users to interact with the system and train their clinical reasoning skills. The term "virtual patient" has been referred to various educational tools and methods like simulated patients in the form of actors, software-based physiological simulators, physical manikins and advanced technological simulators [...] There is however a general consensus in considering a VP an "interactive computer simulation of real-life clinical scenarios for the purpose of healthcare and medical training, education or assessment". VPs can provide away to overcome the reduced student access to real patients, aswell as a structured and safe environment for students to practice; they are therefore more and more extensively used in medical education but their efficacy is still a matter of debate. A recent review provided a critical evaluation of relevant literature but it could find mainly descriptive articles, many articles measuring students' satisfaction with VPs but fewstudies providing evidence about the efficacy of VPs. One of the issues raised by Cook in his review was the way in which VPs are integrated in the curriculum. VPs have been used in many different instructional designs and educational applications but not always the terminology used to describe the learning environment and VPs usage was uniform. At this proposal, in her recent paper, Rachel Ellaway proposes to apply activity theory to redefine Virtual patients "from a software artifact to an intrinsic part of an activity that mediates the ways that learners and their objectives interact. The same virtual patient artifact can be used in different learning activities and activity theory identifies each application as discrete and specific to the activity in hand." This was an important theoretical contribution as a model to describe interventions that use VPs in ameaningful and consistent way. Ellaway and Davies (2011) and Huwendiek et al. (2009) work provided categories to identify in a clearer way context of use, objectives and type of interventions for VPs, allowing an easier comparison among different experiments.	Consorti F, Mancuso R, Nocioni M, Piccolo A. Efficacy of Virtual Patients in Medical Education: A Meta-Analysis of Randomized Studies. Computers & Education 2012; 59(3):1001-8.
Educational technologies are used extensively at all points on the medical education continuum and vary widely in complexity, degree of realism, and cost. Such resources include relatively straightforward online multimedia tutorials; high-fidelity virtual patient applications that ask learners to diagnose and manage simulated patients; and immersive, team-based simulations designed around lifelike mannequins.Many institutions have purchased commercial products developed for the medical education market, while others have created inhouse development teams of educators, illustrators, Web designers, programmers, and other multimedia specialists, who create excellent products for their own use.[...] Virtual Patients (VP)—A specific type of computer-based program that simulates real-life clinical scenarios; learners emulate the roles of health care providers to obtain a history, conduct a physical exam, and make diagnostic and ther apeutic decisions. [...] VP: Adv: Encompasses multiple aspects of clinical encounter • Longitudinal and multidisciplinary care lessons • Easy access • Readily customized, Disadv:	Effective use of educational technology in medical Education. Colloquium on Educational Technology:<br>Recommendations and Guidelines for Medical Educators. AAMC Institute for Improving Medical Education 2007.

Limited physical interactivity • Limited fidelity • High production costs[...] Educational Goals: Facilitate basic knowledge acquisition, Improve decision making, Enhance perceptual variation, Practice rare/critical events.	
As the sophistication and availability of educational technology has increased in recent years, the 'virtual patient' has become both viable and pedagogically useful. VPs might be variously defined but, for the purposes of this project, they are what the American Association of Medical Colleges describes as: interactive computer programs that simulate real-life clinical scenarios in which the learner acts as a health care professional obtaining a history and physical exam and making diagnostic and therapeutic decisions. Choules notes that virtual patients generally go beyond what might be referred to as simply 'multimedia-enhanced patients' – such as video of a consultation that models good practice in historytaking – to applications that allow students to consider a full clinical scenario, often in narrative format and with degrees of difficulty that can be adjusted to suit learners' skill level. Such VPs are characteristically media-dense ('high-fidelity'), often incorporating video/animation and artificial intelligence features that allow for interaction with the 'patient', scope for physical examination and investigation of test results – a fitting application of technology for problem-based, authentic learning. There are numerous arguments for including virtual patients in the medical curriculum. As rates of chronic disease in the developed world continue to rise, but outpatient care and shorter hospital admissions become the norm, exemplar cases are less readily available to medical trainees; VPs augment clinical observation and enhance the breadth and consistency of the educational experience. In addition, students report that they appreciate the 'safe' context and structured feedback that virtual patients provide. Almost all comparisons of media-rich VPs show that they are well-received as pedagogic tools, and at least as effective as standard teaching methods. Traditionally virtual patients have been developed either commercially or by clinicians, but this project took a different approach. High-fidelity VPs are both time- and resource-intensive: cost estimates for professionally-created cases range from US$50 000 to over US$100 000 - and the authors' prior experience demonstrates that medical professionals rarely have time to source and finalise virtual patients.	Imison M, Hughes C. The virtual patient project: Using low fidelity, student generated online cases in medical education. ASCILITE 2008 - The Australasian Society for Computers in Learning in Tertiary Education:441-445
Virtual patients have been defined as ''a specific type of computer-based program that simulates real-life clinical scenarios; learners emulate the roles of health care providers to obtain a history, conduct a physical exam, and make diagnostic and therapeutic decisions and comprise a combination of clinical information, digital media, and interactivity. This enables students to work through a clinical case on a computer and derive their own diagnosis and treatment plans while receiving feedback. They are usually created by a team of subjectmatter experts, including clinicians at teaching hospitals, lecturers, and learning technologists. They are increasingly used in medical education to enhance the efficiency in limited clinician teaching time.11 Virtual patients has the advantage of allowing students to practice making clinical decisions in a safe environment without risk to patients while enabling them to refine their clinical reasoning skills.11 Students can access case material off-campus and in their own time, which encourages self-directed learning. As well as complementing the standard caseload, students may have an opportunity to experience unusual or rare cases that they would be unlikely to see in clinics. They can also benefit from using virtual patients to complement traditional face-to-face teaching and enhance small group	Trace C, Baillie S, Short N. Development and preliminary evaluation of student-authored electronic cases. J Vet Med Educ 2012; 39(4):368-74.

problem solving exercises. Virtual patients can be reused or repurposed to suit other areas of the curriculum and can be updated as best practice evolves. There are also concerns that need to be addressed when introducing virtual patient-based teaching including isolation of learners, lack of face–to-face feedback, and the staff resources required for development and subsequent support of faculty and students.12 The cost of developing sophisticated, media-rich virtual patients can be prohibitive and requires a significant investment of clinician and technician time	
Numerous variations of Internet based learning, computer-based simulations, virtual patients, and many other innovations are being used extensively at all levels of training. These modalities both supplement and in some cases replace traditional instruction [...] VPs have been defined as ‘‘a specific type of computer program that simulates real-life clinical scenarios; learners emulate the roles of health care providers to obtain a history, conduct a physical exam, and make diagnostic and therapeutic decisions,’’ Similar to the above study of more generic CAI interventions, research on VPs shows that these are much more effective than no intervention, have small comparative advantages to noncomputer instruction, and suffer from a paucity of research identifying best practices.[...] VPs have been principally designed to foster the development of clinical reasoning skills. [..] VPs can readily provide learners with multiple and varied case examples, and also provide feedback on both the accuracy of the learner's diagnosis and treatment plan, and the information-gathering and decision-making path. Given these characteristics, VPs are increasingly used to accelerate the development of clinical reasoning as part of a spectrum of simulation that may also include more expensive and personnel-intensive activities involving standardized patients, physical simulators, partial task trainers, and more. VPs can also be easily used by groups of learners collaboratively reasoning through a clinical problem (Bryce et al. 1998). Similar to OSCEs, the VP can be used as an assessment tool, one that provides much richer data on a given learner's problem-solving skills [..] For example, interactive media-enhanced Virtual Patients often include authentic video material of real patients, allow large numbers of learners to practice whenever they want, and permit computer-created customized feedback based on individual performance. Implementing a similar program using traditional Standardized Patients would prove difficult and costly in terms of both money and the time of simulated patients, faculty, and learners. [..] Overall this question is difficult to answer given the heterogeneity of VP applications studied to date, and the lack of standardized measures of quality across studies. [...] Fortunately, as technologies improve and commercial or free sources of content and cases become prevalent, the ongoing costs to schools using these advances are likely to decrease dramatically.	Triola MM, Huwendiek S, Levinson AJ, Cook DA. New directions in e-learning research in health professions education: Report of two symposia. Med Teach 2012;34(1):e15-20.
Educators increasingly use virtual patients (computerized clinical case simulations) in health professions training. [...] The computer-screen-based virtual patient, "a specific type of computer program that simulates real- life clinical scenarios; learners emulate the roles of health care providers to obtain a history, conduct a physical exam, and make diagnostic and therapeutic decisions	Cook DA, Erwin PJ, Triola MM. Computerized virtual patients in health professions education: a systematic review and meta-analysis. Acad Med. 2010;85(10):1589-602.
The opposing forces of increased training expectations and reduced training resources have greatly impacted health professions education. Virtual patients (VPs), which take the form of interactive computer-based clinical scenarios, may help to reconcile this paradox. [...] Although definitions vary, we define a VP as a 'specific type of	Cook DA, Triola MM. Virtual patients: a critical literature review and proposed next steps. Med Educ 2009;43(4):303-11.

computer program that simulates real-life clinical scenarios; learners emulate the roles of health care providers to obtain a history, conduct a physical exam, and make diagnostic and therapeutic decisions'. This excludes other forms of computer-based learning in which patient cases do not unfold in response to learner input, and other forms of simulation such as standardised patients (SPs), manikins, part-task trainers and systems requiring specialised equipment. Virtual patients are clinical scenarios that play out on the computer screen. The learner interrogates the patient (the computer) by typing or selecting (or, in some cases, speaking) questions and subsequently requesting information on physical examination findings and laboratory tests. The computer supplies patient responses or other requested information. Learners are typically required to commit to a diagnosis and management plan at some point. Within this framework there is much room for variation (see Table 1 and Appendix S1).1,3 Although all of these variations have potential impact on learning and assessment, perhaps the most important distinctions involve features extraneous to the case itself. As discussed in greater detail below, the selection and sequencing of cases to facilitate deliberate practice4 may matter more than the technical operations of the case itself. Likewise, the manner in which feedback is provided5 and the ways in which...	
Computer-based virtual patients (VPs) are an emerging medium for medical education that addresses barriers faced by geriatrics educators. Research has shown VPs to be as effective in changing knowledge and behavior as more traditional forms of teaching [...] The Association of American Medical Colleges (n.d.) defines virtual patients as "interactive computer programs that simulate real-life clinical scenarios in which the learner acts as a health care professional obtaining a history and physical exam and making diagnostic and therapeutic decisions." Many virtual patient modules provide coaching and feedback functions, which are important aspects of instructional simulations that guide learners as they work through the simulations and remediate or reinforce performance as needed. Virtual patient programs can be designed and developed for Internet delivery. When the virtual patient program contains coaching and feedback functions, this mode of delivery provides anytime, anywhere access to geriatric instruction in a virtual one-on-one bedside teaching scenario. Several learners can simultaneously access the same virtual patient case. Thus, the development and use of e-learning virtual patients can address the shortage of qualified geriatrics educators by enabling the limited number of geriatrician educators to reach more learners, at more times, in a wider geographic area, than they are able to do through face-to-face contact. Virtual patient programs can augment learners' clinical experiences and provide exposure to cases that are not actually encountered on an educational clerkship or rotation because of the variability in patient load and case diversity. The use of virtual patients can help to standardize the educational value of clinical rotations by exposing all medical students or residents, either through actual clinical cases or through virtual patients, to each classic or important case that is targeted for exposure during the rotation. Simulated exposure to clinical cases also allows learners to make mistakes first in a "no stakes" environment. They can learn from their mistakes and see outcomes modeled without harming real patients and their families. This prepares learners for greater participation in actual clinical decision making by increasing confidence and providing necessary cues for higher performance	Orton E, Mulhausen P. E-learning virtual patients for geratric education. Gerontol Geriatr Educ. 2008;28(3):73-88
Virtual patients (VPs) are online representations of clinical cases used in medical education. Widely adopted, they	Bateman J, Allen M, Samani D, Kidd J, Davies

are well placed to teach clinical reasoning skills. International technology standards mean VPs can be created, shared and repurposed between institutions.[...] Virtual patients (VPs) are online representations of clinical cases used in medical education. Widely adopted, they are well placed to teach clinical reasoning skills. [...] Virtual patients (VPs) are computerised representations of realistic clinical cases. Analogous to that on other webbased educational interventions, much of the focus on VP adoption has concerned 'if' rather than 'how' they should be designed and used.3,4 Recent advances in technology and internationally adopted technical standards have potentially changed the definition of what a VP is as VPs can now be shared, edited and repurposed between institutions. Virtual patients have the potential to deliver education to large numbers of students at a relatively low cost, which will be important in addressing the challenges that will face medical education in coming decades.	D. Virtual patient design: exploring what works and why. A grounded theory study. Med Educ 2013: 47: 595–606
Computerised virtual patients (VPs) are increasingly being used in medical education. [...] The VP technology is defined as an interactive computer simulation of real-life clinical scenarios for the purpose of healthcare and medical training, education, or assessment. The educational strength of VPs has been identified foremost as promoting clinical reasoning, an important aspect of clinical learning.[...] Given the wide range of VP design typologies, educational theory alone has limited ability to predict their success and the interplay among them. Although the idea of VPs is not new, they are still regarded as an educational innovation and broad regular use of these has not yet come into practice. In 2005, only 26 of 108 US and Canadian medical schools used VPs, partly explained by high production costs. There is also reason to believe that there are vague conceptions of how the technology should be used to reach its full educational potential.	Edelbring S, Dastmalchi M, Hult H, Lundberg IE, Dahlgren LO. Experiencing virtual patients in clinical learning: a phenomenological study. Adv Health Sci Educ Theory Pract 2011; 16(3):331-45
Virtual patients are used across a variety of clinical disciplines for both teaching and assessment, but are they an appropriate environment in which to develop professional skills.[...] Computer-based virtual patients have been described for training in various health professional fields, including teaching in medical ethics and communication skills. Ellaway describes the virtual patient as 'an interactive computer simulation of real-life clinical scenarios for the purpose of healthcare and medical training, education or assessment'	McEvoy M, Butler B, MacCarrick G. Teaching professionalism through virtual means. Clin Teach. 2012;9(1):32-6.
Virtual patients have been defined as 'interactive computer simulation of clinical scenarios for the purposes of training and assessment'. They have been proposed to be best placed to teach clinical reasoning skills, can be delivered at low cost over the internet, and can be adapted for different specialities, including general practice.	Bateman J, Hariman C, Nassrally M. Virtual patients can be used to teach clinical reasoning. Clin Teach 2012; 9(2):133-4.
Virtual Patients (VPs) are learning systems designed to simulate encounters between a patient and a healthcare professional. VPs may be used throughout the medical curriculum including pre-clinical courses. Virtual Patients are commonly recommended for teaching clinical reasoning and clinical decision making, but have also been used for teaching basic communication skills with patients. There are also suggestions for the use of VPs to emphasize socio-cultural aspects and cultural differences as they pertain to healthcare education. [...] Quality computer-assisted instruction materials are time and labour intensive to develop, and therefore expensive. The development and maintenance of virtual patients in medical education through a collaborative multi-institutional authoring might be the best solution for most medical schools	Muntean V, Calinici T, Tigan S, Fors UG. Language, culture and international exchange of virtual patients. BMC Med Educ 2013; 11;13:21.
Virtual patients (VPs) are used increasingly in medical education as an adjunct to real patients . Virtual patients	Huwendiek S, Reichert F, Bosse HM, de Leng

have been defined as 'interactive computer simulation[s] of real-life clinical scenarios for the purpose of medical training, education, or assessment'. [...]The diversity of VPs and the expense of developing them5 underline the importance of paying careful attention to VP design.	BA, van der Vleuten CP, Haag M, Hoffmann GF, Tönshoff B. Design principles for virtual patients: a focus group study among students. Med Educ. 2009;43(6):580-8
"Virtual patients" are computer-based simulations designed to complement clinical training. These applications possess numerous educational benefits but are costly to develop. Few medical schools can afford to create them. [...] In particular, virtual patients were designed to fill gaps in clerkships by exposing students to diseases that they would not otherwise experience because of short clinical rotations and limited ambulatory care experiences. "Virtual patients" are defined as "computer programs that simulate real-life clinical scenarios in which the learner acts as a health care professional obtaining a history and physical exam and making diagnostic and therapeutic decisions." They should be distinguished from other forms of simulation, such as standardized patients (human actors trained to portray patients) and from high-fidelity simulators (life-sized robot mannequins). Virtual patients can be used to simulate, for example, the longitudinal care of a diabetic patient over the course of nine "virtual" years, condensed into several hours. They expose learners to rare, "do-not-miss" events that might not occur over the course of a three-month clerkship or even a three-year residency, such as a ruptured aortic aneurysm. They permit a window into procedures or conversations in which trainees may not normally participate, such as a cardiac catheterization or a primary care physician giving bad news. They portray a variety of clinical presentations for a single disease such as HIV or, alternatively, multiple diseases for the same clinical presentation, such as chest pain. They have been used to teach topics as diverse as communication skills3 and bioterrorism response. [..] Despite persuasive evidence for the effectiveness of virtual patients, these programs are not ubiquitous in medical education. Because virtual patient programs employ complex programming and multimedia to replicate clinical environments, these resources are extremely time and resource intensive to produce, which is prohibitive to institutions that lack robust educational technology programs. At the same time, virtual patient development tends to be confined within single institutions, resulting in potentially duplicative case development and lack of access for those schools that have not developed virtual patients. In addition, as with any educational innovation, the successful integration and effective use of virtual patient programs hinges on the extent to which educators sufficiently consider curricular issues and training needs. These challenges, among others, have led to the inconsistent and ultimately limited impact of virtual patient simulation on the undergraduate medical curriculum.	Huang G, Reynolds R, Candler C. Virtual patient simulation at US and Canadian medical schools. Acad Med 2007; 82(5):446-51.
Virtual patients have been defined as computer programs that simulate lifelike clinical scenarios in which the learner becomes the health care professional making therapeutic decisions. This technology was first published in the medical literature,8 but has been described in the training of medical, nursing, dental, and other health professionals. Advantages to virtual patient technology include allowing educators to assess application of course content in a ''safe'' environment, while allowing students to both learn and err in private. [...] Branching within the simulation eventually terminates at a session endpoint. Endpoints can reflect positive or negative patient	Benedict N. Virtual patients and problem-based learning in advanced therapeutics. Am J Pharm Educ 2010;74(8):143.

outcomes that likely would have resulted from the collective decisions made by the student. ...	
The virtual patient is a case-based computer program that combines textual information with multimedia elements such as audio, graphics, and animation. It is increasingly being utilized as a teaching modality by medical educators in various fields of instruction. [...] Virtual patients are case-based computer programs that simulate real-life clinical scenarios in which the learner acts as a health care professional obtaining a history and physical exam and making diagnostic and therapeutic decisions. [...] A virtual patient (VP) program may incorporate, to varying degrees, a combination of textual information and other multimedia elements such as audio, graphics, and animation. It can provide clinical information in its entirety, expert modeling through worked examples, and challenging practice dilemmas to be solved by the learner. Through the use of multimedia features such as video, audio, and animations, VP programs can create dynamic learning environments that allow learners to actively delve into realistic case scenarios, obtain contextual information, assess knowledge, gain access to expert modeling of management approaches, and revise management approaches based on adequate signs and support. There are many advantages and disadvantages to consider when using virtual patients (see Table 1). One feature that distinguishes VPs from other learning modalities is its ability to shift the locus of control from instructors to learners through nonlinear access to information, problem identification, on-demand information access, instructional support, and immediate feedback on problem challenges. VPs offer novice medical trainees a realistic, controlled, and nonthreatening environment for deliberate practice of clinical skills. [...] Going beyond its use in teaching, VPs have also been used as an assessment tool.[...] Despite its unique features, VPs should be viewed by educators as an instructional aid and, as such, are generally part of the larger curricula. The Kern model for curriculum development is a useful framework to place VPs in a proper educational context. [...] The implementation of VPs as part of a blended e-learning curriculum that consists of other instructional modalities should be followed by an assessment of the impact of the VP program on learning and an evaluation of the success or lack thereof of this strategy in terms of instructional effectiveness, efficiency, and feasibility. Advantages: Attractive to the “Net generation”, Standardized experience, Meet competencies: K, S, A – clinical reasoning skills, Deliberate Practice, Assessment, Cost-effective?, Efficient delivery: Anytime, anywhere, Learner-centered, Multimedia principles:, Effective?, Scaffold learning tool, Develops skills in educational technology. Disadvantages: Learning curve, Technology requirements: Computer & software, Cost of development: Initial investment, Slow or unreliable Internet connections, Rapidly evolving technology, Cross-platform compatibility, Evolving standards, Issues about ownership, Copyright issues, Maintenance of hardware and software	Tan ZS, Mulhausen PL, Smith SR, Ruiz JG. Virtual patients in geriatric education. Gerontol Geriatr Educ. 2010;31(2):163-73.
recognizing simulated learning experiences—including virtual patients— as equivalents to real-life clinical encounters for accreditation purposes. Although virtual patients offer a more consistent and learner-centered curriculum that provides greater practice opportunities and reduces the demand for busy clinical preceptors, going virtual does involve potential risks [...] Virtual patients are “computer programs that simulate real-life clinical scenarios in which the learner acts as a health care professional obtaining a history and physical exam and making diagnostic and therapeutic decisions. [...]a small total—likely because of the considerable time (16.6 months) and	Tworek J, Coderre S, Wright B, McLaughlin K. Virtual patients: ED-2 band-aid or valuable asset in the learning portfolio? Acad Med. 2010; 85(1):155-8.

cost ($10,000 –$50,000) required to produce each case. At the<br>present time, therefore, virtual patients are still in the realm of the “innovators” and “early adopters,” but their use will likely increase over time, especially since the LCME has granted them curricular parity with real-life clinical experiences: [...] At the curricular level, virtual patients offer several advantages, including greater consistency in the delivery of learning experiences. [...]At the individual student level, virtual patients allow learners—rather than patient availability—to determine their learning agenda. The virtual learning environment is safe because it gives learners permission to fail. They can make errors without patients suffering adverse clinical consequences, and they can err in private. Virtual patients allow learners to have graded challenges, through which they begin with straightforward cases that have a lower cognitive load and then gradually progress to increasingly complex cases. Teaching is always available around virtual patients; the “virtual preceptor” always provides evidence-based recommendations and is never too busy to teach. Finally, when we have a plentiful supply of virtual patients, learners will have a greater opportunity to practice their diagnostic skills. [...] Yes, there are two glaring reasons why virtual patients might not provide a sound learning experience. First, they may not be able to deliver effective feedback, and, second, virtual learning may not transfer to the real-life environment.	
The virtual patient application is a specific type of computer program that simulates real-life clinical scenarios; learners emulate the roles of health care providers to obtain a history, conduct a physical exam, and make diagnostic and therapeutic decisions	Djukic M, Fulmer T, Adams JG, Lee S, Triola MM. NYU3T: teaching, technology, teamwork: a model for interprofessional education scalability and sustainability. Nurs Clin North Am 2012; 47(3):333-46.
An alternative to SPs is use of computer simulation. Virtual-patient (VP) systems are computer programs that simulate real-life clinical scenarios in which the learner can complete a patient interview and physical exam, while making diagnostic and therapeutic decisions. VP systems allow standardized instruction, immediate and objective performance feedback, and unlimited opportunity for repetitive practice. VPs and computer simulations are already used in various branches of medicine to teach communication, counseling, crisis management, procedures, leadership, teamwork, and medical decision-making skills.	Shah H, Rossen B, Lok B, Londino D, Lind SD, Foster A. Interactive virtual-patient scenarios: an evolving tool in psychiatric education. Acad Psychiatry 2012;36(2):146-50.
There are varying proposals for how to define virtual patients. For instance, in proposing a virtual patient typology, Huwendiek and colleagues described it [virtual patients] as “an interactive computer simulation of real-life clinical scenarios for the purpose of healthcare and medical training, education, and assessment. Apart from a number of medical specialties comprising, among others, training in lifesaving events, virtual patients have also been used in other health professions, ranging from nursing6 and occupational therapy to pharmacy practice. [...] Among other purposes, virtual patient technology was designed to promote students’ clinical reasoning and the development of communication skills. Users’ opinions of this educational resource, including medical students’ acceptance of virtual patient design principles and of virtual patient-based learning and assessment tools, indicate that virtual patient technology benefits both clinical reasoning and communication skills. [....] ..the virtual patient technology model definition proposed by Keele University, which defines virtual patient technology as “an	Cavaco AM, Madeira F. European pharmacy students' experience with virtual patient technology. Am J Pharm Educ 2012;76(6):106

interactive learning system that creates a computer-simulated environment of patient real-life scenarios."	
Virtual Patients (VP), interactive computer based programs which simulate real-life clinical scenarios. They are used across a variety of clinical disciplines for both teaching and assessment.	McEvoy MM, Butler B, MacCarrick G, Nicholson AJ. Virtual patients: an effective educational intervention to improve paediatric basic specialist trainee education in the management of suspected child abuse? Ir Med J. 2011 Sep;104(8):250-2.
For the purposes of this specification, a virtual patient is defined as: An interactive computer simulation of real-life clinical scenarios for the purpose of medical training, education, or assessment. Users may be learners, teachers, or examiners. Virtual patients are notoriously difficult and costly to author, adapt and exchange. Historically this has limited their uptake and utility, despite their being able to provide high quality learning opportunities.	MedBiquitous Virtual Patient Player Specifications and Description Document. 2010
... given the diversity of virtual patient simulation systems [...] Virtual patient simulation is a learning method defined as "an interactive computer simulation of real-life clinical scenarios for the purpose of medical training, education, or assessment". This activity focuses on acquiring knowledge and skills with the main objective to develop clinical reasoning and decision-making	Bediang G, Raetzo MA, Geissbuhler A. Virtual patient simulation: a comparison of two approaches for capacity building in Sub-Saharan Africa. Stud Health Technol Inform. 2012;180:978-82.
The demonstration of patient-based cases using automated technology [virtual patients (VPs)] has been available to health science educators for a number of decades. Despite the promise of VPs as an easily accessible and moldable platform, their widespread acceptance and integration into medical curricula have been slow.<br>[...] Despite the fact that the concept has been around for 40 yr, few medical schools have incorporated it into the educational paradigm. In fact, Huang and Candler found that only 24% of medical schools in the United States and Canada were using VPs in their curricula in 2007. Cost is cited as a significant concern as schools struggle to meet a reported average $10,000 –50,000 cost of developing even one VP scenario, not including the associated maintenance costs. However, there is reason to remain optimistic about the use of VPs. They facilitate the provision of feedback and represent a venue for safe and repetitive practice as well as a model where progressive clinical variation and difficulty can be presented. These features offer a significant contribution to the medical curriculum and mirror those observed in other high-fidelity simulation platforms. [...] We refer to VPs using the terminology presented by the American Association for Medical Colleges and echoed by Cook and Triola to be a "specific type of computer program that simulates real-life clinical scenarios; learners emulate the roles of health care providers to obtain a history, conduct a physical exam, and make diagnostic and therapeutic decisions". The main components of VPs include interactivity on the learner's part (as opposed to passively watching videos), the simulation of medical conditions, and the visual and/or physical presentation of the conditions. The manifestation of the VPs can differ greatly and include 1) case studies presented on webpages or CD-ROMs, 2) immersive virtual reality simulations, and 3) robotic human-scale mannequins (Fig. 1). Most systems currently in use	Cendan J, Lok B. The use of virtual patients in medical school curricula. Adv Physiol Educ. 2012;36(1):48-53

represent a linear experience where the user is guided through a scripted dialogue but has the potential to ask questions and receive feedback at each step, although a smaller number of systems allow for any question to be asked at any time in a format that is reminiscent of real conversations. Interaction with these systems also varies greatly. In particular, and of substantial consequence when planning for a curriculum, human-scale mannequins require substantial technical support during activities and great acquisition, storage, and maintenance costs. Simply stated, VPs are a computer-based simulation of a patient and are typically composed of three components: inputs, simulation, and outputs. VP inputs are the interface mechanisms that the learner uses to interact with the system and include standard computer interfaces like a mouse and keyboard. Some VPs require engineered solutions such as force sensors in mannequins. Given the inputs, the VP simulation engine processes and generates a patient response. The simulation is software that attempts to model a component of the patient. The simulation usually relies on an underlying model, such as physiological or pharmacological models, physics, and social/conversational models. The conversational model is the easiest to develop but is dependent on the author of the scenario to provide anticipated outcomes for each possible response, as opposed to a VP that uses a real-time physiological simulation engine. VP outputs present the visual, auditory, and any mechanical output of the simulation results. The VP could be shown as presenting a response (including speaking a response to an input, changing facial expressions, and performing a gesture) along with simulation information (e.g., blood pressure and heart rate). The fidelity and realism of the interaction will vary given the infrastructure (such as computing, space, time, and equipment). The original motivation for the creation of VPs was summarized in an article by McGee et al.. In one of the earliest references to this technology, the authors recognized that multiple nonacademic drivers were conspiring to minimize clinical experiences for students such that a serious gap in clinical experiences was developing. These included the push for higher clinical throughput in academic teaching environments, societal push back to clinical practice on real patients, and higher documentation requirements for procedural skills of all levels, including basic doctoring skills, such as communication. The coincident development of multimedia technology provided a reasonable approach for filling this experience. a computer-based environment. Clinical learning experiences are difficult to standardize and, in the case of conditions that require urgent intervention, nearly impossible to schedule in a reproducible manner. Standardization is difficult both intrainstitutionally and interinstitutionally, and it is not uncommon for students in a given institution to experience a different array of clinical cases and conditions from their peers, making it difficult to guarantee exposure and mastery of concepts. The Liaison Committee on Medical Education recognized this concern with an organized systemwide push for advanced student experience documentation and permission to incorporate simulated patient experiences as “real” experiences. These simulated experiences may include work with standardized patients (SPs). [...] It is worth stating explicitly that VPs could be complementary to SP experiences and not considered as a replacement for SP programs. VPs can provide exactly the same experience repeatedly. VPs are available in simulation centers or continuously online and could provide students with opportunities for repetition, individualized feedback, and opportunities to revisit the actions taken during the interaction, allowing comparison with bestpractices protocols.	

VPs can demonstrate simulated physical examination findings that SPs cannot portray (cardiac murmurs, abnormal breath sounds, neurological findings, etc.). VP interactivity also leverages Kolb and Fry's experiential learning theory, motivating the learner to actively participate in the educational process. By interacting with VPs under certain conditions, it is expected that learning will be more efficient and complete, with a higher retention rate over passive education approaches. This theory presents a cyclical model of learning, consisting of four stages, which tend to follow this sequence: 1) concrete experience ("do"), 2) reflective observation ("observe"), 3) abstract conceptualization ("think"), and 4) active experimentation ("plan"). By presenting clinical variations, VPs add to the general knowledge that a student can draw upon when confronted with a similar case, whether virtual or real, through pattern recognition. Pattern recognition is largely nonanalytical and unconscious and builds upon prior exposure to the clinical concern (2). VPs offer the possibility to address this component of the decision-making infrastructure by adding to the user's clinical foundation and reinforcing knowledge structures (11). [...] We describe this research in further detail below. VPs can also provide students opportunities for self-directed learning, which leads to reflection (5, 6), self-driven change (26, 30), and more insight into performance (13).	
The concept of virtual patients (VPs) encompasses a great variety of predominantly case-based elearning modules with different complexity and fidelity levels. Methods for effective placement of VPs in the process of medical education are sought [...] Virtual patients (VPs) are computer simulations of reallife clinical scenarios created for the purpose of healthcare and medical training, education, or assessment. This concept encompasses a great variety of predominantly case-based e-learning modules with different complexity and fidelity levels	Kononowicz AA, Krawczyk P, Cebula G, Dembkowska M, Drab E, Frączek B, Stachoń AJ, Andres J. Effects of introducing a voluntary virtual patient module to a basic life support with an automated external defibrillator course: a randomised trial. BMC Med Educ 2012;12:41.
Case-based learning objects presenting a patient's story are often called virtual patients (VPs). It should be noticed, however, that the term "virtual patient" (VP) is more general and encompasses all kinds of "interactive computer simulations of real- life clinical scenarios for the purpose of medical training, education, or assessment"	Kononowicz AA, Hege I, Krawczyk P, Zary N. New approaches to linking clinical guidelines to virtual patients. Stud Health Technol Inform. 2012;180:958-62.
A VP can be as simple as a list of demographics and health data on paper or as complex as a computer-generated avatar with whom a student interacts in a program like *Second Life*.4 VPs can also provide a self-paced method with immediate feedback for students to progress, such as in medical/dental history-taking or development of clinical skills. [...] VPs provide many advantages in medical education, including efficiency, efficacy, standard-ization of experiences, interactivity, exposure to rare but critical cases, immediate feedback, and, most importantly, improvement of clinical skills in a non-threatening environment. Disadvantages are cost, difficulty in integrating VPs into curricula, difficulty in editing, technology limitations, and the level of resources required. [...] The advantages of the use of VPs in dental education are clear: 1) reinforcement of basic science information within clinical treatment considerations; 2) uniformity of student experiences; and 3) immediate feedback for learning and self-assessment. VPs that employ interactive components may further evolve to better prepare dental students for their first encounter with a live patient. [...] One suggestion for the definition of a VP might be the following: a digital representation of a realistic patient that is capable of being interactive, i.e., allowing for a	Cederberg RA, Bentley DA, Halpin R, Valenza JA. Use of virtual patients in dental education: a survey of U.S. and Canadian dental schools. J Dent Educ. 2012;76(10):1358-64.

relationship between student and patient. There are also many challenges to the use of VPs in dental education, such as the costs associated with producing a good-quality interactive VP and the commitment of resources to gather the data and create and manage VPs. Additionally, incorporating VP technology into existing courses and providing suitable information technology support to ensure that the technology works are challenges for the use of VPs	
Virtual Patients (VPs) are web-based representations of realistic clinical cases. They are proposed as being an optimal method for teaching clinical reasoning skills. International standards exist which define precisely what constitutes a VP. There are multiple design possibilities for VPs [...] Virtual patients(VPs) can be defined as electronic representations of realistic clinical cases. They have been proposed as being an ideal tool to teach clinical reasoning skills. A recent literature review, and systematic review of the literature has highlighted a lack of evidence supporting individual design properties for virtual patients as in other elearning areas. VPs are widely used in up to one third of US and Canadian medical schools, however until 2007 development costs were high.[...] This facilitates collaboration, research, open access and the upkeep of these electronic resources. This has potentially changed the working definition of what a VP is, by re-defining properties and dimensions of VPs. A European Commission funded study has produced self-reported evaluation scores to help evaluation, the EViP project. There are numerous VP design properties identified in the literature and their impact on the learning experience are poorly understood. Of particular interest are, firstly, the use of branching case pathways , and secondly, the role of structured feedback to promote clinical reasoning. Branching cases are more difficult to construct, more expensive when compared with linear cases, and may have unpredictable effects on individual students.	Bateman J, Allen ME, Kidd J, Parsons N, Davies D. Virtual patients design and its effect on clinical reasoning and student experience: a protocol for a randomised factorial multi-centre study. BMC Med Educ. 2012;12:62.
Over the last decade, use of virtual patient technology- from computer-based virtual reality programs to full-size lifelike simulators- emerged as a new method of training health care providers in clinical and communication skills. In pharmacy education, the virtual patient is a simulated patient, typically generated by a computer software program, and used to simulate realistic clinical scenarios. Teaching methods using virtual patient technology in health care education allow students to adopt the role of a health care provider in a safe environment where they can develop clinical and communication skills, such as patient assessment, interview skills, and information provision, without compromising the welfare of an actual patient. This can be achieved through the use of a range of virtual clinical scenarios applied to individual case-based assignments. Typically, students interact with a virtual patient, and during the process of assessment, propose some health care intervention, which is then recorded to complete the case. As computer-generated virtual patients are available on demand, students are able to practice their clinical and communication skills at any time. An advantage of using virtual patients in the teaching of medication counseling compared to traditional teaching methods is the ability of the virtual patient to emulate the psychological state of the different types of patients that pharmacists encounter in the practice of pharmacy (eg, angry, anxious, ambivalent, passive, assertive, and persuasive). [...]Virtual patient technology also can be used to assess student skills. [...] The standardized nature of virtual patient simulations also increases the validity of	Jabbur-Lopes MO, Mesquita AR, Silva LM, De Almeida Neto A, Lyra DP Jr. Virtual patients in pharmacy education. Am J Pharm Educ 2012; 76(5):92

assessments, as each scenario is relatively consistent. The use of virtual patient technology allows students to develop their competencies (knowledge, skills, and attitudes) in providing care to patients.	
standardized virtual patients (i.e., actors whose performances were recorded onto a CD-ROM that was played for physician-participants)	Hooper LM, Epstein SA, Weinfurt KP, DeCoster J, Qu L, Hannah NJ. Predictors of primary care physicians' self-reported intention to conduct suicide risk assessments. J Behav Health Serv Res. 2012;39(2):103-15.
Over recent years, an increasing number of computer-based simulations of patients have been proposed for both training and assessment in medical education, often referred to as "virtual patients" (VP). Although the use of VP has not yet entered the main stream of medical education and is only currently starting to be integrated into the regular curriculum in few universities, there is no doubt in the potential of VPs to fill significant gaps in the current tuition of medical students. VPs are excellent teaching tools for developing clinical reasoning and decision-making skills and improving clinical competency. [..] VPs are ideally suited to this task, as potential variations in VP design are practically limitless. VPs can incorporate images, sound, videos, lab tests and imaging results, both for early medical students, and for later years' courses. Although VPs cannot replace interpersonal communication with real patients, students tend to react similarly to real and simulated patients. Web-based VP software has the most important advantage of providing an interactive "game", enabling practicing patient management in a fast sequence whenever and wherever convenient for the student, providing real-time and accurate feed-back. The same system can be used for exams, providing statistically analyzed results within seconds after the exam. The most advanced VP programs replaced the multiple choice system by natural language processing, designed to enable the student to ask any medical related question via an interactive dialogue with text entry.	Oliven A, Nave R, Gilad D, Barch A. Implementation of a web-based interactive virtual patient case simulation as a training and assessment tool for medical students. Stud Health Technol Inform 2011;169:233-7.
Simulated patients are realistic objects constructed using a series of parameters that define their verbal, emotional and motor responses....	Peñaloza-Salazar C, Gutierrez-Maldonado J, Ferrer-Garcia M, Garcia-Palacios A, Andres-Pueyo A, Aguilar-Alonso A. Simulated interviews 3.0: virtual humans to train abilities of diagnosis-usability assessment. Stud Health Technol Inform 2011; 167:165-9.
One notable uptake of instructional multimedia in medical education has been around the use of 'virtual patients'. Computer-based educational simulations of patient encounters were first developed in the 1960s and were revisited as personal computers and disc-based media became available, and again in the context of the Web and more virtual worlds. Although there has been much work in this area there are a few common designs such as history examination- investigation-diagnosis-therapy (HEIDR for short), scenario-based decision-making and interactive question and response. Almost all of these are rendered using hyperlinks, dropdown lists, checkboxes, radio buttons, typein- text boxes and drag and drop items. The use of virtual worlds has added avatars with 3-	Ellaway RH, Davies D. Design for learning: deconstructing virtual patient activities. Med Teach. 2011;33(4):303-10

dimensional movement and audio/text communication. [...] We propose a theoretical construct to unite the design of virtual patients, their use, and their relation to other educational interventions such as simulation, problem-based learning and assessment. [...] A virtual patient can be realized using a wide range of presentations, styles and configurations that make it difficult to define what exactly a virtual patient is. The lack of common and comprehensive models is one of the barriers to quality research into the design and use of virtual patients	
The increasing availability and power of computing has led to growing interest in computer simulations for use in examinations, creating assessment virtual patients (AVPs). They can potentially test knowledge and data interpretation, incorporate images, sound or video and test decision making. Such AVPs could represent the most comprehensive, integrated assessment possible that is both objective and feasible. [...] Assessment Virtual Patients (AVPs) are computer based simulations of patient management designed to predict performance in clinical settings. [...]AVPs allow candidates to choose between different parts of history, examination, investigations or treatments. The clinical condition of the patient can depend on choices made. [..]AVPs offer many advantages over current assessment tools, offering integrated testing of knowledge, data interpretation and management skills set in a more realistic context than paper-based assessments. [...] They can also incorporate use of resources, including time or money. AVPs can be used synchronously or asynchronously, on or off line and can either produce marks visible or invisible to the candidate. They can be used in formative or summative assessments. AVPs allow examiners to test knowledge, interpretation and decision making, all in the context of a patient scenario. Sound, images and video can be incorporated to test a candidate's skill in integrating multiple pieces of information into a presentation. Being multi-step, the patient can improve, deteriorate or develop new features. Because each candidate has the same 'patient', an AVP should be reliable. AVPs have excellent face validity and also would be expected to have better predictive validity than other formats, as no other test better integrates different patient management skills. AVP designs can incorporate consequences, based on choices made by the candidate. The exam can mirror real life, where a patient can be treated successfully in several different ways, and mistakes are often correctable.	Round J, Conradi E, Poulton T. Improving assessment with virtual patients. Med Teach. 2009;31(8):759-63.
VPs are defined as interactive computer programs that simulate real-life clinical scenarios in which the learner acts as a healthcare professional obtaining a history and physical examination and making diagnostic and therapeutic decisions. VPs offer several potential advantages over SPs such as limiting the effort and expense associated with SP training, creating diverse virtual clinical scenarios that are difficult to duplicate with SPs, and providing a controllable, secure, and safe learning environment with the opportunity for extensive repetitive practice. VP scenarios have the potential to accelerate student learning and enhance traditional SP teaching and testing programs [..] Preliminary studies reveal that most students would use the virtual teaching tool in their preparation for interaction with SPs and real patients	Deladisma AM, Cohen M, Stevens A, Wagner P, Lok B, Bernard T, Oxendine C, Schumacher L, Johnsen K, Dickerson R, Raij A, Wells R, Duerson M, Harper JG, Lind DS; Association for Surgical Education. Do medical students respond empathetically to a virtual patient? Am J Surg 2007;193(6):756-60.
is the virtual patient – a knowledge-based model and simulation of a person suffering from one or more diseases. The virtual patient is a "double agent" in that it models and simulates both the physiological and the cognitive functionality of a human. Users can interview a virtual patient; order lab tests; receive the results of lab tests from	Nirenburg S, McShane M, Beale S, Jarrell B, Fantry G. Integrating cognitive simulation into the Maryland virtual patient. Stud Health

technician agents; receive interpretations of lab tests from consulting physician agents; posit hypotheses, clinical diagnoses and definitive diagnoses; prescribe treatments; follow-up after those treatments to judge their efficacy; follow a patient's condition over an extended period of time, with the trainee having control over the speed of simulation (i.e., the clock); and, if desired, receive mentoring from the automatic mentor.	Technol Inform 2009;142:224-9.
Studies have shown that there is a potential for effective computer-assisted learning (CAL) in undergraduate clinical dental programmes and that it can be used as an adjunct to traditional education or as a means of self-instruction. The advantages of computer-assisted learning are seen for example in self-paced and self-directed learning and increased motivation. The use of CAL in dentistry dates back to the 1980s and various applications aimed at presenting patient cases and/or demonstrating certain practical tasks, have been developed since then. One example of this is virtual patient (VP) systems, which are interactive computer programs that simulate real-life clinical scenarios in which the student acts as a health care professional. In these scenarios, the student obtains a history, performs physical examinations, orders and interprets lab and/or imaging tests and finally makes diagnostic and therapeutic decisions.	Zary N, Johnson G, Fors U. Web-based virtual patients in dentistry: factors influencing the use of cases in the Web-SP system. Eur J Dent Educ 2009;13(1):2-9.
Virtual Patients are computer-based simulations of a clinical encounter where the user plays the role of a healthcare provider while receiving in-context instruction. This unique pedagogical approach enables active case-based learning for learners. Academic institutions around the world have developed high-quality virtual patients using many different authoring and playback technologies. However, sustainability and scalability have proved challenging due to the number of cases needed and production costs. [...] Computer-based Virtual Patients (VP) are now being explored as a new method for creating high-fidelity simulated patient interactions. The term "Virtual Patients" is used to represent a variety of developing technologies to create interactive computer models of the patient-provider interaction that simulates communication and information gathering, and allows the user to apply diagnostic reasoning. Since computer-based VPs can be used at any time, they can be integrated into curricula in a much more flexible manner. Since they are computer-based, they can also be deployed at the point of care, and many learners can use a single VP case simultaneously. They can be developed at one institution and immediately and widely externally accessible, providing training to regions that do not have the resources to develop them, or have a sudden surge in need for specialized training, such as a disaster or crisis. VPs offer true standardization across interactions creating a more consistent experience for learners. The VP has the advantage of being easily modified to demonstrate a variety of clinical or interview scenarios, for example changing the gender or race of the patient (6). Virtual Patients have worked well to simulate concrete and predictable topics such as physiological processes and performing procedures. More recently they have been used to model the patient interview and are being used to teach clinical interviewing skills. [...]Previous work has included the development of virtual patient cases designed to teach bedside competencies of bioethics, basic patient communication and history taking, and clinical decision-making [...] Though they are more scalable and reusable than traditional live cases, the process of creating quality VP cases is both expensive and time consuming. [...]The VP cases tended to focus on primary care disciplines and had limited racial or ethnic diversity. (12) The resource intensive nature of	Triola MM, Campion N, McGee JB, Albright S, Greene P, Smothers V, Ellaway R. An XML standard for virtual patients: exchanging case-based simulations in medical education. AMIA Annu Symp Proc. 2007;741-5.

this approach has led many faculty to believe that the most efficient use of these cases is for different institutions to exchange, edit and reuse them, creating a pool of cases for general use.	
Virtual Patients are computer simulations designed to train or assess clinicians in information gathering, diagnostic reasoning and management of individual patients. The majority of online Virtual Patient designs have focused on a single user or group interaction with a single patient.	Radon K, Carvalho D, Calvo MJ, Struempell S, Herrera V, Wengenroth L, Kausel G, Marchetti N, Rojas DS, Russ P, Hege I. Implementation of virtual patients in the training for occupational health in Latin America. Int J Occup Environ Health. 2011;17(1):63-70.
The development and use of virtual patients has become more expansive.[...] Virtual Patients may be defined as a simulation through which a user has to undertake communication, information gathering and apply diagnostic reasoning towards a computer generated patient. [...] there is an increasing variety of interfaces for presenting virtual patient cases, and in particular an interest in the possibility of using 3 dimensional virtual worlds as the primary platform on which to host them	Patel V, Aggarwal R, Taylor D, Darzi A. Implementation of virtual online patient simulation. Stud Health Technol Inform. 2011;163:440-6.
Virtual patients (VPs) are virtual interactive agents who are trained to simulate a particular clinical presentation of a patient with a high degree of consistency and realism. VPs have commonly been used to teach bedside competencies of bioethics, basic patient communication and history taking, and clinical decision making. Virtual patients can provide valid, reliable, and applicable representations of live patients	Parsons TD, Kenny P, Ntuen CA, Pataki CS, Pato MT, Rizzo AA, St-George C, Sugar J. Objective structured clinical interview training using a virtual human patient. Stud Health Technol Inform 2008;132:357-62.
Virtual Patients are an essential component of medical virtual reality. [...] Like in many other areas of Virtual Reality, the development of virtual humans for medical applications is becoming an important research topic Virtual Patients can be part of realistic simulations which include visual, interactive and knowledge-based aspects. [...] The key idea in developing a complete virtual patient is to be able to integrate various physiological sub-systems that could support the modelling of a wide range of pathological situations . [...] Virtual patients in clinical medicine can be conceived of as visual interfaces to knowledge- based systems, simulating clinical situations from first principles. They offer the potential to embody the medical knowledge in a realistic context, supporting many forms of training and simulation.	Simo A, Cavazza M, Kijima R. Virtual patients in clinical medicine. Stud Health Technol Inform. 2004;98:353-9.
The use of a virtual patients program aimed to develop an authentic assessment method to improve student's learning experience and develop critical thinking skills. The virtual patient program is a computer program that provides access to a list of "virtual patients" to which a range of clinical scenarios can be applied to develop individual case-based assignments. The assessment uses these clinical cases, a criterion-referenced marking scale and provides both summative and formative assessment by peers and tutors of an oral case presentation.	Marriott JL. Use and evaluation of "virtual" patients for assessment of clinical pharmacy undergraduates. Pharmacy Education: An International Journal of Pharmaceutical Education 2007;7(4):341-349.
The term Virtual Patient is an umbrella term for a type of interactive medical system used for teaching and learning medicine, especially clinical reasoning. According to the electronic Virtual Patients (eViP) project website a Virtual Patient can be formally defined as "an interactive computer simulation of real-life clinical scenarios for the purpose of medical training, education or assessment. These systems exist in many forms, and	Bloice M., Simonic K.-M., Kreuzthaler M., Holzinger A. Development of an interactive application for learning medical procedures and clinical decision making. Lecture Notes in

range from physical robotic patients, entire hospital simulation systems, to online accessible interactive patient cases.	Computer Science 7058 LNCS:211-24
Virtual Patients have proven to be an effective educational tool for learning and applying clinical examination skills. Interactive virtual patient scenarios provide opportunities for medical students to practice and improve verbal and non-verbal communication through the use of performance feedback. [...] Evidence supports that interactive VPs fill a niche in medical education and testing for scenarios that cannot be practiced outside the virtual environment or with standardized patients. Not only are VPs effective in medical curriculum, as evidence by various studies, they are applicable in understanding theways in which learning occurs and can be implemented into a number of educationl settings. [...] We define virtual patients as embodied conversational agents-computer-generated virtual characters that are controlled by a computer algorithm with whom users can conduct a conversation. This cobversation is modeled as a knowledge-base of possible answers the VP can give to input from the user. Input from the user can be in the form of questions or gestures. Users may type, speak or choose from a list their questions to the VP.	Ferdig R.E., Schottke K., Rivera-Gutierrez D., Lok B. Assessing past, present, and future interactions with virtual patients. International Journal of Gaming and Computer-Mediated Simulations 4(3):20-37
These virtual patients are realistic objects that can in real-time with the user using a series of parameters that define their verbal, emotional and motor responses. From them the trainee must obtain the data needed to make an accurate diagnosis. The high level of flexibility and interactivity increases the trainees' sensation of participating in the simulated situation, leading an improving of the learning of the skills required. [...] When using AI healthcare students can engage in a natural interaction with a virtual patient (that should display a wide range of moods), understanding more deeply their beliefs, motives and emotions, and providing a realistic setting in which she can learn specific skills such as those involved in the diagnostic interview. This should facilitate that this learning can be generalized more easily to the real world situations.	Gutierrez-Maldonado J, Alsina-Jurnet I, Rangel-Gomez MV, Aguilar-Alonso A, Jarne-Esparcia AJ, Andres-Pueyo A, Talarn-Caparros A. Virtual intelligent agents to train abilities of diagnosis in psychology and psychiatry, Studies in Computational Intelligence 2008;142:497-505
Extending virtual humans to health care has been accomplished with virtual patients (VP). VPs are virtual interactive agents designed to simulate a particular clinical presentation of a patient with a high degree of consistency and realism. VPs have recently been implemented to teach bedside competencies of bioethics, basic patient communication and history taking, clinical decision making, and psychotherapy training. These VPs have been found to provide valid, reliable, and applicable representations of live patients; and can augment many live actor simulated patient programs. VPs could also be used to teach caregivers or family members how to communicate or interact with their aging loved ones or relatives with mental health issues.	Kenny P, Parsons T, Gratch J, Rizzo A. Virtual humans for assisted health care. 1st International Conference on Pervasive Technologies Related to Assistive Environments, PETRA 2008
VPs are virtual interactive agents who are trained to simulate a patient's particular clinical presentation with a high degree of consistency and realism. VPs have commonly been used to teach bedside competencies in bioethical decision making, basic patient communication and history taking, and clinical decision making. VPs can provide valid, reliable, and applicable representations of live patients	Kenny P, Parsons TD, Gratch J, Leuski A, Rizzo AA. Virtual patients for clinical therapist skills training. Lecture Notes in Computer Science 2007;4722:197-210
Recent research has established the potential for virtual characters to act as virtual standardized patients VP for the assessment and training of novice clinicians.[...] The potential of using virtual humans as virtual standardized patients (VP) for use in clinical assessments, interviewing and diagnosis training is becoming recognized as the	Kenny P, Parsons TD, Gratch J, Rizzo AA. Evaluation of Justina: A virtual patient with PTSD. Lecture Notes in Computer Science

technology advances. These VPs are embodied interactive agents who are designed to simulate a particular clinical presentation of a patient with a high degree of consistency and realism. VPs have commonly been used to teach bedside competencies of bioethics, basic patient communication, interactive conversations, history taking, and clinical decision making. VPs can provide valid, reliable, and applicable representations of live patients. [...] Since virtual humans and virtual environments can allow for precise presentation and control of dynamic perceptual stimuli (visual, auditory, olfactory, gustatory, ambulatory, and haptic conditions), conversations and interactions, they can provide ecologically valid assessments that combine the control and rigor of laboratory measures with a verisimilitude that reflects real life situations	2008;5208:394-408
A patient is a person suffering from an illness, that has been determined or not. Most of the time this term is used to designate a human being. When a patient is placed at the disposal of a physician or when he receives a medical attention, he becomes then a patient. An interactive virtual patient is therefore a computer program that simulates the real life of a patient's clinical scripts as define above. It has as objective to provide an efficient means to the health professionals' daily practice (to diagnose the illnesses in a suitable virtual environment).	Monthe V., Batchakui B., Tangha C. MV-SYDIME: A virtual patient for medical diagnosis apprenticeship. International Journal of Online Engineering 2012;8(4):21-31
VPs are ontological objects, specifically, subclasses of VIRTUAL-HUMAN that have various diseases and disorders. Like all VIRTUAL- HUMANs, their large inventory of property-value pairs changes in response to ontological events, including internal and external stimuli. All VPs inherit the lion's share of physiology from VIRTUAL -HUMAN , meaning that GERD -PATIENT and HEART -DISEASE -PATIENT (as ontological concepts, not instances) differ only with respect to the disease-specific changes that affect certain of their property values over time.	Nirenburg S, McShane M, Beale S, O'Hara T, Jarrell B, Fantry G, Raczek J. Cognitive simulation in Virtual Patients. FLAIRS 2006 - Proceedings of the Nineteenth International Florida Artificial Intelligence Research Society Conference 2006:174-75
The term Virtual Patient (VP) has been coined to describe interactive computer interfaces or virtual humans used in health care education. VPs allow the learner to take the role of health care professional and develop clinical skills such as making diagnosis and therapeutic decisions	Sakpal R. Virtual patients to teach cultural competency. ICMI'12 - Proceedings of the ACM International Conference on Multimodal Interaction 2012:349-52
A related difficulty, which derives from the "dialog" model used in many systems, is to decide which amount of information should be introduced to the trainee at each step, and how to give a more active role to the trainee. These problems could be addressed by providing a realistic visualization of the clinical situation using a virtual patient in a virtual hospital context. Virtual patients in clinical medicine can be conceived of as visual interfaces to knowledge-based systems, simulating clinical situations from first principles. They offer the potential to embody the medical knowledge in a realistic context, supporting many forms of training and simulation.	Simo A, Cavazza M. Medical education interfaces through virtual patients based on qualitative simulation. Studies in Computational Intelligence 2007;48:255-90
One cost-effective method of teaching procedural knowledge is the use of virtual patients. In the context of medical education, this term [VP] generally refers to any software that allows case-based training. Cases are largely text-based, sometimes with additional audio and video material that shows real patients. Learning is not team-based and the environment is not immersive.	Von Zadow U, Buron S, Harms T, Behringer F, Sostmann K, Dachselt R. SimMed: Combining simulation and interactive tabletops for medical education. Conference on Human Factors in Computing Systems - Proceedings 2013;1469-78

The process of creating and designing Virtual Patients for teaching students of medicine is an expensive and time-consuming task [...] Much research has shown that Virtual Patients are a credible and effective form of teaching, and they have been shown to improve knowledge retention, student participation, and other factors. However, they are also expensive and time consuming to produce, resulting in lower adoption rates than might be expected [...] According to the European Commission co-funded Electronic Virtual Patients (eViP) project, a Virtual Patient is "an interactive computer simulation of real-life clinical scenarios for the purpose of medical training, education or assessment". This definition covers all electronic Virtual Patients, however for the purposes of this review this definition has been expanded to include other forms of virtual patient, including hardware simulators, mannequins, and videos.	Bloice MD, Simonic KM, Holzinger A. On the usage of health records for the design of virtual patients: a systematic review. BMC Med Inform Decis Mak 2013; 13(1):103.
Advances in technology have made it possible to provide faculty members and students with unique opportunities to use human patient simulators, standardized patients, and virtual patients in an effort to bridge the gap between the classroom and clinical practice [...] Virtual patients are computer-based simulations designed to complement clinical training. [..] Virtual patient software technology offers the distinct advantage of providing more versatility, mobility, and accessibility through a virtual environment that maximizes the realism of actual patient care at a low level of risk, avoiding the need for expanded physical space requirements and the associated overhead and staffing. These simulation scenarios can increase students' exposure to patients living in a range of environments from rural communities to urban locations.12 Demographic variables and comorbidities can easily be modified in a virtual case to encompass a more broad and diverse patient population compared to other forms of simulation. Unlike traditional methods of knowledge assessment, virtual patient simulation encourages students to move beyond rote memorization and toward clinical decision-making using evidence-based practice.14 While a short-answer or multiple-choice examination presents a patient at only 1 specific point in time, students can follow a virtual patient's medical progress over time with the use of a simulated electronic medical record (EMR). Virtual patient platforms can also provide real time clinical guidance as students move through cases, as opposed to delayed feedback on written examination performance because of lengthy grading processes	Douglass MA, Casale JP, Skirvin JA, Divall MV. A virtual patient software program to improve pharmacy student learning in a comprehensive disease management course. Am J Pharm Educ. 2013; 77(8):172.
E-learning resources, such as virtual patients (VPs), can be more effective when they are integrated in the curriculum [...] Contrary to earlier assumptions that e-learning resources, including virtual patients (VPs), would automatically reach their full potential when students were exposed to them, recent studies have reported low acceptance, usage and satisfaction among students when e-learning resources are poorly integrated with, or offered as an add-on to, other curricular components (Haag et al. 2007; Fischer et al. 2008). Research has also shown that VPs are particularly suited to foster clinical reasoning, and their use is on the increase in medical education As part of our search for ways of optimizing the power of VPs to enhance medical education, the development of clinical reasoning skills in particular, we designed and studied integration scenarios for VPs, defined as ‘‘interactive computer simulation[s] of real-life clinical scenarios for the purpose of medical training, education, or assessment’’.	Huwendiek S, Duncker C, Reichert F, De Leng BA, Dolmans D, van der Vleuten CP, Haag M, Hoffmann GF, Tönshoff B. Learner preferences regarding integrating, sequencing and aligning virtual patients with other activities in the undergraduate medical curriculum: A focus group study. Med Teach 2013;35(11):920-9.
Virtual Patients (VPs) have been used in undergraduate healthcare education for many years [...] Virtual Patients	Ekblad S, Mollica RF, Fors U, Pantziaras I,

(VPs) are interactive computer simulations of patient encounters used in health care education. The VP is a virtual representation of a patient encounter for learning and assessment, typically including interactive features for illness history taking, physical examination, laboratory tests as well as features for suggesting diagnosis and treatment plan. [..] Authenticity is critical to whether a virtual patient can be considered to be part of a situated learning endeavor, indicating that VPs may provide reliable, valid, and applicable representations of live patients. VPs can emulate a problem-based learning environment to assist medical practitioners in active, independent (and group) learning and problemsolving and are also giving the learner automatic feedback on the patient management process. VPs have been shown to have a great educational value especially for training clinical reasoning. Virtual patients are also shown to have a potential to emphasize socio-cultural aspects and cultural differences as they pertain to healthcare education. The use of VPs has been proven to be effective in the training of surgeons, medical students as well as psychology students. [..] VPs may offer a holistic approach to medical education using a model that emphasizes both the patient and the doctor' s perspective. Therefore VPs might have a potential to dramatically increase skills and knowledge as well as foster trust, respect and empathy in the doctorpatient relationship.	Lavelle J. Educational potential of a virtual patient system for caring for traumatized patients in primary care. BMC Med Educ. 2013;13:110.
Virtual patient (VP) simulation is a computer-based learning method aimed at developing clinical reasoning and decision making skills. Considering the exponential growth in the number of medical students and the limited access to patients, the necessity to structure and standardize medical training has also risen in resource-limited settings. VP technology is increasingly used to train healthcare personnel or surgeons, as simulations with VP have demonstrated the potential to develop the acquisition of knowledge, skills and clinical reasoning. [...] However, there is little evidence on the ability of VP systems, based on medical consultations (training of clinical reasoning), to develop real-life operational clinical skills. [...]	Bediang G, Franck C, Raetzo MA, Doell J, Ba M, Kamga Y, Baroz F, Geissbuhler A. Developing clinical skills using a virtual patient simulator in a resource-limited setting. Stud Health Technol Inform. 2013;192:102-6.
One increasingly used but understudied e-learning approach is the virtual patient (VP).VPs are interactive computer programs, based on real clinical cases, that have the benefit of being engaging, emphasizing decision making, and allowing for presentation of clinical situations not commonly encountered in real life.	Harris JM Jr, Sun H. A randomized trial of two e-learning strategies for teaching substance abuse management skills to physicians. Acad Med. 2013;88(9):1357-62.
"Virtual Patients" (clinical case simulations to improve and assess professional skills)	Trichkova E, Kossekova G. Interactive biochemistry - Sofia (IBS) - A flexible Web-based e-learning system. Proceedings of the IADIS International Conference e-Learning 2011, Part of the IADIS Multi Conference on Computer Science and Information Systems 2011, MCCSIS 2011;1:3-10
Virtual patients are typically animated NPCs [non-player character] with which the user, who assumes the role of a healthcare provider, interacts. An essential feature of any virtual patient is the interactive interface that enables the user to query the the patient and receive a patient response that is supplied by the computer. Characteristics that	LeFlore JL, Anderson M, Zielke MA, Nelson KA, Thomas PE, Hardee G, John LD. Can a virtual patient trainer teach student nurses how

are shared among games, virtual worlds, and virtual patients include that they are 3D and immersive, incorporate virtual reality by recreating real environments, and are used to make abstract concepts more understandable.1 They all have visual and auditory feedbac and a graphic interface. [..] Research regarding games, virtual patients, and virtual worlds in healthcare education has demonstrated their efficacy in teaching didactic information to promote knowledge acquisition, critical thinking/reasoning, and skilled communication.	to save lives-teaching nursing students about pediatric respiratory diseases. Simul Healthc. 2012;7(1):10-7.
The VP allows the conceptualization of the accident history, where patient-specific problems can be simulated directly after the accident, including patient-related issues like medical history taking, physical examination and remaining patient symptoms after the initial treatment. [...] VP models have proven to engage and motivate learners.	Courteille O, Ho J, Fahlstedt M, Fors U, Felländer-Tsai L, Hedman L, Möller H. Face validity of VIS-Ed: a visualization program for teaching medical students and residents the biomechanics of cervical spine trauma. Stud Health Technol Inform. 2013; 184:96-102.
Virtual patients have been found to promote critical thinking and decision-making skills in medical students. If used in the right way it requires active participation by the students, which is associated with improved learning outcomes compared with no intervention. As the clinical encounter lies in the centre of medical training it could additionally be the focus of a student-centred e-learning approach, where students can experience an interactive multimedia environment as well as formative assessment.	Abendroth M, Harendza S, Riemer M. Clinical decision making: a pilot e-learning study. Clin Teach 2013;10(1):51-5.
VP focuses on going through a complete process of clinical reasoning until the virtual patient is cured or dies. [...] Content validity and reliability are low due to the low degree of representativeness of the test compared with the subject matter; a considerable amount of time is needed to complete one VP. VPs are very expensive to develop. Special software and hardware is needed to implement VPs in education. No data were found in literature on the time students need to answer a VP..	van Bruggen L, Manrique-van Woudenbergh M, Spierenburg E, Vos J. Preferred question types for computer-based assessment of clinical reasoning: a literature study. Perspect Med Educ. 2012;1(4):162-71.
Virtual patients (VPs) have a very specific purpose: to provide training for novice health care practitioners without risk to the patient. VPs also allow for delivery of a consistent set of patient cases to the novice-in-training, some-thing a clinical rotation cannot do. For the most common type of VP, a user progressively gathers information about a specific patient by clicking on a series of linked information pages before making a final decision about the patient's diagnosis and treatment. This type of system has value in that it can introduce preclinical health care students to the process of history and examination data-gathering and give them familiarity with the decision mak-ing process. Unfortunately, with this linked-page style of VP, student users do not formulate their own questions, which is an essential skill that they must acquire. At most, it forces the student user to choose pre-scripted questions and remember the resulting information before he or she makes choices. Another type of VP system is one in which student users (by speech or text) submit their own questions and the VP recognizes and responds with an appropriate answer.	Clark GT, Suri A, Enciso R. Autonomous virtual patients in dentistry: system accuracy and expert versus novice comparison. J Dent Educ. 2012;76(10):1365-70.
Simulation in medical education provides students with opportunities to practice interviews, examinations, and diagnosis formulation related to complex conditions without risks to patients [...] Virtual patients (VPs) presented through computer-based medium are particularly of interest due to their capacity to display a variety of complex	Johnson TR, Lyons R, Chuah JH, Kopper R, Lok BC, Cendan JC. Optimal learning in a virtual patient simulation of cranial nerve

pathologies and clinical scenarios in a cost-effective and easily distributable format.	palsies: the interaction between social learning context and student aptitude. Med Teach 2013;35(1):e876-84.
A virtual patient provides an engaging and interactive experience in which the learner is the principal decision maker rather than a passive observer.	Subramanian A, Timberlake M, Mittakanti H, Lara M, Brandt ML. Novel educational approach for medical students: improved retention rates using interactive medical software compared with traditional lecture-based format. J Surg Educ. 2012;69(4):449-52.
Virtual patient designs are typically linear, branching or 'semi-linear'. A linear design can simulate history taking and test the knowledge required for physical examination, but is inappropriate for simulating clinical decision making. Branching designs simulate clinical decision making, but are not suitable for practising history taking and physical examination because they provide options that act as clues for students.	Pinnock R, Spence F, Chung A, Booth R. evPaeds: undergraduate clinical reasoning. Clin Teach. 2012;9(3):152-7.
Computer-based simulations of patient cases have long been used for learning and training in healthcare education. These learning tools are frequently referred to as Virtual Patients (VPs), but have also been labeled Interactive Simulated patients, Simulated Cases or Avatars. VPs may be used throughout the medical school curriculum including pre-clinical courses. VPs are commonly recommended for teaching clinical reasoning and clinical decision making, but have also been used for teaching basic communication skills with patients (learning how to ask about the patient's illness history) and to broach the subject of bioethics. Furthermore, there are also indications for using VPs to emphasize socio-cultural aspects and cultural differences as they pertain to the discipline of the practice of medicine. Moreover, VPs have been used in both traditional curricula and problembased learning (PBL)-based programs. [...] Today, VPs have been accepted as viable supplements for the delivery of educational resources with good learning outcomes. The use of VPs for assessment is a more recent application and has been described as a possible way to assess clinical reasoning and/or clinical decision-making abilities among students.	Gunning WT, Fors UG. Virtual patients for assessment of medical student ability to integrate clinical and laboratory data to develop differential diagnoses: comparison of results of exams with/without time constraints. Med Teach. 2012;34(4):e222-8.
There is a long tradition of the use of patient cases for teaching in medical education. Computerised cases have allowed new dimensions to emerge in the form of virtual patients (VPs).[...] The use of VPs has been introduced mainly as an educational activity to promote clinical reasoning. Typical VP use involves asking the VP questions regarding symptoms, followed by conducting a physical examination and laboratory tests when appropriate. Lastly, a diagnosis and differential diagnoses are presented. Students' use of VPs has demonstrated effects on clinical reasoning, knowledge and other skills. However, these effects do not supersede those arising from instructional methods that are not computerbased and we have yet to attain a full understanding of how to structure the use of VPs in order to optimise student benefit [...] In the clinical environment the use of VPs has been described as intermediating between factual knowledge and authentic patient experience. However, merely	Johnson TR, Lyons R, Chuah JH, Kopper R, Lok BC, Cendan JC. Optimal learning in a virtual patient simulation of cranial nerve palsies: the interaction between social learning context and student aptitude. Med Teach 2013;35(1):e876-84.

introducing VPs into a course does not fulfil this purpose. The focus of investigation should be shifted from VPs per se to their educational function in relation to how the overall learning activity is framed. This requires a better understanding of principles for the integration of VPs into a course.	
The variety of VP programs is vast; many are used to practice medical procedures, fewer for practicing interviewing and diagnostic skills. VP technology ranges from showing images and texts on a computer screen to having a patient-image projected on a screen , with some programs accepting oral questions from the learner, recognizing key words in the question, and then producing a verbal answer. Also, some programs use video and audio clips, including heart sounds, MRIs, and X-rays. Depending on the VP program, time-lines and patient story-development can be incorporated by unlocking additional questions once the critical question has been asked. These sophisticated programs typically start at $15,000, with costs quickly rising with program complexity. VP programs offer multiple advantages: they can provide formative or summative feedback and assess clinical reasoning and decision-making skills. Individual resident performance can be assessed and compared over time, and with peers, in a highly standardized manner not possible with actual patients or live standardized patients (SPs). Virtual programs have the advantage of consistency when compared with objective structured clinical exams. VPs allow the resident to practice—and programs to evaluate— competence in recognizing a variety of diagnoses. The ability to incorporate sociocultural, ethical, and religious issues that can influence diagnosis and treatment outcomes further enhances the learning process. Virtual encounters are also psychologically safer for the novice interviewer	Williams K, Wryobeck J, Edinger W, McGrady A, Fors U, Zary N. Assessment of competencies by use of virtual patient technology. Acad Psychiatry 2011; 35(5):328-30.
Computer-based simulations of real-life clinical scenarios are are increasingly being used in medical education. [..] Such simulations or virtual patients (VPs) provide learners with an interactive opportunity to engage in clinical problem solving and receive feedback on their performance. [...]VPs utilise a multimedia experience to provide a 'more realistic' context to learning. [...] Despite the increasing popularity of VPs, it is recognised that such learning packages are resource intensive and expensive to produce.	Gormley GJ, McGlade K, Thomson C, McGill M, Sun J. A virtual surgery in general practice: evaluation of a novel undergraduate virtual patient learning package. Med Teach. 2011; 33(10):e522-7.
Medical training cases (virtual patients) are in widespread use for student education.	Hörnlein A, Mandel A, Ifland M, Lüneberg E, Deckert J, Puppe F. Acceptance of medical training cases as supplement to lectures. GMS Z Med Ausbild 2011;28(3):Doc42
[...]a specific type of computer- assisted instruction – virtual patients – in medical education [...] Developing a virtual patient case program at a single institution to comprehensively cover a curriculum is virtually impossible due to the scope and level of resources needed for such a project (Fall et al. 2005). Implementing or integrating virtual patients into the clerkship curriculum has thus been beyond the reach of most clerkship or course directors. [...] To achieve the most value from a virtual patient program, the cases must be thoughtfully integrated in the clerkship experience (Haag et al. 2007), a process significantly facilitated by the comprehensive coverage of the national curriculum	Berman NB, Fall LH, Chessman AW, Dell MR, Lang VJ, Leong SL, Nixon LJ, Smith S. A collaborative model for developing and maintaining virtual patients for medical education. Med Teach. 2011;33(4):319-24.

Virtual patients, also known as computer-based simulations, have been proposed as alternatives to standardized patients and supplements to the traditional medical school curriculum. Like standardized patients, virtual patients allow learners to interact with simulated patients in controlled learning environments and to receive feedback from virtual faculty preceptors. Virtual patients also allow medical educators to supplement or fill gaps in preexisting curricula by exposing students to clinical experiences they may not otherwise encounter. [...] In a review of the medical literature on virtual patients, Cook and Triola4 found that development of clinical reasoning skills, a process that matures through deliberate practice with multiple and varied clinical scenarios, is a major strength of the virtual patient method.	Ricciotti HA, Hacker MR, De Flesco LD, Dodge LE, Huang GC. Randomized, controlled trial of a normal pregnancy virtual patient to teach medical students counseling skills. J Reprod Med 2010; 55(11-12):498-502.
[...] Virtual Patients (VPs) have successfully been applied in medical and healthcare education for a number of years (Ellaway et al., 2008). Most VP systems have common features, including taking a medical history, physical examination, lab/imaging tests, and features for suggesting appropriate diagnosis and treatment. Most systems also provide feedback concerning interactions between the healthcare practitioner and the VP. The use of VPs has been suggested not only for educational purposes, but also in the assessment of CR. VP systems are in fact focused on CR, and allow for the possibility to track in detail every interaction between the user (i.e. student) and the Virtual Patient for CR assessment purposes.	Forsberg E, Georg C, Ziegert K, Fors U. Virtual patients for assessment of clinical reasoning in nursing - a pilot study. Nurse Educ Today. 2011; 31(8):757-62.
It is little surprise that there has been a corresponding growth in the use of so-called 'virtual' patients in medical and health care education. A virtual patient can take many forms: computer simulations of biochemical processes, physical simulators such as manikins, data sets representing actual patients (so that, in effect, we might ourselves conceivably be considered as virtual patients), and electronic case studies delivered via interactive computer applications.	Begg M. Virtual patients: practical advice for clinical authors using Labyrinth. Clin Teach. 2010;7(3):202-5.
Many factors play a role in the successful educational use of a Virtual Patient Simulation application. To start with, producing a Virtual Patient Simulation system (VPS) is a resource consuming endeavor. [...]There is a broad literature consensus in regarding ''clinical reasoning'' as the best use of VPS[...]It is thus not a surprise that VPSs are ''barely now entering the mainstream of medical education. Among the reasons cited there are early design flaws, use of improper educational models, high up-front costs, and lack of proof of educational efficacy	Botezatu M, Hult H, Kassaye Tessma M, Fors UG. As time goes by: Stakeholder opinions on the implementation and use of a virtual patient simulation system. Med Teach 2010;32(11):e509-16.
Virtual patients (VPs) are used increasingly in medical education, especially to teach clinical reasoning.	Huwendiek S, de Leng BA. Virtual patient design and curricular integration evaluation toolkit. Med Educ 2010;44(5):519.
Virtual patients (VPs) used as interactive, multimedia-enhanced case simulations may provide a feasable tool for student preparation.	Lehmann R, Bosse HM, Huwendiek S. Blended learning using virtual patients and skills laboratory training. Med Educ. 2010;44(5):521-2.
E-learning with virtual patients is where a student-user learns through simulation cases by playing the role of the physician responsible for treating patients.[...] However, e-learning programs that include patient simulations remain scarce as they are expensive and timeconsuming to create.	Horstmann M, Renninger M, Hennenlotter J, Horstmann CC, Stenzl A. Blended E-learning in a Web-based virtual hospital: a useful tool for

	undergraduate education in urology. Educ Health (Abingdon). 2009;22(2):269.
Virtual patients (VPs) are excellent teaching tools for teaching clinical medicine, developing decision-making and improving clinical competency but are believed to be very expensive and time consuming to make. [..] One response to these challenges has been the development of simulated or virtual patient (VP) encounters employing a range of technologies and software to replicate common or important patient presentations. VPs can provide students with a reliable, safe and repeatable environment in which to rehearse and practise diagnostic skills and develop clinical reasoning. In particular, VPs have demonstrated their use in healthcare teaching, learning and assessment and throughout a wide range of designs for learning (Ellaway et al. 2006). Typically VPs offer task repetition, the ability to investigate alternative courses of action, the ability to make mistakes without real world repercussions, more exposure to patient scenarios, learning at a time, place, and pace convenient to the learner and formative or summative assessment with consistent feedback. Whilst VPs are a useful component of healthcare education, they are not a cheap alternative.	Round J, Conradi E, Poulton T. Training staff to create simple interactive virtual patients: the impact on a medical and healthcare institution. Med Teach 2009;31(8):764-9.
Web-based clinical cases (‘‘virtual patients’’, VPs) provide the potential for valid, cost-effective teaching and assessment of clinical skills, especially clinical reasoning skills, of medical students.[..] There is a growing need to develop assessment methods for clinical reasoning and decision-making. One such method includes the use of VPs for assessment.	Gesundheit N, Brutlag P, Youngblood P, Gunning WT, Zary N, Fors U. The use of virtual patients to assess the clinical skills and reasoning of medical students: initial insights on student acceptance. Med Teach 2009; 31(8):739-42.
Use of Virtual Patients (VPs) in medical education provides opportunities for students to train on a variety of disease scenarios at an accelerated pace from real-life patient encounters. These educational tools can expose students to events not witnessed in clinical training settings. However, while VPs can theoretically expand the number of case situations experienced by the learner, they are expensive to create, from both a time and effort perspective. [...] Institutional sharing of VPs would increase accessibility to the student of a broad range of VPs with different disease scenarios and more diversity of patients and cultural environments without duplication of effort. Efforts are underway to enable greater sharing and collaboration of VPs between institutions on an international basis through common data interoperability standards. But sharing of VPs outside the local classroom context highlights the need for strong management of the rights and permissions associated with the VP content. While VPs vary widely in terms of how they deliver simulated patient case information, they all incorporate some type of content. This content may include pictures, audio and/or video recordings, as well as scenarios. The VPs may be the creation of one person or a collaboration of many people, working with newly authored content, pre-existing ‘re-purposed’ content, or a combination thereof. The platforms on which the VPs operate may also be an original development or adopted from an existing system. In all these situations, intellectual property rights (IPRs) arise. How these rights are managed by the VP development team at the beginning of the project has considerable	Campbell G, Miller A, Balasubramaniam C. The role of intellectual property in creating, sharing and repurposing virtual patients. Med Teach. 2009;31(8):709-12.

implications on whether the VP can be shared and repurposed.	
On-screen simulations of clinical settings have been used for educational purposes since the 1970s. Despite this, it is only now that these 'virtual patients' are increasingly forming a part of the medical education mainstream. [...] The value of virtual patients in medicine and healthcare learning has been attributed to their potential for use in a wide range of distinct educational contexts. These uses include the exposure of students to a wider range of patient scenarios than they are likely to encounter face-to-face during their training, the provision of safe alternatives to novices engaging in direct clinical practice, and the use of virtual patients as assessment tools. Of course, all of these applications could also be (and in many cases are being) mediated by more traditional methods, such as simulated patient programs. However, it is increasingly recognized that a particular strength of virtual patients is to promote and/or assess clinical reasoning (Cook 2009), particularly in complex and emergent settings. In other words, virtual patients are uniquely suited to offer structured training in the type of clinical decisionmaking that experts normally acquire only after considerable experience and practice, and to do so in a safe and controlled environment. [...] Despite their many strengths and advantages, there are also challenges in developing and using virtual patients. As with any design for learning, virtual patient activities need to be tailored to the particular contexts in which they are to be used. A gradual lessening of barriers to adoption and wider changes in the educational environment have led to virtual patients becoming increasingly practical and desirable as an educational modality. Important factors in this change include[...] Despite there being quite different approaches to design, previous evaluations of virtual patient systems have tended to be based on single favoured institutional designs or systems, with little or no consideration of the variety of other approaches or the partiality that such monocultures impose. The move to the collaborative development and implementation of common standards and specifications, in particular the MedBiquitous Virtual Patient standard, has provided a powerful stimulus to go beyond local orthodoxies to develop shared understanding and practice.	Ellaway RH, Poulton T, Smothers V, Greene P. Virtual patients come of age. Med Teach. 2009; 31(8):683-4.
Virtual-patient CAI modules also provide students with the added benefits of problem-based learning (PBL) approaches. Moreover, virtual patient CAI modules provide pre-clerkship medical students the opportunity to assimilate learned theory in a controlled patient setting before embarking on the care for live patients.	Kandasamy T, Fung K. Interactive Internet-based cases for undergraduate otolaryngology education. Otolaryngol Head Neck Surg 2009; 140(3):398-402.
Virtual patients (VPs), or computerized representations of patients, have the potential to provide medical students with a safe learning environment, the opportunity for extensive repetitive practice, and real-time feedback. Moreover, VPs could facilitate the learning of anxiety-provoking tasks such as breast HT and examination before interacting with a RP or SP.	Deladisma AM, Gupta M, Kotranza A, Bittner JG 4th, Imam T, Swinson D, Gucwa A, Nesbit R, Lok B, Pugh C, Lind DS. A pilot study to integrate an immersive virtual patient with a breast complaint and breast examination

	simulator into a surgery clerkship. Am J Surg 2009; 197(1):102-6.
The virtual patient scenario provides a detailed account of a patient's illness and care, using multimedia and computing technology. It allows the student to follow a patient's progress from beginning to end, which is often not possible with real patients and within the time constraints of modern-day medical curricula. Such scenarios can also be made formative and interactive by embedding questions and feedback within the web pages. Thus, the scenario becomes a type of interactive e-learning teaching resource.	Critchley LA, Wong JW, Leung JY. Virtual patients and undergraduate anaesthesia teaching. Med Educ 2008; 42(11):1120-1.
providing a simulated (virtual patient) clinical exposure for students	Sanders C, Kleinert HL, Boyd SE, Herren C, Theiss L, Mink J. Virtual patient instruction for dental students: can it improve dental care access for persons with special needs? Spec Care Dentist. 2008;28(5):205-13.
Over recent years, we have seen an increasing level of use of simulated and virtual patients (computer-based simulations of patients) for both training and assessment in medical education. This has been extensively reported by Issenberg et al. in a BEME systematic review (guide no 4) where both problems and opportunities were thoroughly investigated. Studies investigating the usefulness of simulations for instance, have showed that students might react similarly to real and simulated patients. In the study by Edelstein et al., students thought that computer-based case simulations were better tests of clinical decision making than written shelf examinations. Schuwirth & Van der Vleuten investigated the problem of construct and face validity and described how to meet the need for assessment procedures that are both authentic and well-structured. In their educational assessment guidelines, Appel et al. even recommend the Clerkship Directors to 'use computer-based case simulations to augment traditional internal medicine evaluation methods' and that these 'would be used as a supplement to, and not a replacement for, other assessment tools'. In a special themed article, Holmboe states that simulated patients and other simulation technologies are considered as 'being important and reliable tools for teaching clinical skills and evaluating competence' but also emphasises that 'they cannot substitute to the direct observation by faculty of trainees' clinical skills with actual patients. Hence, because of deficiencies in faculty direct observation evaluation skills, automated scoring of patient interactions has been proposed as a way to limit the effort required for mentor evaluation. Simulated patients have therefore been suggested to be useful as assessment tools in Objective Structured Clinical Examinations (OSCEs) or in other assessment methods in evaluating students' interactions with patient related medical issues, such as clinical reasoning and/or medical problem solving abilities. More recently, virtual patients (VP) have been gradually introduced as a complementary method to simulated patients because they support active and reflective learning.	Courteille O, Bergin R, Stockeld D, Ponzer S, Fors U. The use of a virtual patient case in an OSCE-based exam-a pilot study. Med Teach 2008;30(3):e66-76

Virtual patients (VPs) have the potential to augment existing medical school curricula to teach history-taking and communication skills. [...] Virtual patients (VPs) have the potential to enhance the efforts of health professions students in learning history-taking and communication skills by providing a means for standardized, repetitive practice in a safe environment. [..]With respect to medical education, VPs can elicit empathetic responses from students when compared to standardized patients (SPs) and can help reduce student anxiety when performing sensitive topic interviews, such as the sexual history, for the first time	Deladisma AM, Johnsen K, Raij A, Rossen B, Kotranza A, Kalapurakal M, Szlam S, Bittner JG 4th, Swinson D, Lok B, Lind DS. Medical student satisfaction using a virtual patient system to learn history-taking communication skills. Stud Health Technol Inform 2008;132:101-5.
Interactive multimedia instruction, involving computer-assisted training or the virtual patient instructional format provides an alternative to hands-on clinical experience and has demonstrated efficacy in a variety of domains, including clinical nursing education. In fact, computer-assisted instruction (e.g., the virtual patient format) has been found to have the potential to surpass traditional methods of instruction in educational gains. [..] The virtual patient format affords students risk-free practice of clinical skills and judgment. Multimedia, interactive instructional modules allow students to work at their own pace, usually providing immediate feedback. Moreover, as noted by Kiegaldie & White, interactive instructional modules allow for scaffolding of student learning by organizing the case information in a coherent fashion. The format allows students to work at their own pace and provides prompt feedback.	Sanders CL, Kleinert HL, Free T, King P, Slusher I, Boyd S. Developmental disabilities: improving competence in care using virtual patients. J Nurs Educ 2008;47(2):66-73.
Virtual patients are nearer to reality than multiple choice questions or paper cases.[..] A clinical scenario is described and the student – in the role of the physician – has to deal with a ''virtual patient''.	Waldmann UM, Gulich MS, Zeitler HP. Virtual patients for assessing medical students--important aspects when considering the introduction of a new assessment format. Med Teach. 2008;30(1):17-24.
Interactive multimedia instruction, involving computer-assisted training and/or a "virtual patient" (VP) instructional format provides an alternative to hands-on clinical experience with demonstrated efficacy in a variety of domains, including clinical nursing education. In fact, CAI (e.g., VP format) has often been found to surpass traditional methods of instruction in educational gains.	Sanders CL, Kleinert HL, Free T, Slusher I, Clevenger K, Johnson S, Boyd SE. Caring for children with intellectual and developmental disabilities: virtual patient instruction improves students' knowledge and comfort level. J Pediatr Nurs. 2007;22(6):457-66.
Experimental use of patient simulations in medical education has been going on for the past twenty-five years. The purpose of these simulations is to offer students a virtual patient on whom they can practice medical history taking, physical examination, laboratory and functional tests ordering and assessment, inferring differential diagnoses and treatment planning. A student is provided with different kinds of feedback generated within the virtual learning simulation environment.	Beux PL, Fieschi M. Virtual biomedical universities and e-learning. Int J Med Inform. 2007;76(5-6):331-5

For simulation to be effective, it must expose the student to virtual patients that demonstrate sophisticated, realistic behaviors; it must allow openended patient investigation by the student (learning through self-discovery); and it must provide each student with a population of patients suffering from a given disease, with each patient displaying clinically relevant variations on the disease theme. Such variations might involve the path or speed of disease progression, the profile and severity of symptoms, responses to treatments, and secondary diseases or disorders that affect treatment choices. If each student could independently manage the care of many such patients – especially in a context in which trial and error learning carried no risk – we hypothesize that the decision making skills of each student would develop faster than with traditional training methods alone.	Jarrell B, Nirenburg S, McShane M, Fantry G, Beale S, Mallott D, Raczek J. An interactive, cognitive simulation of gastroesophageal reflux disease. Stud Health Technol Inform 2007; 125:194-9.
Clearly, much remains to be done in preparing general dentists to care for patients with developmental disabilities. The lack of availability of an adequate patient pool to practice clinical skills has been cited as one barrier to the development of proficiency in this practice area. For many developmental disabilities (e.g., Down syndrome, deaf-blindness), students may simply not have the opportunity to provide treatment during their training. Interactive, multimedia, computer-based, virtual patient instruction provides an alternative to hands-on experience and has demonstrated consistent efficacy in a variety of educational domains, including clinical training in the health professions. For example, Frisby et al. found that a computer-based multimedia program consisting of an infant patient encounter was more effective in teaching physical examination skills to medical residents than routine didactic instruction. Interactive, computer- assisted virtual patient instruction offers several advantages over traditional instruction. Virtual patient encounters offer students the opportunity to practice clinical decision-making skills in an environment that poses no danger to either student or patient. An interactive format typically allows students to learn at their own pace, and the format provides for prompt feedback, which supports student metacognition.	Kleinert HL, Sanders C, Mink J, Nash D, Johnson J, Boyd S, Challman S. Improving student dentist competencies and perception of difficulty in delivering care to children with developmental disabilities using a virtual patient module. J Dent Educ. 2007;71(2):279-86.
A working definition of a virtual patient is: An interactive computer simulation of real-life clinical scenarios for the purpose of medical training, education, or assessment. Users may be learners, teachers, or examiners.	Ellaway R, Candler C, Greene P, Smothers V. An Architectural Model for MedBiquitous Virtual Patients. White paper available from http://groups.medbiq.org/medbiq/display/VPWG/MedBiquitous+Virtual+Patient+Architecture
'Problem solving' virtual patients have a relatively generic design that simulates the stages of a real-patient encounter; beginning from interview and proceeding to physical exam and then to gathering lab and imaging data and finally management according to the most probable diagnosis.	Vash JH, Yunesian M, Shariati M, Keshvari A, Harirchi I. Virtual patients in undergraduate surgery education: a randomized controlled study. ANZ J Surg. 2007; 77(1-2):54-9.
In order to prepare students for the reality of patient care, a longitudinal approach involving complex cases is recommended. One means of accomplishing this is to create a scenario in which the patient's condition changes with every visit. These changes in patient response to care may be related to the student's drug therapy recommendation, a disease complication, or deterioration in organ function. Changes commonly require reevaluation of current therapy, dosage modifications, or ordering laboratory tests. The application of virtual	Hussein G, Kawahara N. Adaptive and longitudinal pharmaceutical care instruction using an interactive voice response/text-to-speech system. Am J Pharm Educ 2006;70(2):37.

patients who change in ways appropriate to the recommendations students make creates scenarios that more closely parallel the reality of pharmaceutical care.	
Computer-based virtual patients (VPs) are now being explored as a new method for creating high-fidelity simulated patient interactions that can overcome many of the challenges associated with using live SPs. As computer-based VPs can be used at any time, they can be integrated into curricula in a much more flexible manner. Many learners can use a single VP case simultaneously. Virtual patients offer true standardization across interactions creating a more consistent but less flexible experience for learners. The VP has the advantage of being easily modified to demonstrate a variety of clinical or interview scenarios, for example changing the gender or race of the patient. Virtual patients have worked well in simulating concrete and predictable topics, such as physiological processes and performing procedures. More recently they have been used to model the patient interview and are being used to teach clinical interviewing skills. Previous work has included the development of VP cases designed to teach bedside competencies of bioethics, basic patient communication and history taking, and clinical decision making. Combining live SPs and VPs to create more realistic simulations of procedures or he effects of medications has also been explored.	Triola M, Feldman H, Kalet AL, Zabar S, Kachur EK, Gillespie C, Anderson M, Griesser C, Lipkin M. A randomized trial of teaching clinical skills using virtual and live standardized patients. J Gen Intern Med. 2006; 21(5):424-9.
One way to provide learners with the opportunity to train medical problem solving and clinical reasoning in a relevant context is by simulating patient encounters using either standardized patients [7,8] or Virtual patients (VP). VP can be put into two broad categories: problem-solving or narrative [10,11]. These categories represent the most frequent design choices used when developing VP systems. The problem-solving approach is aimed for learning and training clinical reasoning or diagnosis. Problembased learning, or exploratory learning, often underpins this design. In such a system, the student gathers information, usually from menus of possible history questions, lab tests, and phsysical examinations, and subsequently diagnoses and/or manages the patient. The information is not "cued", that is, there is no direction from the program format as to what the student's next course of action should be. Templates are easily created with this type of design, which might reduce the costs of multiple simulations [12-14]. The narrative approach is often found in VP systems that are concerned with cause and effect and that have a time element/personal story line [15]. Using this approach, learning and training medical problem solving and clinical reasoning might be harder to accomplish in a problem based setting. The main features of VP systems are that they allow for repetitive and deliberate practice of "clinical" skills by any learner (e.g. nurse, physician, dentist students) without regard to time of day, physical location, or position in the health science curriculum. VP provides practice in a safe environment with no risk to patient or student. Mistakes are allowed. Another benefit of the simulated environment is the ability to allow every student the opportunity to "meet" any disease they may encounter in their practice by providing cases optimized for medical education. Some conditions are so rare that it would take a lifetime to gain experience, while others that are common, are handled by the primary care and do not turn up at university hospitals. Record keeping, reproducibility, assessment and validity	Zary N, Johnson G, Boberg J, Fors UG. Development, implementation and pilot evaluation of a Web-based Virtual Patient Case Simulation environment-Web-SP. BMC Med Educ 2006; 6:10.

are issues all brought to the forefront with clinical governance and revalidation. VP could be a way of addressing some of these issues. The World Wide Web has become more and more important as an effective medium to grant access to VP and other learning systems. VP are directly and always available, changes made in the cases are immediately accessible and the users do not have to carry out extensive installations. The Web as a distribution method might also have some drawbacks and limitations like bandwidth limitation that might influence the effective use of multimedia, especially larger video clips. [..] Issenberg et al. lists the factors that might boost the future role of simulation technology in the education of health professionals such as demonstrated educational effectiveness, increased technological efficiency and simplicity, educational cost-effectiveness, and slow evolution in the culture of clinical education. What might prevent a more widespread implementation of web-based VP systems in health education includes among other things: • The absence of a common generic platform for design/ creation, management and evaluation of web-based patient cases. • A poor integration of simulation-based education into health sciences curricula due to the inability to move beyond the technology savvy teachers. The lack of rigorous evaluation research to fine-tune the technology and gauge its outcomes. • The dependence on the assistance of computer specialists to develop realistic and interactive cases. • The lack of tools to facilitate the exchange of patient cases between teachers.	
Virtual patients have great potential for training patient-doctor communication skills. [..] Virtual patients are receiving serious attention as a powerful tool for educating medical students. Through repeated experiences with virtual patients, medical students can be exposed to, and evaluated on, many more situations then through traditional methods. [...] Each of the core components: graphics rendering, speech recognition, speech processing, and speech synthesis play a part in the overall impression of a virtual human.	Dickerson R, Johnsen K, Raij A, Lok B, Stevens A, Bernard T, Lind DS. Virtual patients: assessment of synthesized versus recorded speech. Stud Health Technol Inform 2006;119:114-9.
Virtual patients are a fairly easy bunch to get along with. You make up their identity and their symptoms and then ask your learners about their diagnosis. If they do not bring out the teaching points that you wanted them to, then you can change their symptoms.	Walsh K. Virtual patients get real. Med Educ 2005; 39(11):1153-4.
Often the appropriate patient is missed or he can not be demonstrated to all students because of practical or ethical problems. A possible solution is the presentation of a clinical problem by a virtual patient using a simulative computer program. But such an approach means often a lot of work for the author of the case.	Ruderich F, Bauch M, Haag M, Heid J, Leven FJ, Singer R, Geiss HK, Jünger J, Tönshoff B. CAMPUS-a flexible, interactive system for web-based, problem-based learning in health care. Stud Health Technol Inform 2004;107(Pt 2):921-5.
It is difficult to track how many medical schools have created virtual patient panels or virtual clinics, but Minnesota's apparently is one of only a few such programs in the United States-or the world, for that matter. [...] The use of virtual patients "comes close to replicating what it is we really expect a doctor to do," says Smith. The concept teaches students how to access online patient care information from their first ay of medical school. "It helps them develop the skills of being lifelong learners," Smith says. Danoff says medical schools that are considering the use of virtual patients have to weigh the benefits—flexibility and cases that fit well with course	Voelker R. Virtual patients help medical students link basic science with clinical care. JAMA. 2003; 290(13):1700-1.

material - with potential drawbacks of cost and having the information technology capabilities to set up clinic Websites .	
medical student role-playing a doctor with a computer- based, simulated patient. However, such a ‘‘virtual patient’’ is often represented as a case rather than as a person.[...] virtual patients can be put into two broad categories: problem-solving and narrative. [..] The problem-solving approach teaches clinical reasoning or diagnosis. Problem-based learning or exploratory learning often underpins this design choice. The student gathers information, usually from menus of possible history questions, lab tests, and physical examinations, and subsequently diagnoses and/or manages the patient. The information is not ‘‘cued,’’ that is, there is no direction from the program format as to what the student’s next course of action should be. Templates are easily created with this type of design, which reduces the costs of multiple simulations. [..] The narrative approach is often found in virtual patient encounters that are concerned with cause and effect and that have a time element. In general, narrative designs are more expensive to produce because the narrative has to be individually crafted -a time-consuming process-rather than relying on a template. Pedagogic rationales are diverse but narrative designs often have a personal story line.	Bearman M. Is virtual the same as real? Medical students' experiences of a virtual patient. Acad Med. 2003;78(5):538-45.
Virtual patients - where the student role-plays a doctor with a computer-based simulated patient... They range from simple web-based to expensive resource-intensive productions, but there are also differences in their fundamental structure. Two majpr designs have been distiguished previously by the authors. These are "problem-solving" and the "narrative" approach. The problem-solving approach is found in virtual patient designs concerned with teaching clinical reasoning and diagnosis. Generally the student has to collect a range of uncued information - usually from menus of possible history questions, lab tests and physical examinations - and make descisions based on their findings. [..] Often the problem-solving design is very cost-effective, as templates are easy to build and maintain, which greatly reduces the cost of creating multiple virtual patients. The narrative approach is often found in virtual patioent encounters that are concerned with cause and effect. This includes programs that have an emphasis on decision making, resulting in various outcomes over time. Creating a decision tree can be very time-consuming, and in general narrative designs are more expensive to produce because the narrative has to be individually crafted than relying on a template. [...] It can therefor be hypothesised that a narrative approach to the virtual patient will provide an increased sence of value of the patient as a person, with an accomanying affective dimension. [...] There is empirical evidence for the value of using narrative virtual patients to teach communication skills.	Bearman M, Cesnik B. Comparing student attitudes to different models of the same virtual patient. Stud Health Technol Inform 2001;84(Pt 2):1004-8.

Two types of virtual patient designs can be distinguished: a `narrative' structure and a `problemsolving' structure. [..] Virtual patients are an increasingly common tool in medical education and are used across a variety of clinical disciplines. They range from the simple webbased to the expensive resource-intensive productions, but there are also differences in their fundamental structure. After a wide range of virtual patients had been examined, two major designs were distinguished. These are the `problem-solving' and the `narrative' approaches. The problem-solving approach is found in virtual patient designs concerned with teaching clinical reasoning and diagnosis. Generally the student has to collect a range of information, usually from menus of possible history questions, lab tests, and physical examinations, and make diagnostic and management decisions based on their findings (Figure 1). The students have to decide what is relevant: the information is not cued. Frequently the pedagogic rationales are of exploratory or problem-based learning. [..] This type of design is easily extended to templates, which enormously reduce the cost of creating multiple virtual patients. [...] The narrative approach is often found in virtual patient encounters which are concerned with cause and effect. This includes programmes that have an emphasis on decision making which results in various outcomes over time. Creating a decision tree for narrative virtual patients can be very time-consuming, as Rosenblatt notes in his report on the Virtual Patient Project.3 In general `narrative' designs are more expensive to produce because the narrative has to be individually crafted, rather than created using a template. Henderson's pedagogic rationale for simulation using this type of design is that of reflective learning through experience. [...] Narrative is considered a method of integrating the sense of the human into medical education.	Bearman M, Cesnik B, Liddell M. Random comparison of 'virtual patient' models in the context of teaching clinical communication skills. Med Educ. 2001; 35(9):824-32.
In the future we will see more sophisticated software with virtual patients who can communicate and interact with the student in a very realistic way. The software will even ‘‘step out’’ from the screen and help the student with clinical procedures. However, at present CAL should not replace traditional education, but rather be used more as a supplement and for self-directed studies.	Schittek M, Mattheos N, Lyon HC, Attström R. Computer assisted learning. A review. Eur J Dent Educ. 2001;5(3):93-100
Multimedia applications can achieve not only real-time connections, but can help construct a "virtual patient" as a platform for supervision and assessment, permitting preceptors to evaluate traineepatient interactions, utilization of Web-based data and human resources, and on-line professionalism. [...] In this model the electronic record will become the `virtual patient', building, of course, upon individual real-time, person-to-person interactions. Digital transmission and storage permit video, audio, text, and telemetry of data to be merged into one medium, creating a rich, integrated, patient record capable of ubiquitous transfer and access via the internet.	Glick TH, Moore GT. Time to learn: the outlook for renewal of patient-centred education in the digital age. Med Educ 2001;35(5):505-9.
Interactive multimedia instruction (such as CD-ROM–based programs), including computerassisted instruction, and/or virtual patient modules, provides an alternative to hands-on clinical experience and has demonstrated efficacy in a variety of domains, including medical education. computer- assisted instruction (eg, “virtual patient” format) has demonstrated the potential to surpass traditional methods of instruction in educational gains. Certain patients with developmental disabilities may present with issues too complex for students in early stages of clinical exposure to ethically provide care — the “virtual patient” format thus affords students risk-free practice in clinical skills and judgment. A multimedia, interactive instructional module lets students work at their	Kleinert HL, Fisher SB, Sanders CL, Boyd S. Improving physician assistants students' competencies in developmental disabilities using virtual patient modules. Journal of Physician Assistant Education 2007;18(2): 33-40.

own pace, while typically providing immediate feedback.	
narrative-based environments such as virtual patients	Ellaway R,Topps D. Preparing for practice: Issues in virtual medical education. Wankel, Charles [Ed]; Malleck, Shaun [Ed]. (2010). Emerging ethical issues of life in virtual worlds. (pp. 101-117). viii, 222 pp. Charlotte, NC, US: Information Age Publishing; US.
Web based e-learning with virtual patient cases has become a valuable tool in medical education. [...] With continuing advances in computer and internet technology web based e-learning with virtual patients becomes an option even for undergraduate medical students	Horstmann M, Horstmann C, Renninger M. Case creation and e-learning in a web-based virtual department of urology using the INMEDEA simulator. Nephro-Urology Monthly 2012;4(1):356-60.
In medical applications, Virtual humans would constitute natural interfaces to knowledge-based systems, as virtual patients displaying the symptoms associated with a given pathology.	Cavazza M, Simo A. A virtual patient based on qualitative simulation. International Conference on Intelligent User Interfaces, Proceedings 2003;19-25
In this paper we present a new model of collaborative virtual environment for medical e-learning, this model is based on virtual patient methodology. The virtual patient provides a semi real environment which can be used for students training and assessment. The teacher will be able to create a medical case represented in a virtual patient, which can be hardly achieved in the real time. These medical cases can be accessed through a web based application that provides a set of interactive multimedia tools that can be used in diagnose any virtual patient. Either in training mode (lectures) which can be recorded and accessed later by medicine students, or in assessment mode which enables the teacher create a virtual practical assessment.	El-Razek SMA, El-Bakry HM, El-Wahed WFA, Mastorakis N. Collaborative virtual environment model for medical E-learning. Proceedings of the 9th WSEAS International Conference on Applied Computer and Applied Computational Science, 2010;191-195
Interactive Virtual Standardized Patients (VP) can provide meaningful training for clinicans. These VP's portray interactive embodied conversational characters with realistic representations of a mental or physical problem to be diagnosed or discussed.	Kenny PG, Parsons TD, Gratch J, Rizzo AA. Evaluation of novice and expert interpersonal interaction skills with a virtual patient. Lecture Notes in Computer Science 2009;5773:511-2
Interactive computer generated characters can be applied to the medical field as virtual patients for clinical training. The user interface for the virtual characters takes on the same appearance and behavior as a human.[...] Virtual humans are embodied interactive agents that represent real humans in a virtual environment. These avatar characters take on human representations in their appearance, interaction and decision making and are used in many applications that require human-like interfaces, such as guides, trainers or medical. [...] The focus of the	Kenny PG, Parsons TD, Rizzo AA. Human computer interaction in virtual standardized patient systems. Lecture Notes in Computer Science 2009;5613:514-23

virtual patient is applying these virtual humans to the medical domain to create virtual standardized patients (VP) that can be used to teach interview, diagnosis, and social-interaction skills.	
Thus, to study VHs, we have created and refined a constrained scenario involving a virtual patient (VP) - a digital animated avatar, or DIANA that medical students interview.	Lok B. Teaching communication skills with virtual humans. IEEE Computer Graphics and Applications 2006;26(3):10-3
The virtual patient is a "double agent" that displays both physiological and cognitive function. Physiologically, it undergoes both normal and pathological processes in response to internal and external stimuli. Cognitively, it experiences symptoms, has lifestyle preferences, has memory (many of whose details fade with time), and communicates with the human user about its personal history and symptoms.	McShane M, Nirenburg S, Beale S, Jarrell B, Fantry G. Knowledge-based modeling and simulation of diseases with highly differentiated clinical manifestations. Lecture Notes in Computer Science 2007;4594:34-43
Virtual patients (VPs) in MVP are "double agents" combining a physiological agent that simulates the progression of a disease with a cognitive agent capable of perceiving symptoms, understanding natural language inputs, reasoning (notably, making decisions about its actions) and carrying out verbal actions. The language capabilities allow the VPs not only to engage in dialog but also to learn by being told. The VPs can learn both general facts (for example, the properties of a disease) and specific facts (for example, that their test results are negative).	Nirenburg S, McShane M, Beale S. Aspects of metacognitive self-awareness in Maryland virtual patient. AAAI Fall Symposium - Technical Report FS-10-01:69-74
At the core of this network is the virtual patient (VP) – a knowledge-based model and simulation of a person suffering from one or more diseases. The virtual patient is a "double agent" in that it models and simulates both the physiological and the cognitive functionality of a human. Physiologically, it undergoes both normal and pathological processes in response to internal and external stimuli. Cognitively, it experiences symptoms, has lifestyle preferences (a model of character traits), has memory (many of whose details fade with time), and communicates with the human user about its personal history and symptoms. [...] The virtual patient is a "double agent" in that it combines the physiological agent, which is a simulation of the physiological and pathological properties of an organism, with a cognitive agent capable of perception, reasoning, and communication in natural language.	Nirenburg S, McShane M, Beale S. A simulated physiological/cognitive "Double Agent". AAAI Fall Symposium - Technical Report FS-08-04():127-134
Maryland Virtual Patient (MVP) is a cognitive simulation and training system whose goal is to provide medical practitioners with the opportunity to develop clinical decision-making skills by managing many highly differentiated artificial intelligent agents playing the role of virtual patients (VPs). These VPs can suffer from various diseases and combinations of diseases, and are capable of realistic physiological and cognitive responses even to unexpected actions on the part of the user. [...] In short, trainees can learn by trial and error using a large number of patients that present with clinically relevant variations of each disease.[...] The core artificial agent, the VP, is a knowledge-based model and simulation of a person suffering from one or more diseases. The VP is a "double agent" in that it models and simulates both the physiological and the cognitive functionality of a human. Physiologically, it undergoes both normal and pathological processes and responds realistically both to expected	Nirenburg S, McShane M, Beale S, English J, Catizone R. Four kinds of learning in one agent-oriented environment. Frontiers in Artificial Intelligence and Applications 2010; 221:92-97

and to unexpected (e.g., by user error) internal and external stimuli. Cognitively, it experiences symptoms, has lifestyle preferences (a model of character traits), has dynamic memory and learning capabilities, has the ability to reason in a contextsensitive way, and can communicate with the human user about its personal history, symptoms and preferences for treatment.	
Virtual patient case studies allow students to take a case history from an imaginary patient, order virtual tests, and explore different diagnoses and treatments. An imaginative story line, illustrated by pictures or video clips can make these cases convincing and effective in accustoming the future doctor to the decisions he must take while dealing with a real patient. In the view of better professional qualification for medical students, a simulation type virtual patient e-learning system can clearly be considered the best way of improvement.	Scarlat R, Stanescu L, Popescu E, Burdescu DD. Case-based medical E-assessment system. Proceedings - 10th IEEE International Conference on Advanced Learning Technologies, ICALT 2010: 158-162
the development of a virtual patient can be seen as an integration of a visual model and a physiological model, which is also a realistic model of the “internal behaviour” of the patient.	Simo A, Cavazza M. Qualitative Simulation of Shock States in a Virtual Patient. LECTURE NOTES IN COMPUTER SCIENCE 2003; 2780:101-11
The literature supports the educational advantages of actors being employed as simulated patients on both tutorial practice sessions and for assessments such as oral examinations, with significant improvements being seen in student’s social and history-taking skills . This is true even if there is no direct perception of the simulated patient as being human, that is, when the patient becomes “virtual”. [...] Computerised simulation of patients in medicine, dentistry, pharmacy, or nursing personalise the patient by employing images of a patient that are either static pictures of real patients, or dynamic three-dimensional images (either video images of real patients or computer generated images using virtual reality technology and avatars to represent patients). Computerised virtual patient systems have been developed to assist the generation of case studies that will be presented by a computer to a student for the student’s diagnosis and which will then provide feedback to the student. [..] Despite the limitations of technology illustrated above, there are many advantages in using computerised virtual patients rather than actors. These include savings in the cost and time of training, and in the cost of employing, real actors. Advantages are also realised in the standardisation of the assessment, with greater control over the assessment process and greater consistency of student feedback than is possible using real actors. Computerised virtual patients are available at any time and can be utilised for many assessments occurring simultaneously at many different locations. They can also easily record student performance and generate reports on individual student, or on aggregated class, results for both summative and formative assessments.[...] Dynamic interaction is also achieved using virtual reality avatars, which are computer generated animations used as representations of patients	Summons PF, Newby D, Athauda R, Park M, Shaw P, Pranata I, Jin JS, Xu YD. Design strategy for a scalable virtual pharmacy patient. ACIS 2009 Proceedings - 20th Australasian Conference on Information Systems :96-110
Many different entities with unique approaches and attributes are all often called virtual patients. Such approaches include case presentations, virtual patient games, human standardized patients, high fidelity simulations, high fidelity mannikins and virtual human conversational agents.	Talbot TB, Sagae K, John B, Rizzo AA. Sorting out the virtual patient: How to exploit artificial intelligence, game technology and sound educational practices to create engaging role-playing simulations. International Journal of

	Gaming and Computer-Mediated Simulations 2012;4(3):1-19
Opportunities for building learning activities around real patients have decreased and various representative simulations have become an increasingly common alternative. The use of virtual patients is one such simulation developed to support the delivery of clinical teaching. [...]Many high-quality e-learning materials are being produced by medical schools and healthcare organizations. 'Virtual patients' is one of the models developed to support the delivery of clinical teaching. Healthcare students are familiar with the concept of virtual patients, as they are frequently exposed to actors performing the role of patients in clinical examinations. In the area of medicine, however, there are limitations to what these cases can offer in terms of either a game-informed learning experience or a real patient experience, as the narratives that accompany and describe many current virtual patient scenarios are simplistic and linear. [...] Virtual patient scenarios offer opportunities for 'game-informed learning'. This is due to their experiential and problembased learning approaches as prime pedagogic drivers. Branching stories that represent virtual patient scenarios are not new in medical education. Some medical schools have successfully included their delivery across the medical curriculum, pointing out that they offer opportunities for 'game-informed learning'. They shift the emphasis from case-based scenarios towards a more controlled position in which the learner is able to steer the case. The reason for using game-based models is simple: people learn better when they don't know that they are learning. Gamebased learning tends to be a pleasant break from traditional linear content. As Begg et al. observed, the lack of an immersive contextual framework tends to fail to engage students within the activity. The authenticity of the environment and the value of the actions taken by the learner will reflect on the level of immersion and, therefore, the reality of the learning experience. However, development of three-dimensional representations is challenging, and it requires a lot of information in order to create a credible 'metaverse'. It is believed that branching virtual patient scenarios offer a more challenging and engaging learning experience that the learner can relate to; however, they lack immersion. This lack of immersion in current virtual patient delivery, as well as the familiarity of our 'digital natives' with virtual and game-based environments, has been the motivation for this piece of research.	Toro-Troconis M, Mellstrom U, Partridge M, Meeran K, Barrett M, Higham J. Designing game-based learning activities for virtual patients in second life. Journal of Cyber Therapy and Rehabilitation 2008; 1(3):225-38
Simulation in medical training appears to take four general forms: [...] virtual patients who are generally presented via computer-controlled, multimedia displays [...] Interactive software simulations of patients have been used in standard simulation exercises and in gaming simulations for training and assessing medical skills. These simulations are gradually taking the place of standardized patients, although the absence of strong artificial intelligence, which would allow full mixed-initiative dialogue, limits their applicability. However, growth in the use of virtual patients is likely to continue because of their ability to scale inexpensively to large numbers of physically dispersed learners, adapt quickly to prior knowledge and other individual characteristics of learners, and be available anytime and anywhere via the global information infrastructure. [...] The advantages of using simulation in training may be summarized as follows: -Safety: Simulated lives and health can be jeopardized to any extent required for learning.-Economy: Simulated materiel, equipment, and other resources-physical or	Fletcher JD, Wind AP. Cost considerations in using simulations for medical training. Mil Med 2013;178(10 Suppl): 37-46.

fiduciary-can be used, misused, and expended as needed. -Visibility: Simulation can provide visibility in at least two ways. It can (1) make the invisible visible and (2) control the visibility of details allowing the learner to discern the forest from the trees or the trees from the forest as needed. -Time control: Simulated time can be sped up, slowed down, or stopped. It can also be completely reversed, allowing learners to replicate specific problems, events, or operational environments as often as needed. These advantages seem to be applicable in medical training and education as elsewhere. Overall, simulation can provide massive amounts of practice with feedback, exposing individuals or teams to realistic situations that in real-world settings would range from the impracticable to the unthinkable.	
Despite the significant resources required to develop and maintain virtual patient (VP) programs, little is known about why this innovation has been adopted and how it is implemented. [...] VPs are a form of computer-assisted instruction in which learners work through the steps of diagnosing and managing patients, making clinical decisions without the risk of causing patient harm. VPs may be used to meet educational objectives, comply with regulatory requirements, or compensate for the specific needs of individual institutions or clerkship sites. A metaanalysis of VP studies found that they are an effective method for teaching medical knowledge, clinical reasoning, and other skills.4 [...] Although they are increasingly used in clinical education, VPs require significant resources to develop and maintain [..]Single cases or small collections of VP cases have been implemented in a variety of health professions education programs.4 Technical standards for VPs have been developed,8 and case-authoring tools are available for educators to develop their own cases.9,10 Because of the time and effort required to develop and maintain high-quality VPs, collaborations in Europe11 and the United States7,12–14 have emerged to develop more extensive VP programs.[...] Once adopted, VPs may be used to substitute for an existing learning activity (elimination strategy), change or enhance an existing learning activity (integration strategy), or add on to existing learning activities without change or substitution (addition strategy). Multiple calls have been issued to study the implementation of VPs within the clerkship curriculum. [...] Online VP programs are currently used in at least 136 medical schools in the United States and other countries.	Lang VJ, Kogan J, Berman N, Torre D. The Evolving Role of Online Virtual Patients in Internal Medicine Clerkship Education Nationally. Acad Med 2013; 88(11).
Virtual patients (VPs) are defined as "interactive computer simulations of real-life clinical scenarios for the purpose of healthcare and medical training, education or assessment". This definition distinguishes the VPs from devices, human standardized patients, part task trainers and high fidelity manikins. VPs have become an established tool in healthcare teaching and assessment. In particular, VPs are suggested as the key technology that can develop the fundamental skill of clinical reasoning amongst students, allowing students to develop these skills to a similar level as that achieved whilst training with real patients. By emulating the role of the healthcare professional, the learner is provided with training opportunities to identify relevant information from a set of anonymous patient-related data, conduct physical exams, laboratory tests and make diagnostic and therapeutic decisions without any real world repercussions. VPs are reported to be a response to some of the current challenges in medical education such as the limited learning opportunities for observing the treatment process. Moreover, VPs "fill gaps in clerkships by exposing students to diseases that they would not otherwise experience	Stathakarou N, Zary N, Kononowicz AA. Beyond xMOOCs in healthcare education: study of the feasibility in integrating virtual patient systems and MOOC platforms. PeerJ 2014;2:e672

because of short clinical rotations and limited ambulatory care experiences". Although there is evidence to support the effectiveness of training clinical reasoning skills using VPs, they "play only one part in the development of skilled health professionals" and coordination with other instructional activities is suggested. Positive effects have been reported when VPs are used as an additional resource or as an alternative to traditional methods. A significant barrier that medical faculties often encounter in integrating VPs in their curriculum is the timely, costly and complex process of producing and authoring VPs. VP systems have been extended in the past in order to support content transfer and by that to enable the technical sharing of the VP cases among institutions. That was achieved by applying theMedBiquitous Virtual Patient standard (MVP).	
An attractive field of interactive training is the use of online virtual patients (VP), using pre- and post-operative scenarios. VP are defined as multidimensional model patients through which a user has to simulate communication, information gathering, and apply diagnostic reasoning in support of effective and cost-efficient surgical care. Medical education is continually evolving to use such novel internet-based technologies and VP have demonstrated fidelity and content validity. Moreover, VP are uploaded on a virtual world that is easily accessible through an internet connection.	Beyer-Berjot L, Patel V, Ziprin P, Taylor D, Berdah S, Darzi A, Aggarwal R. Enhanced recovery simulation in colorectal surgery: design of virtual online patients. Surg Endosc 2014
Virtual patients are interactive computer simulations of clinical scenarios for medical training.	Bediang G, Perrin C, Ruiz de Castaneda R, Kamga Y, Sawadogo A, Bagayoko CO, Geissbuhler A. The RAFT Telemedicine Network: Lessons Learnt and Perspectives from a Decade of Educational and Clinical Services in Low- and Middle-Incomes Countries. Front Public Health 2014;2:180
Virtual patient simulation has grown substantially in health care education [..] Virtual patient simulation has fewer of these resource constraints compared to mannequin-based simulation. It is capable of creating high-fidelity simulation by applying the features identified in a systematic review. With the capacity for exhibiting a high level of interactivity and realism, a wide range of clinical scenarios with guided reflection can be designed into the virtual patient simulation. In addition, it can cater to a large number of learners simultaneously and be used by learners repeatedly when needed. Being accessible anytime and anywhere, it can also be integrated into curricula in a more flexible manner. Although the use of virtual patient simulations have been widely adopted for training health professionals, more research is required to inform how to effectively design and integrate them into curricula	Liaw SY, Chan SW, Chen FG, Hooi SC, Siau C. Comparison of virtual patient simulation with mannequin-based simulation for improving clinical performances in assessing and managing clinical deterioration: randomized controlled trial. J Med Internet Res 2014;16(9): e214
Virtual Patients (VPs) are an important component of medical education. One way to reduce the costs for creating VPs is sharing through repositories [..] Virtual Patients (VPs) are defined as "specific type of computer-based programs that simulate real-life clinical scenarios; learners emulate the roles of health care providers to obtain a history, conduct a physical exam, and make diagnostic and therapeutic decisions". Today, VPs are an important component of medical education, but the amount of labor and expense involved in their creation and maintenance	Küfner J, Kononowicz AA, Hege I. Virtual patient repositories-a comparative analysis. Stud Health Technol Inform 2014;

is high. Addressing the issue of labor and expense, recent VP-related research has been focused on enabling faculties to share VPs to reduce effort and costs. This can be realized in different ways: To foster the exchange of VPs, the MedBiquitous Virtual Patient (MVP) standard has been developed and implemented over the past years. Moreover, medical schools have undertaken efforts to collaboratively develop and use VPs in the recent past. [..] Finally, web-based repositories allow faculties to either access VPs directly or download, adapt and further use them.	
... using simulated, standardized,and virtual patients provide a learning experience for students in the early pharmacy curriculum that is comparable to direct patient contact. As a result, educational technologies such as computer-aided instruction, virtual patients, and mannequin model simulators have become almost omnipresent in pharmacy education. [...] Virtual patient technology has been used by educators throughout the health sciences to develop required knowledge and skills. Virtual patients simulate real clinical scenarios and allow learners to emulate the roles of healthcare providers by obtaining patient information and making diagnostic and therapeutic decisions. Virtual patients offer educators the opportunity to assess students' decision-making skills regarding course content in a safe, high-fidelity environment, while allowing students to succeed, err, and learn individually. Despite these advantages, educational technologies like virtual patients can be underused in the curricula of higher education. Cultural, process, and academic obstacles to implementing such technologies in the classroom can be overwhelming and disabling. Specifically, attitudes of an institution towards technology and education must be positive, resources (eg, financial, personnel) must be made available to facilitate adoption, and the technology must satisfy the educational goals of the institution and needs of the students.	Smith MA, Mohammad RA, Benedict N. Use of virtual patients in an advanced therapeutics pharmacy course to promote active, patient-centered learning. Am J Pharm Educ 2014; 78(6):125
Virtual Patients are a well-known and widely used form of interactive software used to simulate aspects of patient care that students are increasingly less likely to encounter during their studies.[...] While most agree that the use of Virtual Patients in conjunction with traditional teaching methods is associated with large positive effects [10], depending on the type of system being developed, multimedia production can incur relatively high costs if multimedia is used extensively for Virtual Patient creation.[...] However, this lack of access to patients is considered to be " fertile ground" for computer simulations, where numerous cases, each of them a variation of a theme, could be displayed to students to compensate for this lack of real life experience. Therefore, there is much literature that suggests the availability of multiple cases, either real patients or simulations, is advantageous for learning decision-making	Bloice MD, Simonic KM, Holzinger A. Casebook: a virtual patient iPad application for teaching decision-making through the use of electronic health records. BMC Med Inform Decis Mak. 2014;14:66
Virtual patients are computer-based simulators of patient encounters for the purposes of instruction, practice, and assessment. Although virtual patients have been around for some time they have yet to become part of mainstream medical education. A major reason for this would seem to be a lack of clarity as to what educational value virtual patients actually have.[...] Computer-based simulations of patient encounters have been a focus of interest in the medical education literature for some time, often but not always under the label of 'virtual patients'. A systematic review by Cook and Triola in 2009 concluded that 'virtual patients should be designed and used to promote clinical reasoning skills'. An assumption more or less explicit in much of this work is that a virtual patient is an	Ellaway RH. Virtual patients as activities: exploring the research implications of an activity theoretical stance. Perspect Med Educ 2014; 3:266-77

intervention, a catalyst that causally affords different or improved educational outcomes. Virtual patients have been described using this approach; the overall objectives of virtual patients (how they fit into a curriculum or programme of study), the actions constructed around the virtual patient (what teachers and learners do with and around them), and the operations needed to use the virtual patient (the clicks and key presses needed to make them run). In terms of scholarship these levels can equate to inquiry into why virtual patients are used, how they are used and how they work.	
Training for intraoperative technical skills on simulators and for perioperative care in virtual patients have independently demonstrated educational value [...] The only perioperative care training that has been published so far in the educational system is experiential or via lectures but not interactive between the trainees and the patients, that is, that the patients themselves (virtual or actors) provide data about their chief complaint, history, and examination. [..] An attractive field of interactive training is the use of online, 3-dimensional (3D), virtual patients, using pre- and postoperative scenarios. The 3D, virtual patients are defined as multidimensional model patients through which a user has to simulate communication, information gathering, and apply diagnostic reasoning in support of effective and cost-efficient surgical care. Medical education is evolving continually to use such novel internet-based technologies and 3D virtual patients have demonstrated fidelity and content validity. Moreover, virtual patients are uploaded on a virtual world that is easily accessible through an internet connection.	Beyer-Berjot L, Patel V, Acharya A, Taylor D, Bonrath E, Grantcharov T, Darzi A, Aggarwal R. Surgical training: design of a virtual care pathway approach. Surgery 2014;156(3): 689-97.
In an environment where medical education is fast exploring the application of technology to augment, and in some cases to replace classroom activities virtual patients (VP) offer easy and flexible access to training which can suit students' personal preferences and complement other teaching methods. [..] Learners can use a VP to practice repetitively with a standardized scenario in a safe environment, and using the VP feedback, they can reflect on their own experience gained during the interaction. Various VP systems include video combined with voice recognition, conversationwith the VP, on-screen help, and learner feedback, as well as links to additional teaching materials. The performance in VP and SP encounters correlate, providing evidence of validity for the VPs use as educational tools. VPs may be particularly suitable for convergent learners who value technological applications of knowledge. VPs have been shown to be beneficial in teaching various mental health-related topics and skills, for example, alcohol use screening and brief intervention, obtaining consent for antipsychotic prescriptions, or nursing care of distressed psychiatric patients.	Foster A, Chaudhary N, Murphy J, Lok B, Waller J, Buckley PF. The Use of Simulation to Teach Suicide Risk Assessment to Health Profession Trainees-Rationale, Methodology, and a Proof of Concept Demonstration with a Virtual Patient. Acad Psychiatry 2014;
Virtual patient (VP) cases are an effective teaching method, although little is known about how to design and implement them for maximum effectiveness. [...] A growing branch of e-learning is the use of virtual patients (VPs). VPs are becoming particularly popular for teaching clinical reasoning, due to the interactive learning experience, which can simulate some of the diagnostic steps and the clinical decision-making processes of physicians' daily work. Additionally, they have been shown to be effective at propagating knowledge. Many different approaches and programs for designing VP cases, including different structures and layouts, are currently available. [..] In order to optimize the effectiveness of VP cases, design and implementation play an important role. Regarding implementation, it seems that relevance to seminars and corresponding tests, as well as a smooth,	Jäger F, Riemer M, Abendroth M, Sehner S, Harendza S. Virtual patients: the influence of case design and teamwork on students' perception and knowledge - a pilot study. BMC Med Educ 2014; 14:137.

balanced and functional integration into the curriculum, are important in order to motivate students to actually use the cases. [...] Another important aspect is teamwork, which has been shown to activate learners and to enhance knowledge and, therefore, might also be relevant for learners' motivation and retention of knowledge when working with VPs. In a study by Edelbring et al., students reported a perceived benefit to their clinical reasoning skills from working on VP cases with a partner and discussing their patient management approaches. Furthermore, motivation has been identified as being a dependent variable influenced by autonomy, which is also a necessary skill when working on VP cases.	
To that end, there has been increasing use of virtual patients. The American Association of Medical Colleges defines virtual patient as a ''specific type of computer program that simulates real-life clinical scenarios.'' Similarly, Ellaway defines it as ''an interactive computer simulation of real-life clinical scenarios, for the purpose of healthcare and medical training, education or assessment.'' As such, virtual patients are a specific type of clinical simulation that differs from other types of simulation, such as simulated patients/clients and skill-based manipulative simulators. Kneebone11 cites four main advantages of clinical simulations, including that the training agenda can be determined by the needs of the learners, rather than by the cases presented to them in a hospital setting, and that students can learn in an environment where it is ''safe to fail.'' In addition, clinical simulations can provide objective evaluations of performance and immediate feedback for the learner. The research on virtual patients in particular suggests that they lead to more positive educational outcomes when added to a curriculum and that they are particularly well-suited to teaching clinical reasoning competencies since they allow for the ''application of knowledge to collect and integrate information from various sources to arrive at a diagnosis and management plan.' [...]Furthermore, virtual patients provide learners with the opportunity for deliberate practice in clinical reasoning skills, especially when they are structured so as to include effective, real-time, formative feedback and are integrated effectively into a clinical curriculum through appropriate sequencing and progressive difficulty of cases. Furthermore, virtual-patient technology offers the opportunity for a much more varied and accessible range of case scenarios for learners and greater opportunities for educators to collaborate and share the cases they have developed.	Byron JK, Johnson SE, Allen LC, Brilmyer C, Griffiths RP. Development and pilot of Case Manager: a virtual-patient experience for veterinary students. J Vet Med Educ. 2014; 41(3):225-32
Since their inception, virtual patients have provided health care educators with a way to engage learners in an experience simulating the clinician's environment without danger to learners and patients. This has led this learning modality to be accepted as an essential component of medical education. [...] Virtual patients have been defined as "interactive computer simulations of real-life clinical scenarios for the purpose of medical training, education, or assessment" by the MedBiquitous Consortium for the development of health care technology standards. The need for streamlining the virtual patient creation process became apparent and standardization solutions were offered with a formal MedBiquitous virtual patient initiative for the smooth exchange of virtual patients across systems and institutions being finalized in a formal International Standard form since 2010. Virtual patients, with current Web-based rapid development and deployment cycles, can be ubiquitously present in the curriculum (lectures, exams, project-problem-based learning, synchronous or asynchronous e-learning sessions).	Antoniou PE, Athanasopoulou CA, Dafli E, Bamidis PD. Exploring design requirements for repurposing dental virtual patients from the web to second life: a focus group study. J Med Internet Res 2014; 16(6): e151.

This proliferation of virtual patients has led to attempts of highly specialized, context-specific virtual patient design models for catering to specific medical specialties, or the use of virtual environments' immersiveness by deploying virtual patients in environments such as Second Life	
virtual patient case simulation are documented educational techniques that have had success in preparing students to focus learning on core information that is relevant to real clinical scenarios and adaptive feedback.	Al-Dahir S, Bryant K, Kennedy KB, Robinson DS. Online virtual-patient cases versus traditional problem-based learning in advanced pharmacy practice experiences. Am J Pharm Educ 2014;78 (4):76
Virtual patients (VPs) offer valuable alternative encounters when live patients with rare conditions, such as cranial nerve (CN) palsies, are unavailable; however, little is known regarding simulation and optimal social learning context. [..] Virtual patients (VPs) presented through computer-based systems offer valuable educational encounters due to their capacity to mimic a variety of complex pathologies and clinical scenarios. Computer-based VP simulations are affordable, widely distributable and can be made accessible on demand, allowing for student engagement independent of, or supplemental to, classroom and clerkship experiences. [...] Effectively integrating simulation experiences into the curriculum presents its own set of challenges (e.g. availability of expert faculty members, educational space, equipment and allowable student contact hours).	Johnson TR, Lyons R, Kopper R, Johnsen KJ, Lok BC, Cendan JC. Virtual patient simulations and optimal social learning context: a replication of an aptitude-treatment interaction effect. Med Teach 2014; 36(6): 486-94
Virtual patients (VPs) have been proposed as a learning activity that can support nursing students in their learning of scientific knowledge and help them integrate theory and practice. Although VPs are increasingly used in health care education, they still lack a systematic consistency that would allow their reuse outside of their original context. [...] A type of computer-based simulation called virtual patients (VPs) has been proposed to support nursing students in their acquisition of scientific knowledge as a way to integrate theory and practice and promote clinical reasoning. [...]Virtual patients (VPs) have been proposed as a learning activity that can support students to integrate theory and practice in their development of clinical reasoning skills VPs can take many different forms and can be realized using a wide range of presentations, styles, and configurations. There are also several definitions for the concept of the virtual patient, and the term VP is often used in an ambiguous manner. In this study, we adopted Ellaway's definition: "An interactive computer simulation of real-life clinical scenarios for the purpose of health care and medical training, education or assessment". VPs can be designed in different ways and are often dependent on the technical affordances of the system used to author them. Most VPs have common features including medical history taking, physical examinations, lab/imaging tests, as well as features for suggesting an appropriate diagnosis and treatment. An essential characteristic of VPs is the interactive interface that enables the user to query the patient and receive a patient response supplied by the computer. A particular strength of VPs is that they seem to support learning on clinical reasoning and decision making. Students may be exposed to a large number of VP cases in a safe and controlled environment. Clinical learning experience is difficult to standardize and schedule in a reproducible manner. However, VPs can provide exactly the same	Georg C, Zary N. Web-based virtual patients in nursing education: development and validation of theory-anchored design and activity models. J Med Internet Res 2014;16(4): e105

experience repeatedly and also allow students to revisit their actions during the interaction with the virtual patient, and then compare them with the best practice protocol. VPs also facilitate a venue for safe and repetitive practice and stand as a model where progressive clinical variation and difficulty can be presented [28]. Studies also show that VPs are a cost-effective way to teach and assess clinical skills and clinical reasoning among medicaql students	
Virtual Clinical Encounters (VCEs), was suggested to supply more targeted individualized training in clinical interviewing skills. VCEs, featured by Virtual Patients (VPs), are advanced, contextualized and simulated learning environments which portray reallife clinical encounters and have already been proven efficient and cost-effective complementary educational tools<br>in healthcare.	Courteille O, Josephson A, Larsson LO. Interpersonal behaviors and socioemotional interaction of medical students in a virtual clinical encounter. BMC Med Educ 2014;14:64
Virtual patients are increasingly common tools used in health care education to foster learning of clinical reasoning skills. [...] The use of virtual patients is undeniably one of the techniques most often associated with application of computer-aided training in health care but, as is often the case with new concepts, the understanding of the term "virtual patient" varies depending on the research community. We define a virtual patient as "interactive computer simulation of real-life clinical scenarios for the purpose of health care and medical training, education or assessment". This excludes other methods used in medical education such as human role-playing, computerized mannequins, part-task trainers, and systems requiring specialized<br>equipment, as well as all non-educational virtual patients. While complex, immersive virtual reality scenarios are technologically possible, the routine use of virtual patients often focuses on technically simple solutions. [...]<br>There are at least two significant advantages of this type of virtual patient. The first is the possibility of significant teacher involvement in the development process. This has been enabled by investment in the user-friendliness of authoring tools and the simplification of technical workflows to enable medical experts to focus on the content of virtual patients. As a result, virtual patients of this class are generally tailored to the needs of a particular teacher and institution, to the type of educational activity, and to the specific learning objectives. This might not be the case with technologically complex virtual patients. The second advantage is the high level of accessibility of this type of virtual patient to learners over the Internet with personal computers, or even "just-in-time" access with mobile devices. Accessibility is also enhanced by the low cost of licensing of these virtual patient systems.<br>[..] While these systems differ in many respects, they have in common the presentation of a clinical, case-based scenario divided into discrete steps displayed either (1) linearly, with a single final outcome, or (2) branched, enabling different narration paths depending on user choice.	Kononowicz AA, Narracott AJ, Manini S, Bayley MJ, Lawford PV, McCormack K, Zary N. A framework for different levels of integration of computational models into web-based virtual patients. J Med Internet Res 2014;16(1): e23
Virtual patients (VPs) may support learning processes and be a valuable complement in teaching communication skills, patient-centeredness, clinical reasoning, and reflective thinking. [...] In a review, Cook and Triola defined virtual patients as a "specific type of computer programme that simulates real-life clinical scenarios; learners emulate the roles of health care providers to obtain a history, conduct a physical exam, and make diagnostic and therapeutic decisions". Virtual patients (VPs) are currently introduced into many health care programs worldwide but are still sparsely used in medical school curricula. Designing a VP is a delicate process, and the production of	Salminen H, Zary N, Björklund K, Toth-Pal E, Leanderson C. Virtual patients in primary care: developing a reusable model that fosters reflective practice and clinical reasoning. J Med Internet Res 2014;16(1): e3

learning objects for multimedia consumes both time and resources. VPs are found to be useful in teaching students clinical reasoning skills. [...] Examples are an important part of clinical reasoning, and VPs have something to contribute here as a complement to the real-life clinical practice	
Virtual patients have been found to be a dynamic and costeffective means of providing nursing students with standardized clinical simulations. Virtual patients create an objective learning environment by presenting each student with an identical simulation scenario. Virtual patients provide students with a more uniform opportunity to assess their skills than standardized patient actors, who can insert biases or become bored with the repetition of performing the same simulation repeatedly with multiple students. Another advantage of virtual patients is a more convenient simulation experience that can often be accessed asynchronously, which makes them ideal for distance education programs. Virtual patients are relatively new in the field of clinical simulations; however, their low cost in comparison with simulations using high-fidelity manikins or patient actors makes them appealing in the field of nursing education, where budgets are typically tight, and there is a shortage of educators. [..] The use of virtual patients in health professions education has been applied to a wide range of clinical fields. Although, in most cases, the learning objectives of virtual patient simulations include the development or assessment of clinical reasoning skills, Cook and Triola have made the argument that demonstrating clinical reasoning is the only valid learning objective for a virtual patient simulation.	Kleinheksel AJ. Transformative Learning through Virtual Patient Simulations: Predicting Critical Student Reflections. Clinical Simulation in Nursing 2014; 10: e301-8
Today, there is software available for students to interact with a VP online, in a simulated clinical environment. Interestingly, VPs have been around for over 35 years; however, the technology has not been widely utilized, and there is a lack of research confirming learning outcomes. Nonetheless, VP technology has advanced considerably in recent years, is comparable to advanced video gaming systems, and interactivity of artificial intelligence. Today, students can interact with a VP online with text or with their voice, choose how to proceed in a virtual clinical environment, and receive immediate feedback on their performance. [..] Students can engage with a VP in experiential learning through an unfolding simulated case. There is no time pressure to complete a case, so students may pause, reflect, and choose alternative paths and decisions. After completion of a case, students receive immediate computerized feedback on their performance. Students have the opportunity to repeat their practice and gradually refine their performance. This deliberate practice cycle is a sequence of practicing through repetition, and providing feedback, with the opportunity to improve. As students dig deeper to discover the content embedded in each case, they engage and assume more responsibility for learning. Most importantly, a VP experience allows nurses to learn in a safe, virtual environment in which time can be paused. Overall, the pause allows time for self-analysis. This ability to take time to reflect on one's clinical performance, and evaluate strengths and weaknesses, plays a major role in becoming a safe practitioner and demonstrating clinical judgment and reasoning skills. VPs are well suited for practicing RNs, as they require application of knowledge, while at the same time building upon previously learned knowledge and experience. RNs are challenged in the unstructured virtual clinical environment; whereas novice students may benefit more from a sequential problem based learning module.	Friedman SA, Goldschmidt K. Let me introduce you to your first virtual patient. J Pediatr Nurs 2014; 29: 281-3

In a seminal paper Cook et al. suggested virtual patients (VPs) as the key technology to enhance the skills of clinical reasoning. VPs defined as “interactive computer simulations of real-life clinical scenarios” have potential features to remedy some of the problems addressed currently in MOOCs, enabling interactivity, experiential learning and fostering specific medical skills.	Stathakarou N, Zary N, Kononowicz AA. Virtual patients in massive open online courses-design implications and integration strategies. Stud Health Technol Inform 2014:793-7
Virtual patient (VP) cases are interactive computer simulations of real life scenarios that have been used in medical education for over a decade. They are popular with students and staff alike and have been shown to improve knowledge retention, clinical reasoning and decision-making skills. Ethics virtual patient (EVP) cases are interactive computer simulations of real life scenarios which have a substantive ethical component. They can also contain significant legal and professionalism components. EVP cases have only recently been used in medical education, but there is growing evidence to suggest that medical students find them interesting, engaging and helpful.[...] An electronic virtual patient (VP) can be defined as ‘an interactive computer simulation of real-life clinical scenarios’. VPs have been used in medical education for over a decade and they enable teachers to create richly layered, multidimensional and interactive teaching environments. Students using a VP simulation must apply knowledge to make decisions so that they can progress through the case. If the cases are truly reflective of real situations and choices, they can be excellent tools for practicing reasoning and decision-making skills. They also enable students to make (and learn from) mistakes in a safe environment. [...] The key problem with the use of VPs in the past was the expense of creating them. However, the costs of the technology used to create VPs and EVPs—for example DecisionSim, OpenLabyrinth and VUE—has decreased rapidly of late. Another key problem in the past was the need for specialist e- Learning and programming experts to help create the VPs. However, these problems are also becoming less acute because the available technology is now very user friendly and can be used much more easily by people who lack formal training in e-Learning.	Hooper C. Ethics virtual patients: a new pedagogical tool for educators? J Med Ethics 2014;
Virtual patients (VPs) are increasingly used to train clinical reasoning. So far, no validated evaluation instruments for VP design are available.[...] They have been defined as ‘‘interactive computer simulations of real-life clinical scenarios for the purpose of medical training, education, or assessment’’, and seem to be especially suited to fostering clinical reasoning. VP design is essential for the educational success of VPs. The inherent costs of developing VPs underline the importance of paying careful attention to VP design. Together with the increasing use of VPs both nationally and internationally, within the scope of the ‘‘electronic Virtual Patients’’ (eVIP) project, these arguments led us to the realization that a short and standardized evaluation tool of VP design is a prerequisite for achieving further expansion of VP use in medical education.	Huwendiek S, De Leng BA, Kononowicz AA, Kunzmann R, Muijtjens AM, Van Der Vleuten CP, Hoffmann GF, Tönshoff B, Dolmans DH. Exploring the validity and reliability of a questionnaire for evaluating virtual patient design with a special emphasis on fostering clinical reasoning. Med Teach 2014; 1-8, Early Online
Virtual patients (computer-based simulated clinical encounters) have been around for several decades but they have only recently started to enter mainstream medical education. Virtual patients can take many forms and they can be used within different kinds of educational activities. While virtual patients for self-directed learning function as the primary instructional device, virtual patients for D-PBL do not; they scaffold the activity and provide the learners with triggers as the case unfolds but the learning is realized more through participants’ discussion and problem solving with each other and in their independent research than through their interactions	Ellaway RH, Poulton T, Jivram T. Decision PBL: A 4-year retrospective case study of the use of virtual patients in problem-based learning. Med Teach 2014; 1–9, Early Online

with the virtual patient.	
There are a number of educational technologies that share certain characteristics with PBL. For instance, virtual patients are on-screen learning resources that typically present a clinical problem for learners to solve or manage, and in doing so involve aspects of both PBL and simulation. Although virtual patients can come in many forms, one of the more common forms is the branched case where learners select the best available course of action from predefined options; each decision the learner makes can have consequences and lead to different outcomes.[...] Virtual patients are interactive computer simulations of "real-life clinical scenarios for the purpose of medical training, education, or assessment". Although virtual patients can take many forms, they are intended usually for individual study.	Poulton T, Ellaway RH, Round J, Jivram T, Kavia S, Hilton S. Exploring the efficacy of replacing linear paper-based patient cases in problem-based learning with dynamic web-based virtual patients: randomized controlled trial. J Med Internet Res 2014; 16(11): e240
The use of simulation as a case-based learning activity to achieve interprofessional education (IPE) objectives is difficult because it is resource-intensive with regard to cost, facilities, faculty time, and aligning curricular and student schedules. Computer-based virtual patients, however, are able to overcome these barriers by allowing small groups of students to meet at their convenience rather than trying to coordinate a single date and time for multiple professions to meet with or without a faculty facilitator.	Shoemaker MJ, Platko CM, Cleghorn SM, Booth A. Virtual patient care: an interprofessional education approach for physician assistant, physical therapy and occupational therapy students. J Interprof Care 2014; 28(4): 365-67
For instance, virtual patients have been used to train medical students in interpersonal communication skills	Rivera-Gutierrez DJ, Kopper R, Kleinsmith A, Cendan J, Finney G, Lok B. Exploring gender biases with virtual patients for high stakes interpersonal skills training. Lecture Notes in Computer Science 2014; 8637:385-96
In a new approach to ultrasound training, complex and expensive physical phantoms are replaced by a 3D virtual patient model, which represents the anatomy of any desired body part or organ, and ultrasonography of the virtual patient with a virtual ultrasound probe is simulated on a standard laptop computer. The advantage to training is not only cost effectiveness, but also the ability to emulate various disease states or conditions in different virtual patients and to visualize the underlying body structures of interest through multiple examination procedures with the virtual probe.	Petrinec K, Savitsky E, Terzopoulos D. Patient-specific interactive simulation of compression ultrasonography. Proceedings - IEEE Symposium on Computer-Based Medical Systems2014;113-8;
Virtual patient cases (VPs) are used for healthcare education and assessment. Most VP systems track user interactions to be used for assessment. [...] The utilization of virtual patient (VP) simulations in medical education has become very common internationally, particularly for teaching clinical reasoning problem solving, and clinical decision making. Virtual patient systems have also been increasingly utilized for self-assessment or for student assessment in many medical and nursing schools. Virtual Patient-based exams are also being used in sections of national board exams including Step 3 of the USMLE Board Exam. Of specific interest is that a VP system can	Fors U, Gunning WT. The Impact of Different Scoring Rubrics for Grading Virtual Patient-Based Exams. Journal of Educational Computing Research 2014;50(1): 97-118.

record all interactions that a learner makes with a VP during an examination to measure development of accurate differential diagnoses and appropriate treatment plans, as well as allowing for automatic grading schemes. In addition, since all actions taken by the learner may be reviewed, an exam that incorporates a combination of construct-related evidence and content-related validity readily allows one to evaluate the learner's complete reasoning process. Most VP applications record learners' individual actions while progressing through a case, including specific medical history questions asked of the virtual patient, the physical exam procedures performed, any laboratory or imaging tests ordered, as well as providing a means for the learner to suggest diagnoses and appropriate therapy decisions. Thus, the learner may be assessed for accurate diagnostic capability and treatment protocols and their approach to a VP may be reviewed to determine diagnostic efficiency, providing insight regarding the rationale for asking specific questions of the VP or for ordering specific laboratory, imaging, or other ancillary medical tests. Using a VP to assess clinical decision making requires that the teacher or examiner select appropriate questions, physical exams and lab/imaging tests from hundreds or even thousands of possible options available when developing an exam case. Therefore, optimal VP "inquiries" can be used to assess the learners' critical reasoning/decision-making skills in terms of how many appropriate and/or unnecessary actions the user performed. A VP-based exam may be created using a real patient's medical record or upon a number of aggregate cases that illustrate different aspects of a particular condition or subject area (e.g., restrictive pulmonary disease in general and hypersensitivity pneumonitis for the specific diagnosis) enabling a more complete assessment of a student's clinical reasoning abilities and skills.	
Researchers have used virtual patients to teach communications skills to medical students, and students have rated the virtual patient experience as being as effective as a standardized patient (actor) [4]. Medical students have also used virtual patients to help practice patient interviewing skills with a high level of immersion. Results indicate that using life-size virtual characters with speech recognition is useful in their education.	
However, the evolving role and growing utilization of virtual patients (VPs) in undergraduate medical education; as well as an increased emphasis on blended learning, multi-modal models that include VPs in core curricula; suggest a growing requirement for strategies or guidelines that directly focus on VPs.[...] A virtual patient (VP) is a computer-based simulation of a clinical scenario for learning and assessment. VPs use authentic, relevant and comprehensive clinical scenarios to actively engage students in problem-solving exercises that emphasize critical analysis, pattern recognition through deliberate practice, require decision-making, hypothesis generation, treatment planning and care management skills , align decision-making with consequences, and allow for necessary repetition and deliberate practice in a safe and controlled environment. They provide necessary immediate, continuous and iterative feedback; as well as access to exemplars of expert practice and rationales. VP cases can integrate distracters such as false or misleading test results, delays and interruptions to further mimic authentic clinical environments. Case complexity can be adjusted to encourage learners to focus on tasks beyond their current levels of competence or comfort without impact on patient safety. When presented as a series of cases, they can support learners in the development of their own experiential knowledge and mental databases.[...] VPs	Posel N, McGee JB, Fleiszer DM. Twelve tips to support the development of clinical reasoning skills using virtual patient cases. Med Teach 2014; 1-6, Early Online

are increasingly embedded within core medical curricula. Several qualitative studies have described the potential of VPs to develop clinical reasoning skills in medicine and nursing to support clinical-management decision-making through feedback; and through authors' case-design decisions, such as branching, to impact on efficacy of learning outcomes, including clinical reasoning. None to date have provided practical guidelines to support the development of clinical reasoning by using VPs.	
Virtual Patients (VPs) have successfully been applied in medical and healthcare education for a number of years. A variety of authoring and playback tools (players) for VPs have been introduced with both similarities and differences with regard to their features. Most players have a number of common aspects such as medical history taking, physical examinations, diagnostic tests, as well as features for suggesting an appropriate diagnosis and treatment. Many players also provide feedback concerning the interactions performed while interacting with the VP. The target users are primarily undergraduate and postgraduate health science students, but postgraduate students and clinicians also use VPs today. Additionally, VPs may also be used for training non-healthcare personnel such as fire-fighters, the police and the military, with the aim of preparing them for interaction with people having various medical conditions. Most VP systems are initially developed at a local university, which influences the language, culture, medical procedures, and scope of cases that are reflected in the respective system. However, in today's society with internationalized healthcare, VPs could play a role in addressing social and cultural diversity. Usually, the VPs reflect common medical conditions found in the region or culture where education takes place. However, there are also indications for using VPs to prepare the learners for meeting less common conditions so as to learn to address uncertainty. This can, for example, be to train European physicians to manage cases such as tropical diseases, or war trauma cases for medical professionals not used to such patients or conditions.	Fors UG, Muntean V, Botezatu M, Zary N. Cross-cultural use and development of virtual patients. Med Teach 2009; 31:732-8
